# Human settlement history between Sunda and Sahul: a focus on East Timor (Timor-Leste) and the Pleistocenic mtDNA diversity

**DOI:** 10.1186/s12864-014-1201-x

**Published:** 2015-02-14

**Authors:** Sibylle M Gomes, Martin Bodner, Luis Souto, Bettina Zimmermann, Gabriela Huber, Christina Strobl, Alexander W Röck, Alessandro Achilli, Anna Olivieri, Antonio Torroni, Francisco Côrte-Real, Walther Parson

**Affiliations:** Department of Biology, University of Aveiro, Campus de Santiago, Aveiro, Portugal; Institute of Legal Medicine, Medical University of Innsbruck, Müllerstr. 44, 6020 Innsbruck, Austria; Cencifor Centro de Ciências Forenses, Coimbra, Portugal; Dipartimento di Biologia e Biotecnologie “L. Spallanzani”, University of Pavia, Pavia, Italy; Dipartimento di Chimica, Biologia e Biotecnologie, University of Perugia, Perugia, Italy; Faculty of Medicine, University of Coimbra, Coimbra, Portugal; Penn State Eberly College of Science, University Park, PA USA

**Keywords:** East Timor (Timor-Leste), Island Southeast Asia, Mitochondrial DNA, mtDNA haplogroup P, Human migration, First settlers, Population genetics, Forensic mtDNA analysis, Next generation sequencing, Ion Torrent PGM

## Abstract

**Background:**

Distinct, partly competing, “waves” have been proposed to explain human migration in(to) today’s Island Southeast Asia and Australia based on genetic (and other) evidence. The paucity of high quality and high resolution data has impeded insights so far. In this study, one of the first in a forensic environment, we used the Ion Torrent Personal Genome Machine (PGM) for generating complete mitogenome sequences via stand-alone massively parallel sequencing and describe a standard data validation practice.

**Results:**

In this first representative investigation on the mitochondrial DNA (mtDNA) variation of East Timor (Timor-Leste) population including >300 individuals, we put special emphasis on the reconstruction of the initial settlement, in particular on the previously poorly resolved haplogroup P1, an indigenous lineage of the Southwest Pacific region. Our results suggest a colonization of southern Sahul (Australia) >37 kya, limited subsequent exchange, and a parallel incubation of initial settlers in northern Sahul (New Guinea) followed by westward migrations <28 kya.

**Conclusions:**

The temporal proximity and possible coincidence of these latter dispersals, which encompassed autochthonous haplogroups, with the postulated “later” events of (South) East Asian origin pinpoints a highly dynamic migratory phase.

**Electronic supplementary material:**

The online version of this article (doi:10.1186/s12864-014-1201-x) contains supplementary material, which is available to authorized users.

## Background

**Figure 1:**
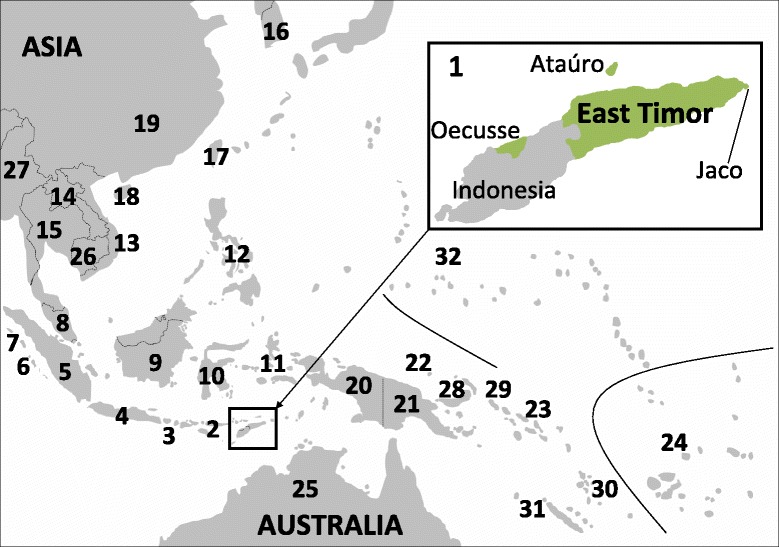
**The geographic location of East Timor and the populations included in this study.** 1 - East Timor, 2 - Nusa Tenggara, 3 - Bali, 4 - Java, 5 - Sumatra, 6 - Mentawai, 7 - Nias, 8 - Peninsular Malaysia, 9 - Borneo, 10 - Sulawesi, 11 - Moluccas, 12 - Philippines, 13 - Vietnam, 14 - Laos, 15 - Thailand, 16 - South Korea, 17 - Taiwan, 18 - Hainan, 19 - Mixed Han (China), 20 - WNG, 21 - PNG, 22 - Admiralty Islands, 23 - Solomon Islands, 24 – Polynesia/Fiji, 25 - Australia, 26 - Cambodia, 27 - Myanmar, 28 - New Britain and New Ireland (Bismarck Archipelago), 29 - Bougainville, 30 - Vanuatu, 31 - New Caledonia, 32 - Micronesia, 33 - New Zealand Maori (not on map). Populations 2–7, 9–11, and 20 are in Indonesia; 22, 28 and 29 in PNG. The population codes were retained throughout this study. For references see Additional file 11 where subpopulations are indicated with suffixes.

The Democratic Republic of Timor-Leste (East Timor) is located in the Lesser Sunda Islands (Nusa Tenggara) of Island (or Maritime) Southeast Asia (ISEA), between Mainland Southeast Asia (MSEA) and Australia, the Indian and the Pacific Ocean. It extends over the eastern part of Timor, the adjacent islands Ataúro and Jaco, and Oecusse, an exclave within western Timor (Indonesia) (Figure 1). Under Portuguese rule since the 16^th^ century, East Timor declared its independence in 1975 and obtained it in 2002 after Indonesian occupation. The country has a population of ~1.1 million, a total area of 14,919 km^2^ and its highest peak reaches 2,963 meters. Thirty-two languages are spoken in the 13 districts; four working languages are used [1,2]. Archaeological, ethnographic, linguistic and genetic investigations of ISEA have outlined colonization on the crossroads of multiple migrations between today’s (S)EA mainland, Australia and the Pacific islands. The area of eastern Indonesia (including East Timor) has been described as a “melting pot” and a migratory “highway” based on genetic data [3-5]. Initial DNA studies have provided insights into East Timor’s complex composition [3,6-12].

The routes and timing of human dispersal in the area remain to be fully clarified (*cf*. [13]). In any assumption, the influence of a changing climate needs to be considered. The sea level in ISEA rose after ~70 thousand years ago (kya), then sank from ~30 kya during the last glacial maximum (LGM) until ~18 kya, whereupon the landmasses of Sunda(land) (*i.e.* Sumatra, Java, Borneo, the Malay Peninsula and nearby islands) and Sahul (or Meganesia; *i.e.* Australia, New Guinea, Tasmania and nearby islands) became partly submersed again by the rising sea. Dramatic coastline shifts occurred ~14.5, 11.5, and 7.5 kya, creating the extant archipelago topography [4,5,14-16]. Timor was an island even during the LGM.

The pioneer migration out of Africa brought the first anatomically modern humans, likely hunter-gatherers and beachcombers able to perform (short) voyages, to Sahul ~60-40 kya [5,17-21]. The expansion from Sunda to Sahul might have occurred along northern (via Sulawesi) or southern Wallacea (via Nusa Tenggara and Timor) [5,14,15,20,22-28]. The so-called Austro-Melanesian, “negrito”, or Australoid first settlers are associated with non-Austronesian (*i.e.* Papuan) languages, spoken as far east as the Solomon Islands [15,24,29-31]. Their initial “fast train” migration likely left individuals carrying deep-rooting mtDNA founder haplotypes along its course. Autochthonous mtDNA lineages of ISEA, Melanesia and Australia support a long-term *in situ* development and date the first modern human arrival to >48 kya [3,5,14,26,28-30,32-34]. The presence of Upper Paleolithic settlers in the region is confirmed by findings in East Timor (>42 kya) [20,27], Borneo (~46 kya) [33], eastern Papua New Guinea (PNG) (~43-49 kya) [35], Melanesia and Australia (≥48 kya) [3,5,25,30,36]. Submergence could have caused a scarcity of sites (*cf.* [37,38]). Subsequent migrations led to admixture with or replacement of the present foragers and shaped the genetic diversity of ISEA. A major southward expansion out of Taiwan is postulated from mid-Holocene (~8-4 kya) linguistic and cultural changes associated with the spread of livestock domestication, agriculture and Austronesian (AN) speakers. Their arrival in eastern Indonesia (and Timor) is estimated to ~4 kya from Neolithic findings. This migration has found support by dispersal patterns of mtDNA lineages with ample basal diversity in Taiwan, and is connected to the East Asian (EA) proportion of the genetic pool in ISEA and AN speaking populations [3,5,15,24,28-30,39-42]. However, the so-called “out of Taiwan” (OOT) event has been questioned both in linguistics as single source of AN languages [30] and in genetics, since the model does not predict a direction and AN speakers share few mtDNA lineages despite matrilocality [5,43]. Moreover, archaeobotanic research indicates plant domestication in ISEA before this presumed advent [16]. A two-step arrival, ~60-40 kya and ~8-4 kya, does not appear to fully reflect the demographic history of the region [24,44]. MtDNA coalescence ages, phylogenetic and dispersal patterns that neither fit with long term *in situ* development >40 kya nor southward population expansions <8 kya indicate a broader timeframe and geographic origin of migrations [5,28,30]. Additional mid-Holocene expansions, labelled “express train” and “slow boat” [28,45], might have originated elsewhere, possibly within ISEA, MSEA or Near Oceania [3,5,24,28,30]. To account for these inconsistencies and to integrate the expected effects of a changing environment, such as facilitated migration by exposed and forced displacement by submersing land [15,24], a more comprehensive model needs to be outlined to explain the extant maternal genetic landscape of ISEA. The classical “waves” might rather represent longer periods of migration than short, distinct events. The second “wave” may be extended to a period of ongoing gene flow in the late Pleistocene and early Holocene between ~40/30-10/5 kya, with substantial, recurrent population expansions and shifts between Asia and Sahul and within ISEA, that may include the dispersals postulated from lineage-specific investigations [5,15,24,28,30,40,42,46]. These intermediate migrations are supported by archaeological, linguistic, Y-chromosomal and autosomal DNA evidence ([5,16,30]: “early train”). Further complexity might be added by gene flow from India into Australia ~4.2 kya [47] and “historic” movements from China, Arabia and India associated with trade and the spread of religions [3,5].

Even in the classical scenario, East Timor is of high genetic interest: it could have accommodated the “last step” before the colonization of Sahul, westward (back) migrations have been suggested, and the southward OOT migration could have extended there [3,5,27,28,40,48]. Nevertheless, mtDNA composition of its population is scarcely described: the available data comprise 38 hypervariable segment I (HVS-I) sequences [3] and 133 not individually reported haplotypes [10]. We here present the first representative complete mtDNA control region (CR) reference dataset for East Timor, comprising 324 country-wide samples (Additional files 1 and 2) sequenced according to highest forensic quality standards. Our aim was to explore the potential of this geographically restricted sample in providing insights into the history of (particularly the less investigated initial) human dispersal into and over the entire region. We thus also generated complete mitogenome sequences of 17 samples and fundamentally refined the phylogeny of mtDNA haplogroup P1 that appeared most promising for phylogeographic reconstructions. We used the Ion Torrent Personal Genome Machine (PGM) on an mtDNA population sample in a forensic environment as stand-alone approach for the first time; this study therefore significantly contributes towards the implementation of massively parallel sequencing (MPS) into high quality mtDNA typing routine.

## Results and discussion

### Ample mtDNA variation of the East Timor population

**Figure 2:**
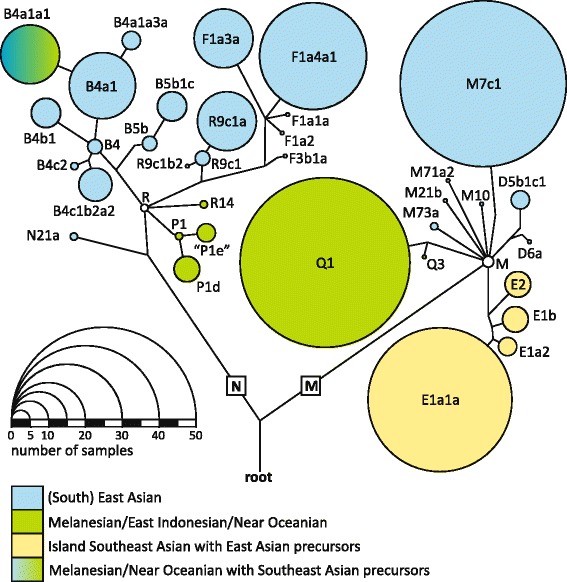
**Schematic phylogenetic tree of all haplogroups observed in the 324 East Timor samples.** Their phylogenetic relation and postulated geographic origin are indicated. The sizes of the circles correspond to haplogroup frequencies. Stem lengths are of no information content. The tree is rooted in the MRCA. All information available was used. Haplogroups are according to [49], build 16.

The diverse haplogroup spectrum of East Timor revealed in this first representative mtDNA study illustrates its position at the crossroads of several migrations between ISEA, Melanesia and Australia – and likely also the area’s rapid change from a large continental landmass to an archipelago [4]. We detected 164 different CR haplotypes in the 324 mtDNAs (disregarding cytosine indels around nps 16193, 309 and 573); 116 of these (35.8%) were unique in the dataset. Macro-haplogroup M comprised 50.3% of the samples in 14 haplogroups; macro-haplogroup N 49.7% in 22 haplogroups (Table 1). Most frequent were Q1 (14.2%) and M7c1 (13.9%). Using all information, 11.1% of the samples could be assigned to a terminal “twig” of the mtDNA Phylotree ([49], build 16) (10.8% based on CR). The most frequent CR haplotypes, relative to the revised Cambridge Reference Sequence (rCRS) [50], were 73G 146C 199C 263G 309.1C 315.1C 489C 523del 524del 16223T 16295T 16362C 16519C (haplogroup M7c1) and 73G 152C 249del 263G 309.1C 315.1C 521del 522del 523del 524del 16129A 16172C 16294T 16304C 16362C 16519C (haplogroup F1a4a1), with 4.0% each (Additional file 3). Figure 2 depicts the proportions of haplogroups, their phylogenetic relations and postulated geographic origin. The latter reflects influence from two geographic macro-regions: mainly (S)EA lineages were detected (56.5%; haplogroups B, D, F, M7, M10, M21, M71, M73, N21, R9). A considerable proportion indicated expansions of EA precursors in(to) ISEA and Polynesia (22.8%; haplogroups B4a1a1, E), followed by mtDNAs with indigenous Melanesian/eastern Indonesian/Near Oceanian origin (19.4%; haplogroups P, Q, R14). Admixture from outside eastern Eurasia could only be concealed among the unresolved M* and R* mitogenomes (1.2%). The 324 novel East Timor mtDNA haplotypes (307 CR sequences, 17 complete mitogenomes) are illustrated in Additional files 3 and 4, available in GenBank [KJ655583-KJ655889, KJ676774-KJ676790; http://www.ncbi.nlm.nih.gov/genbank] and included in the European DNA Profiling Group MtDNA Population Database (EMPOP) [EMP00534; http://empop.org] [51].

**Table 1:** **MtDNA haplogroup frequencies in our East Timor sample**

**Haplogroup**	**n**	**Frequency (%)**
B4*	4	1.2
B4a1*	18	5.6
B4a1a1	16	4.9
B4a1a3a	5	1.5
B4b1	8	2.5
B4c1b2a2	9	2.8
B4c2	2	0.6
B5b*	4	1.2
B5b1c	8	2.5
D5b1c1	5	1.5
D6a	1^#^	0.3
E1a1a	39	12.0
E1a2	5	1.5
E1b	7	2.2
E2	7	2.2
F1a1a	1	0.3
F1a2	1	0.3
F1a3a	16	4.9
F1a4a1	29	9.0
F3b1a	1	0.3
M*	2	0.6
M7c1	45	13.9
M10	1	0.3
M21b	1^#^	0.3
M71a2	1	0.3
M73a	2	0.6
N21a	2	0.6
P1*	2	0.6
P1d	7^#^	2.2
“P1e”^§^	5^#^	1.5
Q1	46	14.2
Q3	1^#^	0.3
R*	2	0.6
R9c1*	3	0.9
R9c1a	15	4.6
R9c1b2	1	0.3
R14	2	0.6
**Total**	324	100

^#^classified after complete mitogenome sequencing.

^§^postulated novel clade.

n - number of mtDNAs. Haplogroups according to [49], build 16.

### New insights from complete mitogenomes

**Figure 3:**
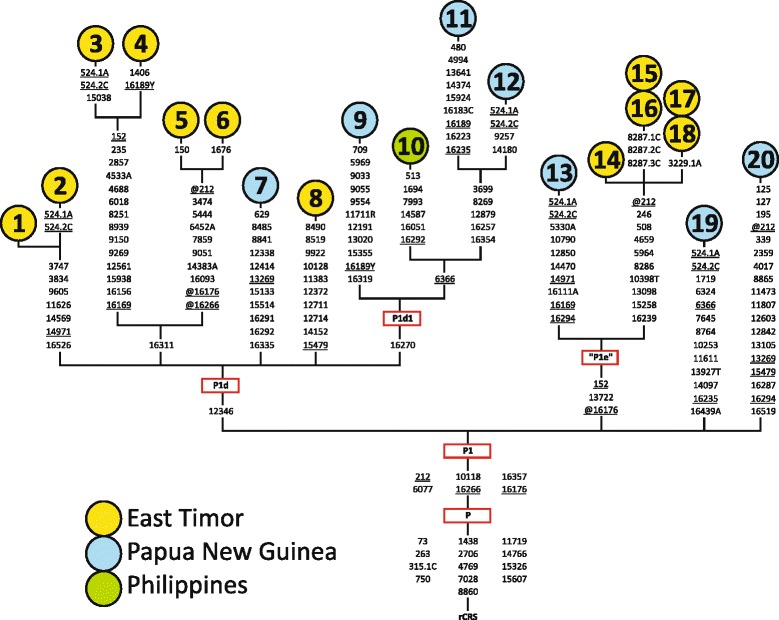
**The refined phylogeny of haplogroup P1.** The 12 novel completely sequenced haplogroup P1 mitogenomes from East Timor are shown together with the eight previously published. All differences are indicated (disregarding cytosine insertions after nps 309 and 16193) relative to the rCRS [50]. Haplogroups are according to [49], build 16. “P1e” is a novel suggestion. Bases are indicated according to the IUBMB nucleotide code. The prefix @ indicates the reversion of a mutation occurring earlier in the phylogeny. Underlined mutations are recurrent in the tree. For details and references, see Additional file 5.

Our analyses at the highest resolution substantially contributed to elucidate and refine the phylogeny of haplogroup P1. This is not “monotypic” as previously described ([49], build 16), which would be uncommon for a successful founder. The most parsimonious tree reconstructed from the available 20 (thereof 12 novel) mitogenomes ([52-55], this study) confirmed subclade P1d and added considerable internal variation to P1d1 with five additional basal branches (Figure 3, Additional file 5). Five East Timor samples and a PNG singleton [52] defined a novel branch characterized by the motif P1-152-13722-@16176 (relative to the rCRS [50]) that we tentatively called “P1e”, expanding the current mtDNA nomenclature [49]. The East Timor P1e quintet additionally shared the transition np 8286, resulting in a previously studied polycytosine stretch (*e.g.*, [56]). Two mitogenomes [52,53] indicated additional basal P1 branches. The earlier proposed subclades P1a-c (*cf*. [48]) were defined partly by homoplasic markers and group with P1d and P1e lineages of our novel phylogeny. Multiple back-mutations of transitions currently defining haplogroup P1 ([49], build 16), viz. nps 212, 16176, and 16266, indicate the need for an updated nomenclature relying solely on the transition at np 16357 as CR marker.

The five completely sequenced non-P1 mitogenomes revealed novel lineages within haplogroups D6a, M21b, M73a, Q3, and R9c1b2 (Additional file 6).

### The genetic position of East Timor

Based on HVS-I and HVS-II data, East Timor (29%) was amid the other island population in the unique haplotype proportions (8-47%, median 45.5%) and the intra-population random match probabilities (RMPs) (1:37–1:67, East Timor: 1:59). Mainland populations yielded higher proportions of unique haplotypes (median: 58.5%) and lower RMPs, likely due to smaller sample sets from islands, colonization bottlenecks and founder effects/isolation (*cf*. [41]). In total, 18.6% of the East Timor haplotypes (comprising 42.6% of the samples) were also found in other populations. The Philippine population shared 18.1% of its haplotypes (33.1% of its samples) with East Timor, followed by Taiwan (12.7|23.3%), all other populations were ≤8.6|6.3%. East Timor ranked second (10.70) in the intra-population numbers of mean pairwise differences (MPD) (9.08 (Philippines) - 11.88 (PNG)) (Table 2, Additional file 7). The inter-population MPD maxima of 13.52 (uncorrected) and 2.46 (corrected) derived from comparisons with the PNG sample. For East Timor, the inter-population MPD were found highest also with PNG and lowest with the Philippines. The pairwise *F*_ST_ values for East Timor indicate a pronounced genetic differentiation if compared with PNG (0.12) and minor ones with all other populations (≤0.05). The values including PNG were 0.12-0.20, while all other comparisons yielded ≤0.05. AMOVA revealed that the observed genetic variation was mainly attributable to variance within populations (95.1%) (Additional file 8).

**Table 2:** **Genetic diversity of the East Timor population and 10 surrounding populations using HVS-I and HVS-II**

**Population**	**Reference**	**n** ^**a**^	**hts** ^**b**^	**Unique hts**	**MPD** ^**c**^	**RMP** ^**d**^
1A - **East Timor**	this study	324	145	93 (29%)	10.705 ± 4.886	0.017
8 - Peninsul. Malaysia*	[57]	205	152	115 (56%)	10.353 ± 4.743	0.009
12 - **Philippines**	[28]	130	83	61 (47%)	9.080 ± 4.206	0.023
13 - **Vietnam**	[58]	187	153	134 (72%)	10.104 ± 4.637	0.009
14 - **Laos**	[59]	214	163	130 (61%)	10.185 ± 4.670	0.009
15 - **Thailand**	[60]	190	133	103 (54%)	9.962 ± 4.576	0.012
16 - **South Korea**	[61]	692	471	378 (54%)	9.372 ± 4.309	0.005
17 - **Taiwan**	[45]	640	122	51 (8%)	9.642 ± 4.425	0.027
18 - **Hainan**	[62]	290	137	74 (26%)	10.315 ± 4.721	0.015
19 - **Mixed Han (China)**	[63]	262	242	228 (87%)	10.478 ± 4.792	0.005
21 - **Papua New Guinea**	[34,64]	142	85	48 (34%)	11.881 ± 5.408	0.016

*(Singapore).

^a^number of mtDNAs.

^b^number of haplotypes.

^c^mean number of pairwise differences.

^d^random match probability.

Reading frame: nps 16080–16193, 16194–16365, 73–300; total sample size: n = 3,276. For geographic locations, see Figure 1.

**Figure 4:**
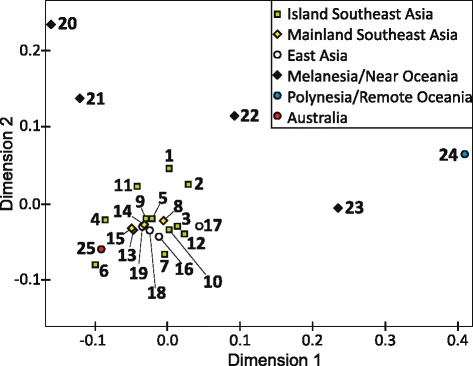
**MDS plot displaying the genetic distances between East Timor and 24 surrounding populations.** Based on pairwise *F*_ST_ values in HVS-I (nps 16080–16180, 16195–16354). For population codes, see Figure 1. For details and references, see Table 3, Additional file 10.

Restricting the comparisons to HVS-I to expand the population number, East Timor (15%) again positioned among the island populations (except Borneo) in the unique haplotype proportions (median: 14.5%). The values for mainland populations, except Australia and Peninsular Malaysia, were generally higher also here (median: 45.5%). East Timor yielded an RMP of 1:26, similar to other ISEA populations (Bali, Java, Sulawesi, and the Philippines) (Table 3), and 54.3% of its haplotypes (comprising 74.0% of the samples) were also found in the 24 surrounding populations. In general, I(S)EA and Oceanian populations shared greater proportions with East Timor than Mainland Asians. The Moluccas ranked highest (41.7%) in the shared haplotype proportions and second in the proportions of individuals, where Polynesia was highest (72.1%). This and similarly high values in other Oceanian populations were caused by the outstanding prevalence of shared B4a1(a1) haplotypes. The influence of greatly differing haplotype numbers and haplogroup spectra (see below) is also visible for Nusa Tenggara and New Guinea (NG) that shared rather low (~10%) haplotype proportions with East Timor compared to the other (geographically more distant) ISEA populations, but large sample proportions (49.6% resp. 24.9%). Australia ranked lowest in both aspects (4.0|9.6%) (Additional file 9). The highest numbers of intra-population MPD were obtained from NG (7.04), Polynesia was lowest (1.79). East Timor again ranked high (5.98), closest to Nusa Tenggara (5.82). The inter-population MPD maxima for HVS-I were 9.59 (uncorrected) and 5.18 (corrected). For East Timor, the lowest (uncorrected) number of inter-population MPD was calculated with Sulawesi (5.57) and the highest with WNG (8.16). Based on the corrected numbers, MPD of East Timor were lowest with the Moluccas (0.04) and Nusa Tenggara (0.07); the remaining values were more than double, up to 2.12 (Polynesia). AMOVA revealed that 87.6% of the variation was attributable to differences within populations (Additional file 10). We used the pairwise HVS-I *F*_ST_ values (discussed in Additional file 10) for an MDS analysis to depict genetic distances between populations (Figure 4). A limited genetic differentiation indicating high gene flow between most (S)EA populations was reflected by the resulting large main cluster. East Timor located in a separate cluster with the Nusa Tenggara and the Moluccan samples, slightly shifted towards the outlying “eastern” populations from NG and the Admiralties. Little gene flow was also indicated with the outlying populations from Polynesian and the Solomon Islands; this mirrored the distant position shown by the genetic indices. Closer to the main cluster, about equidistant to East Timor, were the ISEA outliers Nias, Java, Mentawai and, interestingly, also the Australian sample in the small range analyzed. All outlier islands were characterized by low haplotype diversity/high RMP (see above).

**Table 3:** **Genetic diversity of the East Timor population and 24 surrounding populations using HVS-I**

**Population**	**Reference**	**n** ^**a**^	**hts** ^**b**^	**Unique hts**	**MPD** ^**c**^	**RMP** ^**d**^
1 - **East Timor (pooled)**	[3], this study	362	94	52 (14%)	5.976 ± 2.857	0.039
1A - **East Timor**	this study	324	87	49 (15%)	5.938 ± 2.841	0.039
2 - **Nusa Tenggara**	[3,5,24,65]	1699	345	178 (10%)	5.816 ± 2.783	0.017
3 - **Bali**	[5,24]	570	138	71 (12%)	5.643 ± 2.711	0.034
4 - **Java**	[5,24]	97	49	31 (32%)	5.570 ± 2.698	0.035
5 - **Sumatra**	[5,17,40]	228	107	70 (31%)	5.520 ± 2.664	0.023
6 - **Mentawai**	[5]	128	16	4 (3%)	4.687 ± 2.311	0.126
7 - **Nias**	[5,41]	499	61	29 (6%)	4.766 ± 2.334	0.153
8 - Peninsular Malaysia*	[17,57]	470	158	98 (21%)	6.350 ± 3.016	0.029
9 - **Borneo**	[24]	157	102	77 (49%)	5.345 ± 2.593	0.017
10 - **Sulawesi**	[5,24]	437	153	108 (25%)	4.804 ± 2.351	0.039
11 - **Moluccas**	[24,65]	74	36	23 (31%)	5.425 ± 2.643	0.055
12 - **Philippines**	[24,28]	483	140	89 (18%)	5.052 ± 2.457	0.035
13 - **Vietnam**	[58]	187	119	91 (49%)	5.445 ± 2.634	0.018
14 - **Laos**	[59]	214	127	95 (44%)	5.689 ± 2.737	0.018
15 - **Thailand**	[60]	190	108	79 (42%)	5.584 ± 2.694	0.02
16 - **South Korea**	[61]	692	306	215 (31%)	4.853 ± 2.370	0.019
17 - **Taiwan (indigenous)**	[24,45]	718	86	35 (5%)	5.197 ± 2.518	0.042
18 - **Hainan**	[62]	293	99	45 (15%)	5.338 ± 2.583	0.025
19 - **Mixed Han (China)**	[63]	262	194	159 (61%)	5.533 ± 2.668	0.008
20 - **West New Guinea**	[66]	227	74	39 (17%)	7.045 ± 3.320	0.046
21 - **Papua New Guinea**	[34,64]	201	72	34 (17%)	6.733 ± 3.188	0.046
22 - **Admiralty Islands**	[67,68]	203	41	20 (10%)	5.693 ± 2.739	0.152
23 - **Solomon Islands**	[29]	703	102	41 (6%)	3.660 ± 1.855	0.208
24 - **Polynesia**	[65,67]	394	56	28 (7%)	1.793 ± 1.038	0.340
25 - **Australia (indigenous)**	[34,69]	146	50	22 (15%)	4.555 ± 2.252	0.042

*including Singapore.

^a^number of mtDNAs.

^b^number of haplotypes.

^c^mean number of pairwise differences.

^d^random match probability.

Reading frame: nps 16080–16180, 16195–16354; total sample size: n = 9,634. For geographic locations, see Figure 1.

### Elucidating the initial settlement from its maternal footprints

**Figure 5:**
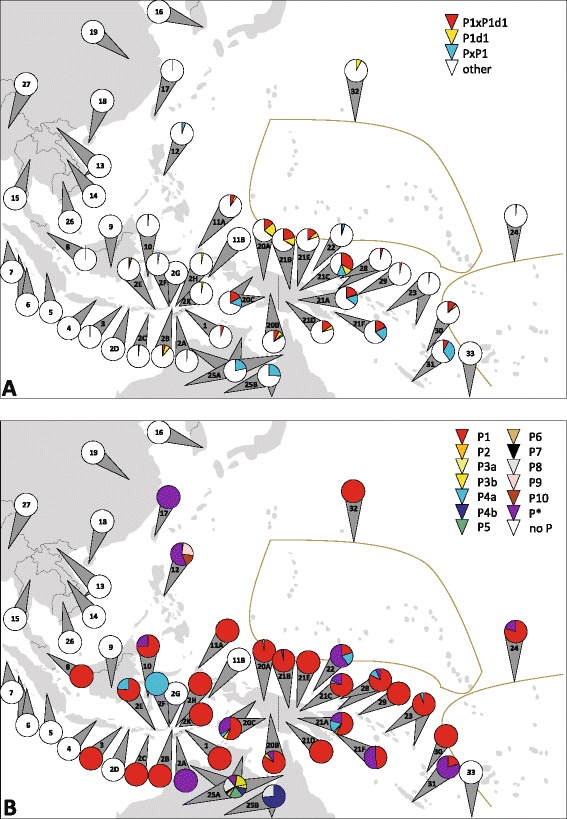
**Distribution of haplogroup P mtDNAs in East Timor and surrounding populations. (A)** P1 and P1d1. These categories discernible with HVS-I data enabled equal treatment of all populations included. All other, including unassigned, P clades are combined into “PxP1”; **(B)** P1-P10. All available information was considered (reading frames varied). The total of haplogroup P has been set to 100% for every population to better depict (small) proportions. Inconclusive P samples were denoted as “P*”. Frequencies are indicated by colored circle segments. See legends for color codes. Population reference numbers located within a circle indicate that the analyzed haplogroup(s) were not found in this population. Populations: 1 - East Timor, 2 - Nusa Tenggara (2A - West Timor, 2B - Lembata, 2C - Sumba, 2D - Lombok, 2E - Flores, 2F - Solor, 2G - Adonara, 2H - Pantar, 2K - Alor), 3 - Bali, 4 - Java, 5 - Sumatra, 6 - Mentawai, 7 - Nias, 8 - Peninsular Malaysia, 9 - Borneo, 10 - Sulawesi, 11 - Moluccas (11A - Ternate, 11B – Ambon), 12 - Philippines, 13 - Vietnam, 14 - Laos, 15 - Thailand, 16 - South Korea, 17 - Taiwan, 18 - Hainan, 19 - Mixed Han (China), 20 - WNG (20A - highlands, 20B - lowlands, 20C - Southwest and Lowland Riverine), 21 – PNG (21A - pooled, 21B - Wewak, 21C - Bundi, 21D - Gidra, 21E - East Sepik, 21F- Kula Ring), 22 - Admiralty Islands, 23 - Solomon Islands, 24 - Polynesia/Fiji, 25 - Australia (25A - Northwest, Northern Territory, Far North Queensland, 25B - New South Wales, Paakintji, Ngiyambaa), 26 - Cambodia, 27 - Myanmar, 28 - New Britain and New Ireland, 29 - Bougainville, 30 - Vanuatu, 31 - New Caledonia, 32 - Micronesia, 33 - New Zealand Maori. For more information, see Additional file 11.

Haplogroup P exhibited widespread distribution in both northern and southern Sahul (and beyond) as the only autochthonous lineage. In our efforts to reconstruct the initial migrations between Sunda and Sahul we therefore particularly focussed on this haplogroup. P generally occurred at frequencies <10%, but central and southern populations revealed proportions up to 56.3%. In most cases (also in East Timor), these mtDNAs completely or mainly consisted of P1 representatives; the highest P1 proportion was reached in PNG (43.8%). P1 lineages not further resolved by HVS-I (P1*) and the derived P1d1 mostly occurred together. The remaining P lineages were less widespread even if combined and always occurred in parallel with and in smaller proportions than P1 (when more than a P singleton was present), except in Australia (where P1 was virtually absent, see below) and the Philippines, as well as New Caledonia, the Admiralty Islands and the Kula Ring (where more unassigned P than P1 samples were found). PxP1 proportions >3.9% were only revealed in the Philippines, NG, Australia, and New Caledonia (up to 32.0%) (Figure 5, Additional file 11).

Both an eastern Indonesian and a Melanesian (Near Oceanian) origin for haplogroup P(1) have been proposed [3,28,48,70]. The high-resolution phylogeographic reconstruction using the 20 available complete mitogenomes already indicates that not even the basal P1 diversity is concentrated in eastern Indonesia (Figure 3): all sublineages were also found in NG, and samples from that island and the Philippines pointed out additional branches at all hierarchical levels [52-55]. A western Melanesian cradle is also supported by published CR data. Various P1 sublineages are dispersed in eastern Indonesia and beyond, but both the frequency and diversity peak lay in NG, whose central role was confirmed by the remaining P clades. Besides the more widespread P4a, NG shared P3 with Australian populations, and P9 only found in Taiwan and the Philippines (the latter based on a CR pattern, maintained until Phylotree’s build 15 [49]). The only P lineages not found in NG were P10 from the Philippines, and those restricted to Australia, viz. P4b and P5-8. *Vice versa*, P2 is only reported in NG, but has not been assessed in most other populations (Figure 5, Additional file 11).

The coalescent ages of the autochthonous Southwest Pacific haplogroup P fit intriguingly well with the archaeological dating of the first settlements in this area (~48-40 kya): it was estimated to 51.7-65.4 ky based on CR [5,34,64,71] and 54.8 ky using complete mitogenomes. The latter calculations yielded 41.3-53.0 ky for P2’10, P3, P4 and P6; estimates for the remaining clades were not made due to paucity of data [72]. Our maximum likelihood (ML) point estimate for haplogroup P1 considering the entire mtDNA molecule and all 20 sequences was 36.1 ky, P1d was dated at 31.9 ky, P1d1 at 28.1 ky and P1e at 27.4 ky. Rho (ρ) statistics yielded remarkably similar estimates completely overlapping with the ML ranges (Table 4). Thus, there is no indication for an age underestimation by ρ statistics, an effect noticed using low-resolution data [73]. The novel dating of P1 was consistent with results using fewer samples [72]. Partial mitogenome-based age reports for P1 were 28.0-53.8 ky [3,5,34,44,48,71]. It appears that haplogroup P and its sublineages arose in a successful period at the cusp of or shortly after the first arrival of anatomically modern humans, merely the most successful clade P1 occurred separately and later.

**Table 4:** **Molecular divergences and age estimates obtained by Maximum Likelihood and Rho statistics for haplogroup P1**

**Haplogroup**	**N** ^**a**^	**All nucleotide substitutions**							
		**ML** ^**b**^	**S.E.** ^**c**^	**T (ky)** ^**d**^	**ΔT (ky)** ^**d**^	**ρ**	**σ**	**T (ky)** ^**d**^	**ΔT (ky)** ^**d**^
**P1**	20	12.8	1.2	36.1	3.7	12.5	1.4	35.3	4.3
**> P1d**	11	11.4	1.0	31.9	3.1	11.1	1.3	31.1	3.9
**>> P1d1**	4	10.1	1.1	28.1	3.2	9.5	1.9	26.3	5.6
**> P1e**	6	9.9	1.6	27.4	4.8	9.7	2.7	26.9	8.1

^a^number of complete mtDNA sequences.

^b^maximum likelihood molecular divergence.

^c^standard error.

^d^age estimates using the corrected molecular clock [74].

The P lineage spectrum in Australia is distinct from those from all other areas (as is possibly that of the Philippines and Taiwan). It is made up almost completely of the most ancient clades (P4b, P5-8; 48.0-52.9 ky); P3, rare and shared with NG at a very low level (≤1.7%), is also rather old (41.2 ky). Despite a singular report of the only other shared P lineage, the youngest and elsewhere predominant P1, this contradicts an origin of haplogroup P in central/southern Sahul (Australia) followed by (back-)migrations to northern Sahul (NG). Rather it favours a southward colonization prior to ~36 kya, when P1 developed, and little later north–south exchange even though a landbridge existed until ~8 kya [25,34,44,53,69,75-77]. Developing this scenario further, the more recent P clades, such as P1, would have arisen later during an “incubation” stage in northern Sahul from root P* mtDNA carriers left behind. Therefore, they were not or very limitedly represented on the forefront of settlers that proceeded southward and gave rise to Australian mtDNA diversity (*cf*. [78] for a similar scenario for South America). Our age estimates also contraindicate that haplogroup P1 is old but simply did not move on [77]. Intriguingly, the singular reported Australian P1 haplotype was revealed in a Northeast Aboriginal population considered closer to SEA Natives than other Australian groups in oral history and due to anthropological traits [77] (*cf.* the “negrito” ancestral connection hypothesis [31]). Apart from that, it could also derive from more recent, possibly individual, migration or displacement – a general caveat when equating lineage ages with timing of migration.

**Figure 6:**
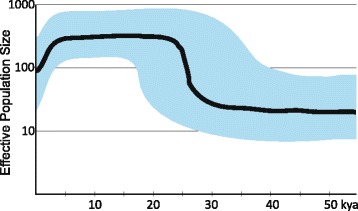
**Bayesian Skyline Plot of mtDNA haplogroup P1.** The hypothetical effective female population size is based on the complete P1 mitogenomes (Additional files 4 and 5) assuming a generation time of 25 years.

The synopsis of haplogroup P data indicates that eastern Indonesia, including East Timor, was not the cradle of this founder lineage, thus probably did not lie on the initial main route that likely led into northern Sahul, but was rather populated from there after haplogroup P1 (36.1 kya), P1d1 (28.1 kya), and likely even P1e (27.4 kya) (whose dispersal data are scant), had arisen. The landmass increase from ~30 kya (see above) could have triggered the demographic expansion at ~28-24 kya indicated in the Bayesian Skyline Plot (BSP) from western Melanesian P1 sequences (Figure 6), the time when all known P1 subclades emerged (29.7 ± 2.3 kya) (Table 4) - and could also have led to a geographic expansion that brought, among others, carriers of haplogroup P1 westward. The lack of PxP1 lineages in East Timor may be caused by the fact that a finite sample does not cover each and every rare lineage, or by drift, as they are found in and around eastern Indonesia [3,5,55]. For the same reasons - and the lack of mitogenomic data - we would not consider a more detailed dating of the westward movement reliable as it is based on the presence of only a P1e sublineage and the lack of P1d1 in East Timor as *termini ante* resp. *post quem*. The second most widespread P lineage, P4a, is much younger (18.6 ky) [72], absent from Australia, could also have arisen in northern Sahul, thus supporting an ongoing dynamic phase (Figure 5, Additional file 11).

**Figure 7:**
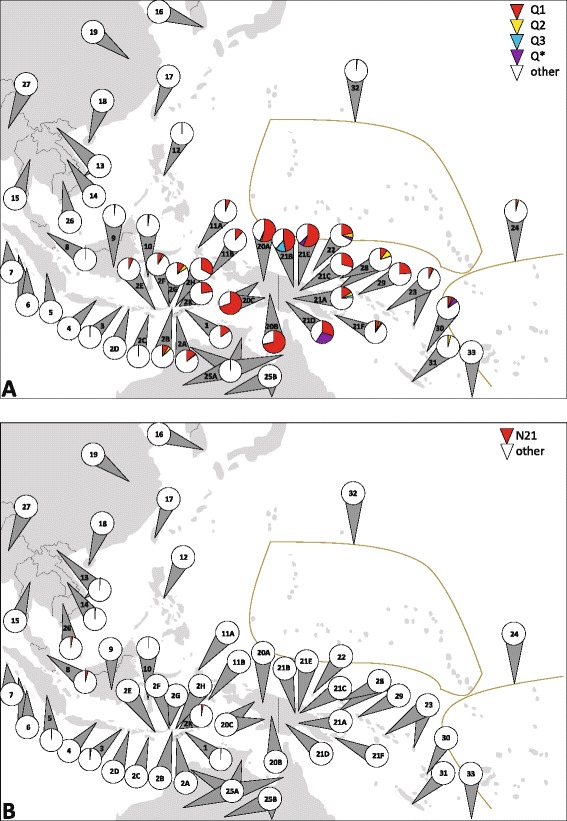
**Distribution of mtDNA haplogroups Q and N21 in East Timor and surrounding populations. (A)** Haplogroups Q1-Q3. Unassigned/inconclusive Q samples were denoted as “Q*”; **(B)** Haplogroup N21. All available information was considered (reading frames varied). Frequencies in populations are indicated by colored circle segments. See legends for color codes. Population reference numbers located within a circle indicate that the analyzed haplogroup(s) were not found in this population. For the populations included, see Figure 5. For details and references, see Additional file 11.

The distribution of haplogroups Q and Q1 resembled that of P and P1 to a very high degree. Q carriers were only absent in the southern- and northernmost populations included; the possible cradle of this indigenous haplogroup, again lay in NG: proportions reached >70%, and only there and in the Bismarck Archipelago, all three Q clades were found. Proportions >10% were revealed in the Moluccas (11.6%), East Timor (15.5%) and its northwest Nusa Tenggara neighbor islands (≤32.8%), as well as in western Melanesia (NG and its Northeast, ≤71.7%). Haplogroup Q proportions consisted predominantly of Q1, the most frequent “pioneer” lineage of eastern Indonesia in general [3] and East Timor, highly diverse even in the CR (Additional file 3). Q2 is widespread, and generally rare (except in the Bismarck Archipelago). Lineage Q3 was found on the latter, in this study (<1%), and in NG (≤14.9%), however, only one subclade carries HVS-I polymorphisms. A single Q sample was reported from Australia, a Q2 – again from the North [34] (Figure 7, Additional file 11). Haplogroup Q has been dated to 32.0-44.5 (even 74.6) ky using partial [5,34,44,64] and 37.5 ky using complete mitogenomes [72]. The age estimates for Q1, Q2, and Q3 from the latter study were 18.1, 28.7 and 31.0 ky, respectively, and widely ranged (2.9-48.0 ky) when partial mitogenomic data were employed [3,5,34,44,48,64,71].

The complete absence of haplogroup Q carriers from Australia (except a single occurrence; possible explanations are discussed above) indicates that southern Sahul was populated before this lineage arose (37.5 kya), as we postulate for haplogroup P1. The outlined westward movements might also have caused the wide spread of the predominant Q1, the youngest Q subclade, after 18.1 kya - the timing we hypothesised from haplogroup P4a (18.6 kya) (Figure 7, Additional file 11). These coincidences support a common history of haplogroups P1, P4a and Q(1) and their spread probably even within the same group of settlers - separated from the events that led to the colonization of today’s Australia.

**Figure 8:**
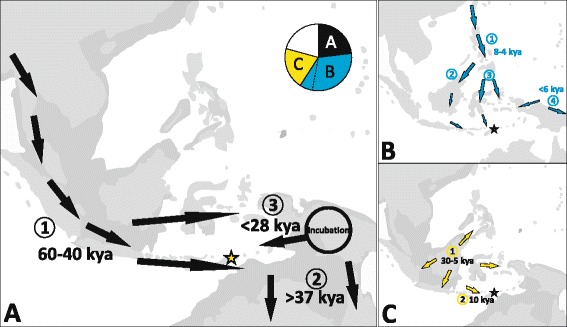
**Migration routes and their contribution to the East Timor mtDNA pool.** The major postulated migration events into ISEA and our novel findings are depicted. The asterisk highlights East Timor. The darker grey areas indicate the predicted late Pleistocene coastline. **(A)** The initial human settlement carrying haplogroups P, Q, N21 and others that arrived (1) between 60–40 kya. Our results indicate (2) a colonization of Australia (southern Sahul) before 37 kya and (3) an incubation period in northern Sahul (NG) followed by westward expansions after 28 kya; **(B)** The Holocene (1) southward out of Taiwan movement marked by haplogroups M7c1, D5, F1a3, F1a4 between 8–4 kya followed a (2) western or (3) eastern route (that we favour for East Timor), and (4) a local arisal, possibly connected to (1), of the “Polynesian motif” ~6 kya followed by west- and eastward migrations; **(C)** the postglacial expansion of haplogroup E (and others) (1) originating in eastern Sunda and a dispersal 30–5 kya that (2) reached eastern Indonesia ~10 kya. The inlay pie chart in **(A)** depicts the proportions of haplogroups associated with **(A)**, **(B)** and **(C)** within the extant East Timor population. The dashed line separates the proportion of the “Polynesian motif”, as it has also been described to derive from a separate event. See text for details.

Westward movements after a stall in migration in northern Sahul are the most likely scenario that explains our findings (Figure 8). They have also been postulated in archaeology from old Australian *vs*. younger Timorese sites. Our genetic dating is however much younger than the first known settlements in Timor (>42 kya), but older than those of modern humans in Flores (<12 kya) [27]. A northward migration after an initial colonization of the South (>37.5 kya) appears less likely because of the complete lack of old Australian P lineages everywhere else. Several mtDNA genetic indices confirm that the relation between East Timor and NG is indeed not a recent one (see above). NG populations, due to their extremely high frequencies of haplogroups P and Q, appear generally far from all ISEA populations in these analyses. Still, East Timor yielded the least genetic differentiation from NG of all samples included, possibly because of its most proximal location in westward expansions (Figures 5 and 7, Additional files 8, 10 and 11). The “*almost complete (female) isolation between the two regions*” [48] is also seen in nuclear DNA investigations. These support a continuous population history for Sahul dating to probably ≥50 kya demonstrating a deep common origin of Australians and Papua New Guineans with little later migration [36,79]. The often depicted split of initial Sahul settlers into a northern and southern group after arrival on the continent, followed by isolation (*e.g.*, [25,34,48]), would not explain the age gap in mtDNA lineages between South and North alone, unless an “incubatory” phase is taken into consideration. Separate settlement waves from Sunda to NG and to Australia [3] would also need to integrate the later development of P1 and Q from root haplotypes.

From the similarly small proportions of autochthonous haplogroups along both, we cannot favour a northern or southern route through Wallacea for the initial settlers [20,25,27]. The clustering of the Sulawesi and MSEA populations in the MDS plot rather derives from the footprints of later inputs in the mtDNA pool (Figure 4, Additional file 11).

Haplogroup N21, another postulated “first settlement” marker, found in proportions ≤4.2% in MSEA and ISEA, has been dated to 22.4 ky [72]. East Timor was its easternmost location. This evidence is not easily compatible with migration scenarios (Figure 7, Additional file 11). In total, the autochthonous lineages considered to have arisen in the course of the initial dispersal, *i.e.* P, Q, M21, M73, N21, R14, and possibly further unresolved M* and R* clades [3,5,17,24,28,34,40,55,80], account for 22.2% of our East Timor sample. This confirms previous estimates for ISEA [24].

### Traces of later settlements

We do not find that “*the mitochondrial genomes of Timorese women predominantly derive from Papuan progenitors*” [4], but rather that additional migration after initial colonization is necessary to explain the predominant proportion of (S)EA lineages, mirrored by high haplotype sharing rates and relatively small genetic, despite large geographic, distance (Figure 2, Additional files 7, 8, 9 and 10). We investigated East Timor’s mtDNA composition (as a pooled sample from this study and [3]) in the light of four particular “later” migratory events postulated to have occurred between (S)EA and Melanesia during late Pleistocene and mid-Holocene in previous studies, often overlapping in terms of time and geography. When their contributions are merged, 52.3% of the East Timor population (53.3% of our sample) can be explained. Figure 8 depicts the migration routes we found to be relevant to the extant East Timorese mtDNA pool.

(i) A *Neolithic OOT expansion into ISEA in mid-Holocene ~8-4 kya* [3,5,24]: its postulated mtDNA marker, the former haplogroup entity M7c3c (see Additional file 12), reached a frequency of 12.4% in East Timor (13.9% in our sample set). Also haplogroups D5 (1.4%), F1a3 (4.4%), F1a4 (8.6%), and F3b(1) (0.3%) have been associated with this migration [24,28,30,80]. Thus, 27.1% (our sample: 29.6%) of the East Timor mtDNAs could be related to this dispersal. A ~20% contribution for ISEA was previously estimated [24]*.* The OOT-related mtDNA haplogroups (as of published records) peak in the Philippines and eastern ISEA, but are almost absent from MSEA (except the basal D5), Melanesia, Polynesia, and Australia (Additional file 12): this migration might thus have ended in eastern Indonesia and not proceeded eastward as hypothesized [28] (see below). A western (Taiwan-Philippines-Borneo-Sumatra/Java) and an eastern (Taiwan-Philippines-Sulawesi/Moluccas) route into ISEA have been suggested [3,5,24,30,81], as well as several subevents via more than a single route [28]. Our results support an eastern route into East Timor: the combined marker haplogroups’ proportions were larger along this route (Additional files 11 and 12), the western route marker Y2 [24] was absent in East Timor, and the haplotype-based genetic parameters put East Timor closest to Nusa Tenggara (probably relevant in both routes), and Sulawesi and the Moluccas, proximal only on the eastern route. In the MDS plot, Sulawesi located at some distance from the cluster of East Timor-Nusa Tenggara-Moluccas, but closest to the Philippines and Taiwan, the postulated origin of the migration. Larger distances from the latter two, but similar ones to East Timor, were yielded by the “western route” populations (Figure 4, Additional files 7, 8, 9 and 10).
(ii) A *pre-mid-Holocene expansion in(to) ISEA and Polynesia* [16,65,70]: marker haplogroup B4a1a1 probably originated in the vicinity of the Bismarck archipelago and might have reached Timor westward along a “voyaging corridor” [16,37]: the wide spread of this lineage (and AN languages) [56,82,83], highlights early navigating routes and capabilities. The so-called “Polynesian motif” (B4a1a1a until Phylotree [49], build 15) comprises 5.0% of the East Timor sample (additional 5.6% for B4a1*) (Table 1). Notably, B4a1a has also been considered an OOT marker [28,30]; hence, the “maternal” contribution of that event to East Timor would rise to 32.1% (34.5% in our sample). The two dispersals would be strongly interwoven, and eastern ISEA (East Timor) would not only represent the last section of a southward corridor (see above), but also the assumed bottleneck through which only few haplogroups made it to Melanesia and Polynesia [28,84,85].
(iii) A *major postglacial eastward expansion into ISEA in late Pleisto- and early Holocene ~30-5 kya* ([15,24,30]: “early train”): accordingly, lineage E originated on the eastern Sunda coastline and during this expansion, caused by the rising sea, reached eastern Indonesia ~10 kya [15,16,24]. Carriers were widespread in ISEA and Near Oceania, with a focus off the former eastern rim of Sunda. The second highest frequency was observed in East Timor (19.3%). The second marker lineage, B4c2, was generally rare. Altogether, 19.9% of the East Timor dataset (18.5% of our sample) could be traced back to this Neolithic dispersal (Additional files 11 and 12). Remarkably, also haplogroup E has been linked to the OOT expansion [42].
(iv) A *Holocene arrival ~7 kya from MSEA to ISEA* [24,40] may have played a minor role in populating its far East. Marker haplogroup F1a1*, frequent throughout eastern Indonesia, MSEA, and on Taiwan, is a singleton in the Philippines (*contra* OOT) and East Timor (0.3%), where the second marker haplogroup, N9a6, was absent (Additional files 11 and 12)*.*

## Conclusions

Our results from the oldest indigenous mtDNA lineages of the Southwest Pacific region indicate migratory events that commenced <28 (possibly <18) kya, more than 15–20 ky after the initial arrival to Sahul. We thus confer the extension of the rather strictly timed classical two stages (*cf*. [44]) also upon the “initial wave” of colonization and suggest a much broader timescale of events connected to indigenous haplogroups. Secondary expansions of initial settlers and westward migrations have been postulated before (*cf*. [27]) and dated at ~40 kya [48]. This is the first clear genetic indication of a temporal proximity and possible coincidence of dispersals which encompassed autochthonous haplogroups with postulated “later” events of (S)EA origin reconstructed from younger lineages. In our scenario, it seems now probable that the “initial” and “later” migration routes, or even the population groups themselves, were or became intertwined. Ongoing genetic (and linguistic) “diffusion” of humans (that at some point also brought P and Q carriers to islands east of NG) was likely facilitated once trade corridors were established [16,37,80,86]. The peak of Sahul’s surface area ~30-18 kya coincides with the highly dynamic migratory period we report and might have triggered it. Population dispersals (geographic expansions) do not necessarily also mean population expansion [4], but BSPs from thousands of samples in the HVS-I range (Indonesia) and tens of complete mitogenomes (Philippines and Malaysia) from mixed haplogroups have (remarkably similarly to our BSP from haplogroup P1; Figure 6) indicated a slowly growing population in the Pleistocene that peaked at 20–15 kya and declined thereafter [4,30,87]. This seems to pinpoint “*substantial impact of common environmental forces*” in ISEA [4].

In 1869, AR Wallace noted that the Timorese appeared closer to inhabitants of the Moluccas and NG than SEA and speculated about admixture [88]. Almost one and a half century later, we tried to shed light on these relations by characterizing the mtDNA composition of East Timorese. The blurring of older “footprints” by subsequent migrations, and the paucity of available (founder lineage) mitogenomic data may have confounded our assumptions on dispersal and time estimates. In any event, the migration history (to be) reconstructed from mtDNA is only the “female side” of the medal, but “*necessarily coupled*” to the history of the entire population [4]. Other genetic markers (*cf*. [36,86,89]) will support or modify our proposals, but contribute to the complete picture only together with non-genetic disciplines, such as archaeology (*cf*. [90]) and linguistics - particularly, since the geographically overlapping AN and Papuan language families are thought to be separable in time; with the reservation of difficulties in speaker affiliation and language grouping (*cf*. [3,29,59]). All-embracing studies on human colonization will need to consider also other hominin species or populations [20,91-93].

## Methods

### Sample collection

Three hundred and twenty-four randomly selected East Timor residents (175 female, 149 male) voluntarily donated buccal swabs under informed consent. The study was approved by the Universidade Nacional Timor Lorosa'e. For most individuals, maternal origin within East Timor could be verified over three generations; for 36 donors, information was incomplete but indicated East Timor origin. Their birthplaces comprised all 13 districts. One donor was born in Flores (Indonesia) with a maternal line traceable to East Timor. Five donors indicated maternal origin in West Timor, one in Java (both Indonesia), four could not provide any information. All were included in this study to reflect the extant population (Additional files 1 and 2).

### MtDNA sequence data generation and interpretation

DNA was extracted using standard protocols [94]. The forensic quality CR (nps 16024–16569, 1–576) Sanger type-sequencing (STS) protocols included amplification of a single segment, redundant sequencing coverage, independent data inspection and final validation to ensure precise base calling [95]. Contiguous sequences were aligned [96] with respect to the rCRS [50] using Sequencher v5.0 (GeneCodes Corporation, Ann Arbor, MI, USA) and assigned to mtDNA haplogroups according to Phylotree [49], build 16, aided by the EMMA software package [97]. In order to define their phylogenetic position, five mtDNAs were subjected to entire mitogenome STS following forensic protocols [98]. To resolve the still poorly described phylogeny of haplogroup P1, also three P1 samples were included in these analyses.

Emerging MPS solutions now offer a more accessible option to yield highly desired full mitogenome data [99], but investigations benefiting from phylogenetic knowledge found published data inappropriate for the quality needs of the field requiring highest accuracy [100] - just as STS flaws attracted attention in the last decade (*e.g*., [101]). Few studies applied MPS on mtDNA in a forensic environment [102-106]. In this study, we used the Ion Torrent PGM under forensic quality control for the first time on a (small) population sample by stand-alone approach for complete mitogenome MPS on the eight samples described above and nine additional *bona fide* P1 samples. We used Ion PGM Sequencing 200 Kit v.2 chemistry on an Ion 316 chip, and applied strict quality control according to a preceding validation study of the PGM in forensic mtDNA sequencing, that suggested MPS, with appropriate care, as a valuable alternative also in a forensic environment [106]. Raw data were inspected twice using independent software, mirroring the gold standard in STS (see above). The latter was applied *ex post* to clarify remaining discordance. Phylogenetic plausibility checks were performed on the dataset.

### Phylogenetic reconstructions, age and demographic estimates

The most parsimonious phylogenetic trees were manually reconstructed from the 20 haplogroup P1 sequences, as well as the five non-P1 complete mitogenomes to depict their position among published sequences [17,28,44,52-55,80,87,107-116]. To obtain ML molecular divergences for haplogroup P1 PAML 4.4 [117] was used, assuming the HKY85 mutation model with γ-distributed rates, as previously suggested [118]. The ML estimates were compared with those obtained from the averaged distance (ρ) of a clade’s haplotypes to the respective root haplotype, accompanied by a heuristic estimate of the standard error (σ) calculated from an estimate of the genealogy. All calculations were performed on the complete mtDNA haplotypes. Mutational distances were converted into years using a corrected molecular clock [74]. Concerns about the estimation accuracy via ρ statistics have been raised considering the small and hypervariable CR [73]; recent studies based on entire mitogenomes found good concordance between ML and ρ results (*e.g*., [38,119]).

The P1 complete mitogenome dataset was analyzed with BEAST v.1.7 [120] to obtain a BSP of the effective female population size. We used the HKY substitution model (γ-distributed rates) and a relaxed molecular clock (lognormal in distribution across, and uncorrelated between branches) for 5,000,000 iterations, drew samples every 10,000 Markov chain Monte Carlo steps, and visualized the output using Tracer v.1.5 [120] assuming a generation time of 25 years, as in [4,119].

### Analysis of maternal relatedness

To avoid a biased representation of lineages by closely related individuals, donors with identical mitogenomes were inspected for maternal relatedness. Also those that only varied in polycytosine- and (AC)_n_-stretch lengths were considered, as such differences can result from analysis and detection conditions [100,121-123]. After typing 15 autosomal STR loci and the amelogenin length polymorphism, pedigree construction, and likelihood ratio (LR) calculation using reported STR allele frequencies [7] [correcting the 10.2 allele frequency of D18S51 to 0.0 (L Souto, *pers. comm*.)], no donor pair revealed close maternal relatedness (*i.e.*, mother-child and sibling constellations) applying a cut-off LR of 1,000 [124,125] (data not shown).

### Forensic and population genetic parameters of East Timor and surrounding populations

The mtDNA composition of East Timor was compared to populations from surrounding areas by calculating haplotype-based forensic and population genetic intra- and interpopulation parameters, taking the entire quality-checked datasets into account. Samples of identical geographic origin were merged from different publications to better reflect true variation and reduce any effects of error in small samples (*cf*. [24]) after careful inspection of metadata to avoid multiple representations of individuals. We determined the proportions of unique haplotypes, RMPs (as sum of squared mtDNA haplotype frequencies), counted MPD within populations and between populations (both uncorrected and corrected, *i.e.* reduced by the mean of MPD observed within the two populations compared), performed an analysis of molecular variance (AMOVA) using ARLEQUIN v.3.5.1.2 [126] and generated an *F*_ST_ distance matrix for Multidimensional Scaling (MDS) analysis with the R software package [127] function cmdscale(). All sequences were trimmed to a greatest common range: (*i*) eleven populations (3,276 samples) from (S)EA and Melanesia [28,34,45,57-64] were included based on HVS-I and -II data (nps 16080–16193, 16194–16365, 73–300); (ii) another analysis covered 25 populations (9,634 individuals) from (S)EA, Melanesia, Polynesia, and Australia [3,5,17,24,28,29,34,40,41,45,57-69] in an HVS-I range (nps 16080–16180, 16195–16354). See Figure 1, Tables 2 and 3 for details.

### Phylogeographic investigations

To assess the distribution of selected marker mtDNA haplogroups, frequency values in surrounding populations were collected from published records after phylogenetic inspection [3,5,17,24,28-30,34,40,41,44,45,48,52,53,55,57-69,75-77,80,86,115,116,128-141]. Data were mostly restricted and sample sizes often very small. As outlined, they were merged in case of identical geographic origin. To analyze the dispersal of haplogroup P(1) in greater detail, we distinguished categories discernible in HVS-I: P1*, P1d1, and the “non-P1” P samples (PxP1). We chose the greatest common (*i.e.* available, in many cases) sequenced fragment for an unbiased picture and assumed haplogroup P1 status when a minimal pattern of [73G 263G 315.1C] 16357C, relative to the rCRS [50], was present, as done before [3], because the remaining current CR markers ([49], build 16) were not found reliable (see above). The transition at np 16357 is a phylogenetic marker of 17 additional mtDNA haplogroups ([49], build 16). All are clearly discernible from P1 by their HVS-I pattern and/or are not expected to occur in the investigated area. Supporting our assumptions, all haplotypes in EMPOP [51] (v2.3, release 11, n = 34,617) that carried a private transition at np 16357 and could be mistaken for P1 fell into the latter category. In further analyses, we looked at the dispersal of the remaining P and other marker lineages, inasmuch they were detectable from available data. We labelled P and Q mtDNAs not assigned to a specific lineage due to lack of information as “P*” and “Q*”.

To assess and depict the impact from surrounding regions on the mtDNA pool, the East Timor haplogroups were classified according to their geographic origin as per previous reports, where terminology and assignments were overlapping and contradictory at times [3,5,15,16,24,28-30,32,34,40,70,80].

### Availability of supporting data

The sequence data generated are available in GenBank [KJ655583-KJ655889, KJ676774-KJ676790; http://www.ncbi.nlm.nih.gov/genbank] and included in EMPOP [EMP00534; http://empop.org].

## Additional files

Additional file 1:
**Origin of the 324 East Timor sample donors. East Timor and its 13 districts are shown.** The diameter of every circle is proportional to the number of samples deriving from each district (see legend). The asterisk indicates the capital, Díli. The Díli district includes Ataúro island. The striped area is part of Indonesia. For a detailed list, see Additional file 2. Additional file 2:
**Detailed origin of the 324 East Timor sample donors.** The district of origin is listed for each donor. n.a. = not available. Additional file 3:
**CR profiles, haplogroups and GenBank accession numbers for the 324 East Timor samples.** Haplogroups according to [49], build 16. Differences are relative to the rCRS [50]. Bases are indicated according to the IUBMB nucleotide code. The prefix @ indicates the reversion of a mutation occurring earlier in the phylogeny. Differences indicating potentially novel, not (yet) defined haplogroups are indicated as suffixes. Additional file 4:
**Mutational profiles, haplogroups and GenBank accession codes of the 17 novel complete East Timor mitogenomes.** Haplogroups according to [49], build 16. Differences are relative to the rCRS [50]. Bases are indicated according to the IUBMB nucleotide code. Additional file 5:
**List of the complete haplogroup P1 mitogenomes depicted in Figure** **3** **.**
Additional file 6:
**Novel non-P1 complete mitogenomes from East Timor.** The five East Timor samples are shown in the context of published sequences. Color codes indicate the country of origin. All identified differences are relative to the rCRS [50], disregarding cytosine insertions after nps 16193 and 309. Haplogroups according to [49], build 16. Bases are indicated according to the IUBMB nucleotide code. The prefix @ indicates the reversion of a mutation occurring earlier in the phylogeny. Underlined mutations are recurrent in the tree. References and GenBank accession numbers are indicated for each sample. Samples marked with an asterisk are available from GenBank only [Pradutkanchana 2010, unpublished data]. Additional file 7:
**Shared haplotypes between East Timor and 10 surrounding populations based on HVS-I and HVS-II.**
Additional file 8:
**Intra-population comparisons for East Timor and 10 surrounding populations based on HVS-I and HVS-II.** Part A: Design and results of AMOVA. Part B: Mean pairwise differences. Part C: *F*_ST_ comparisons. Additional file 9:
**Shared haplotypes between East Timor and 24 surrounding populations based on HVS-I.**
Additional file 10:
**Intra-population comparisons for East Timor and 24 surrounding populations based on HVS-I.** Part A: Design and results of AMOVA. Part B: Mean pairwise differences. Part C: *F*_ST_ comparisons. Additional file 11:
**Frequency of selected mtDNA haplogroups in East Timor and surrounding populations.** For a graphical representation of frequencies, see Figures 5 and 7 and Additional file 12. Additional file 12:
**Dispersal of selected mtDNA haplogroups in East Timor and surrounding populations.** (A) Haplogroups related to the “out of Taiwan” dispersal. (B) Haplogroups B4c2 and E; (C) Haplogroup F1a1. All available information was considered (reading frames varied between publications). Frequencies in populations are indicated by colored circle segments. See legends for color codes. Population reference numbers located within a circle indicate that the analyzed haplogroup(s) were not found in this population. For the populations included, see Figure 5. For details and references, see Additional file 11.

## Abbreviations

AMOVA: Analysis of molecular variance
AN: Austronesian
BSP: Bayesian Skyline Plot
codR: Coding region of the mtDNA
CR: Control region of the mtDNA
EA: East Asia(n)
EMPOP: European DNA Profiling Group MtDNA Population Database
HVS: Hypervariable segment of the mtDNA
ISEA: Island SEA
ky: Thousand years
kya: ky ago
MDS: Multidimensional scaling
MPD: Mean pairwise differences
MPS: Massively parallel sequencing
MRCA: Most recent common ancestor
MSEA: Mainland SEA
mtDNA: Mitochondrial DNA
NG: New Guinea(n)
np: Nucleotide pair
OOT: “out of Taiwan” (dispersal)
PNG: Papua NG
rCRS: Revised Cambridge Reference Sequence
RMP: Random match probability
(S)EA: (South) EA
STR: Short tandem repeat
STS: Sanger-type sequencing
WNG: West NG
