# Supplementary material for: Human settlement history between Sunda and Sahul: a focus on East Timor (Timor-Leste) and the Pleistocenic mtDNA diversity
Source: BMC Genomics. 2015 Feb 14;16(1):70. doi: 10.1186/s12864-014-1201-x (PMC4342813; doi:10.1186/s12864-014-1201-x)
Supplement: Additional file 1: — Origin of the 324 East Timor sample donors. East Timor and its 13 districts are shown. The diameter of every circle is proportional to the number of samples deriving from each district (see legend). The asterisk indicates the capital, Díli. The Díli district includes Ataúro island. The striped area is part of Indonesia. For a detailed list, see Additional file 2. [file 12864_2014_1201_MOESM1_ESM.pdf]

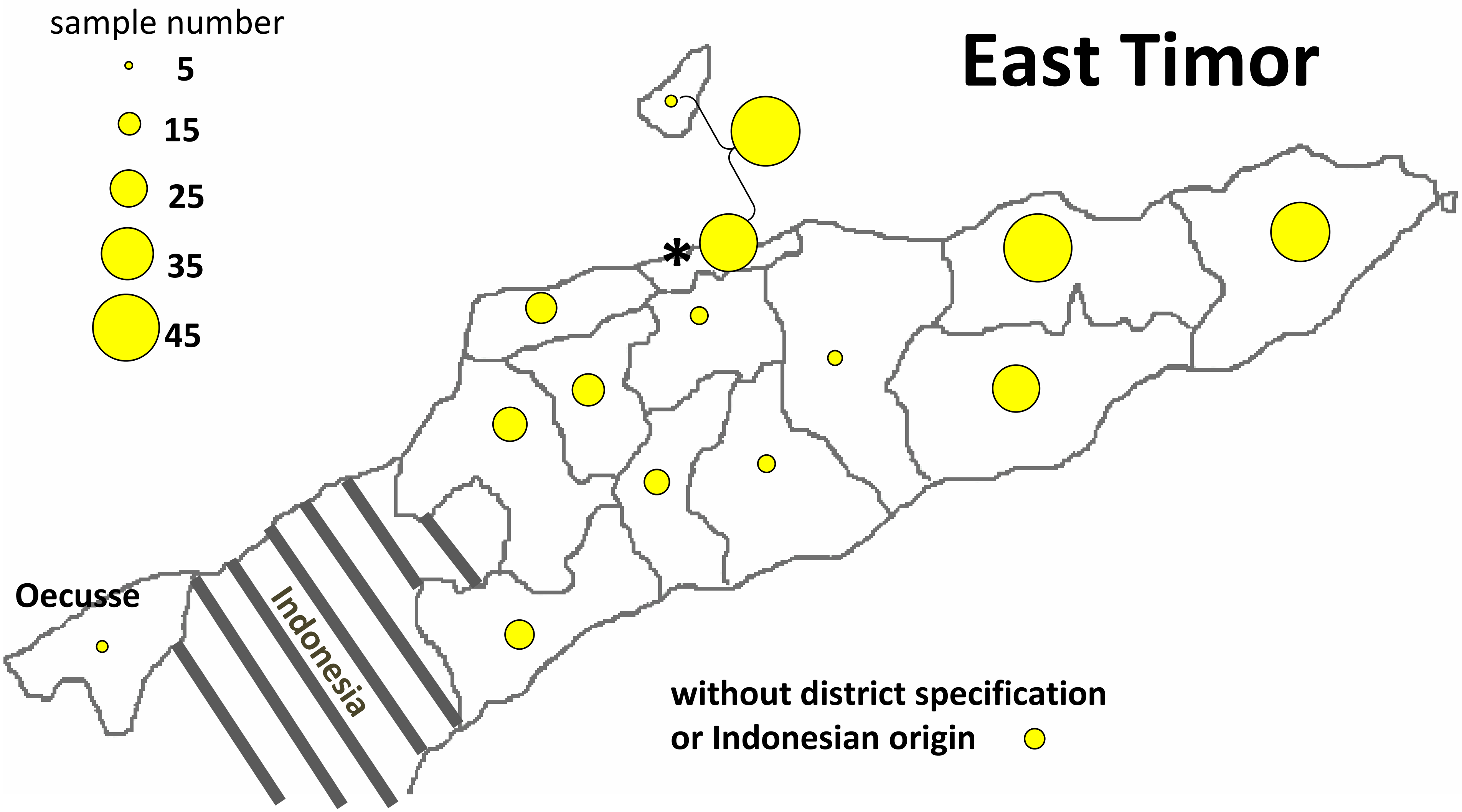
sample number
5
15
25
35
45
East Timor
Oecusse
Indonesia
without district specification
or Indonesian origin
