# Supplementary material for: Human settlement history between Sunda and Sahul: a focus on East Timor (Timor-Leste) and the Pleistocenic mtDNA diversity
Source: BMC Genomics. 2015 Feb 14;16(1):70. doi: 10.1186/s12864-014-1201-x (PMC4342813; doi:10.1186/s12864-014-1201-x)
Supplement: Additional file 2: — Detailed origin of the 324 East Timor sample donors. The district of origin is listed for each donor. n.a. = not available. [file 12864_2014_1201_MOESM2_ESM.pdf]

Additional file 2: Detailed origin of the 324 East Timor sample donors

sample name	District of origin
ET001	Ainaro
ET002	Ainaro
ET003	Baucau
ET004	Liquica
ET005	Baucau#
ET006	Lautem
ET007	Manatuto
ET008	Lautem
ET009	Ermera
ET010	Cova Lima
ET011	Baucau
ET012	Bobonaro
ET013	Bobonaro
ET014	Liquica
ET015	Ainaro#
ET016	Cova Lima
ET017	Cova Lima
ET018	Dili#
ET019	Cova Lima
ET020	Ainaro
ET021	Baucau
ET022	Lautem
ET023	Bobonaro
ET024	Bobonaro
ET025	Baucau#
ET026	Ainaro
ET027	Dili
ET028	Manatuto
ET029*	Indonesia (West Timor)#
ET030	Cova Lima#
ET031	Dili
ET032	Dili#
ET033	Bobonaro
ET034	Lautem
ET035	Bobonaro
ET036	Lautem
ET037	Lautem
ET038	Liquica
ET039	Manufahi
ET040	Lautem
ET041	Bobonaro
ET042	Baucau
ET043	Oecusse
ET044	Ainaro
ET045	Cova Lima
ET046	Viqueque
ET047	Baucau
ET048	Liquica
ET049	Liquica#
ET050	Ermera
ET051	Oecusse
ET052	Cova Lima
ET053	Atauro
ET054	Dili#
ET055	Baucau
ET056	Ainaro#
ET057	Baucau
ET058	Manufahi
ET059	Baucau
ET060	Viqueque
ET061	Baucau
ET062	Lautem
ET063*	Oecusse
ET064	Baucau
ET065	Baucau
ET066	Lautem
ET067	Cova Lima
ET068	Viqueque
ET069	Baucau#
ET070	Liquica
ET071	Ainaro
ET072	Viqueque
ET073	Dili
ET074	Lautem
ET075	Manatuto
ET076	Manatuto
ET077	Dili
ET078	Manatuto
ET079	Lautem
ET080	Baucau
ET081	Lautem
ET082	Ermera
ET083	Viqueque
ET084	Dili
ET085	Baucau
ET086	Aileu
ET087	Manatuto
ET088	Cova Lima
ET089	Ainaro
ET090	Ermera
ET091	Lautem
ET092	Liquica
ET093	Baucau
ET094	Ainaro
ET095	Baucau
ET096	Oecusse#
ET097	Lautem
ET098	Dili
ET099	Atauro
ET100	Cova Lima
ET101	Ermera
ET102	Lautem
ET103	Cova Lima
ET104	Ermera
ET105	Oecusse
ET106	Baucau
ET107	Oecusse
ET108	Baucau
ET109	Baucau
ET110	Viqueque
ET111	Ermera
ET112	Viqueque
ET113	Viqueque
ET114	Ainaro

ET115	Baucau
ET116	Bobonaro
ET117	Liquica#
ET118	Bobonaro#
ET119	Liquica
ET120	Ainaro
ET121	Viqueque
ET122	Cova Lima
ET123	Atauro
ET124	Atauro
ET125	Viqueque#
ET126	Baucau
ET127	Ainaro
ET128	Aileu
ET129	Bobonaro
ET130	Dili
ET131	Dili
ET132	Liquica
ET133	Lautem
ET134	Ainaro
ET135	Baucau
ET136	Baucau
ET137	Dili
ET138	Viqueque#
ET139	Aileu
ET140	Dili#
ET141	Manatuto
ET142	Lautem
ET143	Baucau
ET144	Bobonaro
ET145	Viqueque
ET146	Bobonaro
ET147	Dili
ET148	Ermera
ET149	Viqueque#
ET150	Manatuto
ET151	Bobonaro
ET152	Bobonaro
ET153	Bobonaro
ET154	Baucau
ET155	Cova Lima
ET156	Ermera
ET157	Ermera
ET158	Oecusse
ET159	Manufahi
ET160	Dili
ET161	Cova Lima
ET162*	Dili
ET163	Lautem
ET164	Viqueque
ET165	Cova Lima
ET166	Aileu
ET167	Dili
ET168	Cova Lima
ET169	Lautem
ET170	Baucau#
ET171	Lautem
ET172	Lautem
ET173	Lautem#
ET174	Dili
ET175	Viqueque
ET176	Atauro
ET177	Dili
ET178	Dili#
ET179	Viqueque
ET180	Baucau
ET181	Manufahi#
ET182	Lautem
ET183	Lautem
ET184	Viqueque
ET185	Lautem
ET186	n.a.
ET187	Bobonaro
ET188	Dili
ET189	Lautem
ET190	Ainaro
ET191	Baucau
ET192	Oecusse
ET193	Lautem
ET194	Baucau
ET195	Viqueque
ET196§	Lautem
ET197	Lautem
ET198	Viqueque
ET199	Dili
ET200	Aileu
ET201	Dili
ET202	Aileu
ET203	Viqueque
ET204	Liquica
ET205	Ermera
ET206	Aileu
ET207	Ermera#
ET208	Atauro
ET209	Cova Lima
ET210	Ainaro
ET211	n.a.
ET212	Bobonaro
ET213	n.a.
ET214*	Ermera
ET215	Manufahi
ET216	Liquica
ET217	Liquica
ET218	Bobonaro
ET219	Ermera
ET220	Viqueque
ET221	Liquica
ET222	Lautem
ET223	Ermera
ET224	Manatuto
ET225	Viqueque
ET226	Bobonaro
ET227	Baucau
ET228	Baucau
ET229	Liquica
ET230	Viqueque
ET231	Viqueque

ET232	Baucau
ET233	Manufahi
ET234	Liquica
ET235	Ermera
ET236	Aileu
ET237*	Bobonaro
ET238	Ermera
ET239	Viqueque#
ET240	Manufahi
ET241	Dili
ET242	Dili
ET243	Ermera
ET244	Ermera
ET245	Liquica#
ET246	Dili#
ET247	n.a.
ET248	Viqueque#
ET249	Ainaro
ET250	Baucau#
ET251	Bobonaro
ET252	Baucau
ET253	Dili#
ET254	Atauro
ET255	Baucau#
ET256	Dili
ET257	Baucau
ET258	Lautem
ET259	Dili
ET260	Manatuto
ET261	Dili
ET262	Aileu#
ET263	Viqueque
ET264	Baucau#
ET265	Baucau
ET266	Baucau
ET267	Dili
ET268	Lautem#
ET269	Dili
ET270	Baucau
ET271	Dili
ET272	Dili#
ET273	Dili
ET274	Viqueque
ET275	Baucau#
ET276	Liquica
ET277	Dili
ET278	Bobonaro
ET279	Lautem
ET280	Dili
ET281	Viqueque
ET282	Baucau
ET283	Baucau
ET284	Dili
ET285	Bobonaro
ET286	Bobonaro
ET287	Lautem
ET288	Lautem#
ET289	Ermera
ET290	Ermera
ET291	Liquica
ET292	Liquica
ET293	Ermera
ET294	Lautem
ET295	Ainaro
ET296	Manufahi
ET298	Manufahi
ET299	Aileu
ET300	Aileu
ET301	Baucau
ET302	Viqueque
ET303	Viqueque
ET304	Manufahi
ET305	Baucau
ET306	Liquica
ET307	Lautem
ET308	Manufahi
ET309	Manufahi
ET310	Viqueque#
ET311	Dili
ET312	Lautem
ET313	Baucau
ET314	Atauro
ET315	Viqueque
ET316	Ainaro
ET317	Baucau
ET318	Baucau
ET319	Cova Lima
ET320	Aileu
ET321	Cova Lima
ET322	Liquica
ET323	Indonesia (Flores)
ET324	Lautem
ET325	Lautem

n.a.= not available
* maternal ancestry from West Timor (Indonesia)
§ maternal ancestry from Java (Indonesia)
# ancestry information not complete over three generations
