# Supplementary material for: Human settlement history between Sunda and Sahul: a focus on East Timor (Timor-Leste) and the Pleistocenic mtDNA diversity
Source: BMC Genomics. 2015 Feb 14;16(1):70. doi: 10.1186/s12864-014-1201-x (PMC4342813; doi:10.1186/s12864-014-1201-x)
Supplement: Additional file 3: — CR profiles, haplogroups and GenBank accession numbers for the 324 East Timor samples. Haplogroups according to [49], build 16. Differences are relative to the rCRS [50]. Bases are indicated according to the IUBMB nucleotide code. The prefix @ indicates the reversion of a mutation occurring earlier in the phylogeny. Differences indicating potentially novel, not (yet) defined haplogroups are indicated as suffixes. [file 12864_2014_1201_MOESM3_ESM.pdf]

Additional file 3: CR profiles, haplogroups and GenBank accession numbers for the 324 East Timor samples

range: nps 16024-16569 1-576

GenBank accession	sample	haplogroup																					
KJ655599	ET190	B4-146-16129	16129A	16182C	16183C	16189C	16217C	16261T	16519C	73G	146C	263G	309.1C	309.2C	315.1C	523DEL	524DEL
KJ655678	ET103	B4-146-16129	16129A	16182C	16183C	16189C	16217C	16261T	16519C	73G	146C	263G	309.1C	309.2C	315.1C	523DEL	524DEL
KJ655594	ET026	B4-146-16129	16129A	16182C	16183C	16189C	16217C	16261T	16519C	73G	146C	263G	315.1C	523DEL	524DEL
KJ655856	ET068	B4-146-16129	16129A	16182C	16183C	16189C	16217C	16261T	16519C	73G	146C	263G	309.1C	315.1C	523DEL	524DEL
KJ655814	ET141	B4a1	16182C	16183C	16189C	16217C	16261T	16519C	73G	146C	263G	315.1C	523DEL	524DEL
KJ655630	ET011	B4a1	16182C	16183C	16189C	16217C	16261T	16519C	73G	146C	263G	309.1C	315.1C	523DEL	524DEL
KJ655699	ET280	B4a1	16182C	16183C	16189C	16217C	16261T	16519C	73G	146C	263G	309.1C	309.2C	309.3C	315.1C	523DEL	524DEL
KJ655639	ET228	B4a1	16182C	16183C	16189C	16217C	16261T	16519C	73G	146C	263G	309.1C	315.1C	523DEL	524DEL
KJ655647	ET059	B4a1	16182C	16183C	16189C	16217C	16261T	16519C	73G	146C	263G	309.1C	309.2C	309.3C	315.1C	523DEL	524DEL
KJ655872	ET250	B4a1	16182C	16183C	16189C	16217C	16261T	16519C	73G	146C	263G	309.1C	315.1C	523DEL	524DEL
KJ655688	ET161	B4a1	16182C	16183C	16189C	16217C	16261T	16519C	73G	146C	263G	309.1C	309.2C	315.1C	523DEL	524DEL
KJ655671	ET285	B4a1	16182C	16183C	16189C	16217C	16261T	16519C	73G	146C	263G	309.1C	309.2C	315.1C	523DEL	524DEL
KJ655865	ET302	B4a1	16182C	16183C	16189C	16217C	16261T	16519C	73G	146C	263G	309.1C	315.1C	523DEL	524DEL
KJ655612	ET314	B4a1	16182C	16183C	16189C	16217C	16261T	16519C	73G	146C	152C	263G	315.1C	523DEL	524DEL
KJ655881	ET237	B4a1	16182C	16183C	16189C	16217C	16261T	16519C	73G	146C	184A	263G	309.1C	315.1C	523DEL	524DEL
KJ655624	ET109	B4a1	16182C	16183C	16189C	16203G	16217C	16261T	16519C	73G	146C	263G	315.1C	523DEL	524DEL
KJ655603	ET015	B4a1	16093C	16182C	16183C	16189C	16217C	16261T	16519C	73G	146C	263G	309DEL	315.1C	523DEL	524DEL
KJ655770	ET102	B4a1	16179T	16182C	16183C	16189C	16217C	16261T	16519C	73G	146C	263G	315.1C	523DEL	524DEL
KJ655608	ET053	B4a1	16182C	16183C	16188T	16189C	16217C	16261T	16325C	16519C	73G	146C	263G	309.1C	309.2C	315.1C	523DEL	524DEL
KJ655704	ET032	B4a1	16092C	16182C	16183C	16189C	16217C	16261T	16311C	16519C	73G	146C	263G	309.1C	309.2C	315.1C	523DEL	524DEL
KJ655818	ET075	B4a1-16239	16182C	16183C	16189C	16217C	16239T	16261T	16519C	73G	146C	263G	309.1C	309.2C	315.1C	523DEL	524DEL
KJ655825	ET296	B4a1-16239	16182C	16183C	16189C	16217C	16239T	16261T	16519C	73G	146C	263G	309.1C	309.2C	309.3C	315.1C	523DEL	524DEL
KJ655790	ET040	B4a1a3a	16182C	16183C	16189C	16217C	16223T	16261T	16519C	73G	146C	263G	309.1C	315.1C	523DEL	524DEL
KJ655840	ET107	B4a1a3a	16182C	16183C	16189C	16217C	16223T	16261T	16519C	73G	146C	263G	309.1C	309.2C	315.1C	523DEL	524DEL
KJ655634	ET106	B4a1a3a	16182C	16183C	16189C	16217C	16223T	16261T	16519C	73G	146C	263G	309.1C	309.2C	315.1C	523DEL	524DEL
KJ655739	ET205	B4a1a3a	16182C	16183C	16189C	16217C	16223T	16261T	16519C	73G	146C	263G	309.1C	315.1C	523DEL	524DEL
KJ655827	ET159	B4a1a3a	16182C	16183C	16189C	16217C	16223T	16261T	16519C	73G	131C	146C	176G	263G	310C	314DEL	315DEL	316DEL	317DEL	319C	456T	523DEL	524DEL
KJ655819	ET087	B4a1a1	16182C	16183C	16189C	16217C	16247G	16261T	16519C	73G	146C	263G	309.1C	315.1C	523DEL	524DEL
KJ655593	ET295	B4a1a1	16182C	16183C	16189C	16217C	16247G	16261T	16519C	73G	146C	263G	309.1C	309.2C	315.1C	523DEL	524DEL
KJ655610	ET124	B4a1a1	16182C	16183C	16189C	16217C	16247G	16261T	16519C	73G	146C	263G	315.1C	523DEL	524DEL
KJ655635	ET135	B4a1a1	16182C	16183C	16189C	16217C	16247G	16261T	16519C	73G	146C	263G	309.1C	315.1C	523DEL	524DEL
KJ655689	ET165	B4a1a1	16182C	16183C	16189C	16217C	16247G	16261T	16519C	73G	146C	263G	309.1C	309.2C	315.1C	523DEL	524DEL
KJ655611	ET254	B4a1a1	16182C	16183C	16189C	16217C	16247G	16261T	16519C	73G	146C	263G	315.1C	523DEL	524DEL
KJ655843	ET043	B4a1a1	16182C	16183C	16189C	16217C	16247G	16261T	16519C	73G	146C	263G	309.1C	315.1C	523DEL	524DEL
KJ655773	ET193	B4a1a1	16182C	16183C	16189C	16217C	16247G	16261T	16519C	73G	146C	263G	309.1C	315.1C	523DEL	524DEL
KJ655683	ET019	B4a1a1	16182C	16183C	16189C	16217C	16247G	16261T	16519C	73G	146C	263G	309.1C	309.2C	309.3C	315.1C	523DEL	524DEL
KJ655799	ET291	B4a1a1	16182C	16183C	16189C	16217C	16247G	16261T	16519C	73G	146C	263G	309.1C	309.2C	315.1C	456T	523DEL	524DEL
KJ655656	ET151	B4a1a1	16182C	16183C	16189C	16217C	16239T	16247G	16261T	16519C	73G	146C	263G	309.1C	309.2C	315.1C	523DEL	524DEL
KJ655636	ET180	B4a1a1	16182C	16183C	16189C	16217C	16247G	16261T	16274A	16519C	73G	146C	263G	309.1C	309.2C	315.1C	523DEL	524DEL
KJ655720	ET259	B4a1a1	16086C	16182C	16183C	16189C	16217C	16247G	16261T	16519C	73G	146C	204C	207A	263G	309.1C	309.2C	315.1C	523DEL	524DEL
KJ655863	ET225	B4a1a1	16182C	16183C	16189C	16217C	16247G	16261T	16311C	16519C	73G	146C	263G	309.1C	309.2C	315.1C	523DEL	524DEL
KJ655726	ET256	B4a1a1-214	16178C	16182C	16183C	16189C	16217C	16247G	16261T	16519C	73G	146C	214G	263G	309.1C	309.2C	315.1C	523DEL	524DEL
KJ655875	ET211	B4a1a1-214	16182C	16183C	16189C	16217C	16247G	16261T	16519C	73G	146C	214G	263G	309.1C	309.2C	309.3C	315.1C	523DEL	524DEL
KJ655782	ET142	B4b1-207	16136C	16183C	16189C	16193.1C	16217C	16519C	73G	207A	263G	309.1C	309.2C	315.1C	499A	523DEL	524DEL
KJ655659	ET041	B4b1-207	16136C	16183C	16189C	16193.1C	16217C	16519C	73G	207A	263G	309.1C	309.2C	315.1C	499A	523DEL	524DEL
KJ655668	ET187	B4b1-207	16136C	16183C	16189C	16193.1C	16217C	16519C	73G	207A	263G	309.1C	309.2C	315.1C	499A	523DEL	524DEL
KJ655760	ET062	B4b1-207	16136C	16183C	16189C	16193.1C	16217C	16519C	73G	207A	263G	315.1C	499A	523DEL	524DEL
KJ655742	ET219	B4b1-207	16136C	16182C	16183C	16189C	16217C	16519C	73G	207A	263G	309.1C	309.2C	315.1C	499A	523DEL	524DEL
KJ655871	ET303	B4b1-207	16136C	16145A	16183C	16189C	16193.1C	16217C	16519C	73G	207A	263G	309.1C	309.2C	315.1C	499A	523DEL	524DEL
KJ655805	ET048	B4b1-207	16136C	16182C	16183C	16189C	16217C	16456A	16519C	16555C	73G	207A	263G	309.1C	309.2C	315.1C	499A	523DEL	524DEL
KJ655809	ET216	B4b1-207	16136C	16182C	16183C	16189C	16217C	16519C	73G	152C	207A	263G	309.1C	309.2C	315.1C	499A	523DEL	524DEL
KJ655741	ET214	B4c1b2a2	16140C	16182C	16183C	16189C	16217C	16274A	16335G	16519C	73G	146C	150T	195C	263G	309.1C	315.1C	523DEL	524DEL
KJ655648	ET136	B4c1b2a2	16140C	16182C	16183C	16189C	16217C	16274A	16335G	16519C	73G	146C	150T	195C	263G	309.1C	315.1C
KJ655776	ET288	B4c1b2a2	16140C	16182C	16183C	16189C	16217C	16274A	16335G	16519C	73G	146C	150T	195C	263G	309.1C	309.2C	315.1C
KJ655651	ET266	B4c1b2a2	16140C	16182C	16183C	16189C	16217C	16274A	16335G	16519C	73G	146C	150T	195C	263G	309.1C	315.1C
KJ655690	ET319	B4c1b2a2	16140C	16182C	16183C	16189C	16217C	16274A	16335G	73G	146C	150T	195C	234R	263G	315.1C
KJ655733	ET050	B4c1b2a2-547	16140C	16182C	16183C	16189C	16217C	16274A	16335G	16519C	73G	146C	150T	195C	263G	309.1C	309.2C	315.1C	547G
KJ655708	ET084	B4c1b2a2-547	16140C	16182C	16183C	16189C	16217C	16274A	16335G	16519C	73G	146C	150T	195C	263G	309.1C	309.2C	315.1C	547G
KJ655758	ET008	B4c1b2a2-204-16257	16140C	16182C	16183C	16189C	16217C	16257T	16274A	16335G	16519C	73G	146C	150T	195C	204C	263G	309.1C	309.2C	315.1C
KJ655784	ET172	B4c1b2a2-204-16257	16140C	16182C	16183C	16189C	16217C	16257T	16274A	16335G	16519C	73G	146C	150T	195C	204C	263G	309.1C	315.1C
KJ655837	ET063	B4c2	16147T	16183C	16184A	16189C	16217C	16235G	16519C	73G	263G	309.1C	315.1C

KJ655670	ET278	B4c2	16147T	16183C	16184A	16189C	16217C	16235G	16519C	73G	263G	309.1C	315.1C
KJ655638	ET194	B5b-152	16129A	16140C	16183C	16189C	16193.1C	16243C	16519C	73G	103A	152C	203A	204C	263G	309.1C	315.1C	523DEL	524DEL
KJ655792	ET182	B5b-152	16037G	16140C	16183C	16189C	16193.1C	16243C	16519C	73G	103A	152C	263G	309.1C	315.1C	523DEL	524DEL
KJ655709	ET098	B5b-152	16140C	16183C	16189C	16193.1C	16243C	16362Y	16519C	73G	103A	152C	263G	309.1C	315.1C	523DEL	524DEL
KJ655769	ET287	B5b-152	16140C	16183C	16189C	16193.1C	16213A	16243C	16519C	73G	103A	152C	263G	309.1C	315.1C	523DEL	524DEL
KJ655813	ET092	B5b1c	16140C	16183C	16189C	16193.1C	16243C	16519C	73G	103A	152C	204C	263G	315.1C	523DEL	524DEL
KJ655804	ET004	B5b1c	16140C	16183C	16189C	16193.1C	16243C	16519C	73G	103A	152C	204C	263G	309.1C	309.2C	315.1C	523DEL	524DEL
KJ655662	ET212	B5b1c	16140C	16183C	16189C	16193.1C	16243C	16519C	73G	103A	152C	195C	204C	263G	309.1C	315.1C	523DEL	524DEL
KJ655873	ET035	B5b1c	16140C	16183C	16189C	16243C	16263C	16519C	73G	103A	152C	204C	263G	309.1C	309.2C	315.1C	523DEL	524DEL
KJ655622	ET283	B5b1c	16140C	16183C	16189C	16193.1C	16243C	16519C	73G	103A	152C	204C	207A	263G	309.1C	309.2C	315.1C	523DEL	524DEL
KJ655747	ET148	B5b1c-16311	16140C	16183C	16189C	16193.1C	16243C	16311C	16519C	73G	103A	152C	204C	263G	315.1C	523DEL	524DEL
KJ655595	ET071	B5b1c-16311-16500	16140C	16183C	16189C	16193.1C	16243C	16311C	16500C	16519C	73G	103A	152C	204C	263G	315.1C	523DEL	524DEL
KJ655746	ET111	B5b1c-16311-16500	16140C	16183C	16189C	16193.1C	16243C	16311C	16500C	16519C	73G	103A	152C	204C	263G	315.1C	523DEL	524DEL
KJ655762	ET074	D5b1c1-16092	16092C	16148T	16183C	16189C	16193.1C	16223T	16362C	16519C	73G	150T	152C	185A	225R	263G	309.1C	315.1C	456T	489C	523DEL	524DEL
KJ655767	ET185	D5b1c1-16092-189	16092C	16148T	16183C	16189C	16193.1C	16223T	16362C	16519C	73G	150T	152C	185A	189G	263G	309.1C	315.1C	456T	489C	523DEL	524DEL
KJ655788	ET312	D5b1c1-16092-189	16092C	16148T	16183C	16189C	16193.1C	16223T	16362C	16519C	73G	150T	152C	185A	189G	263G	309.1C	315.1C	456T	489C	523DEL	524DEL
KJ655675	ET052	D5b1c1-16092-16239	16092C	16148T	16183C	16189C	16193.1C	16223T	16239T	16297C	16362C	16519C	73G	150T	152C	185R	263G	309.1C	315.1C	456T	489C	523DEL	524DEL
KJ655833	ET304	D5b1c1-16239	16148T	16182C	16183C	16189C	16223T	16239T	16319A	16362C	16519C	73G	150T	152C	185A	263G	309.1C	309.2C	315.1C	456T	489C	523DEL	524DEL
KJ655590	ET002	E1a1a	16223T	16291T	16362C	16390A	16519C	73G	263G	309.1C	315.1C	489C
KJ655783	ET169	E1a1a	16223T	16291T	16362C	16390A	16519C	73G	263G	315.1C	489C
KJ655807	ET070	E1a1a	16223T	16291T	16362C	16390A	16519C	73G	263G	315.1C	489C
KJ655597	ET134	E1a1a	16223T	16291T	16362C	16390A	16519C	73G	263G	309.1C	315.1C	489C
KJ655868	ET125	E1a1a	16223T	16291T	16362C	16390A	16519C	73G	263G	309.1C	315.1C	489C
KJ655698	ET261	E1a1a	16223T	16291T	16362C	16390A	16519C	73G	263G	309.1C	315.1C	489C
KJ655663	ET226	E1a1a	16223T	16291T	16362C	16390A	16519C	73G	263G	315.1C	489C
KJ655734	ET082	E1a1a	16223T	16291T	16362C	16390A	16519C	73G	263G	309.1C	315.1C	489C
KJ655800	ET322	E1a1a	16223T	16291T	16362C	16390A	16519C	73G	263G	315.1C	489C
KJ655684	ET030	E1a1a	16223T	16291T	16362C	16390A	16519C	16527T	73G	263G	315.1C	489C
KJ655867	ET315	E1a1a-125-127	16223T	16291T	16352C	16362C	16390A	16519C	73G	125C	127C	153G	263G	315.1C	489C
KJ655787	ET307	E1a1a-125-127-235-257	16223T	16291T	16362C	16390A	16519C	73G	125C	127C	235G	257G	263G	309.1C	315.1C	489C
KJ655785	ET222	E1a1a-125-127-235-257	16223T	16291T	16362C	16390A	16519C	73G	125C	127C	235G	257G	263G	309.1C	315.1C	489C
KJ655854	ET203	E1a1a-125-127-235-257-292	16223T	16291T	16362C	16390A	16519C	73G	125C	127C	235G	257G	263G	292C	309.1C	315.1C	489C
KJ655847	ET239	E1a1a-125-127-235-257-292	16223T	16291T	16362C	16390A	16519C	73G	125C	127C	235G	257G	263G	292C	309.1C	315.1C	489C
KJ655645	ET085	E1a1a-125-127-235-257-292	16223T	16291T	16362C	16390A	16519C	73G	125C	127C	235G	257G	263G	292C	309.1C	315.1C	489C
KJ655832	ET308	E1a1a-257	16223T	16291T	16362C	16390A	16519C	73G	257G	263G	309.1C	315.1C	489C
KJ655786	ET258	E1a1a-257	16223T	16291T	16362C	16390A	16519C	73G	257G	263G	309.1C	315.1C	489C
KJ655736	ET101	E1a1a-257	16223T	16291T	16362C	16390A	16519C	73G	257G	263G	309.1C	315.1C	489C
KJ655817	ET028	E1a1a-257	16223T	16291T	16362C	16390A	16519C	73G	257G	263G	309.1C	315.1C	489C
KJ655771	ET171	E1a1a-195	16223T	16291T	16362C	16390A	16519C	73G	195C	263G	309.1C	315.1C	489C
KJ655764	ET091	E1a1a-195	16223T	16291T	16362C	16390A	16519C	73G	195C	263G	309.1C	315.1C	489C	574C
KJ655885	ET069	E1a1a-195	16223T	16291T	16362C	16390A	16519C	73G	195C	263G	309.1C	315.1C	489C
KJ655853	ET113	E1a1a-195-16189	16189C	16193.1C	16223T	16291T	16362C	16390A	16519C	73G	195C	263G	309.1C	315.1C	489C
KJ655861	ET184	E1a1a-195-16189	16189C	16223T	16291T	16362C	16390A	16519C	73G	195C	263G	309.1C	315.1C	489C
KJ655789	ET006	E1a1a-195-16189	16189C	16223T	16291T	16362C	16390A	16519C	73G	195C	263G	309.1C	315.1C	489C
KJ655617	ET108	E1a1a-195-16189	16189C	16223T	16291T	16362C	16390A	16519C	73G	150T	185R	195C	263G	309.1C	315.1C	489C
KJ655706	ET073	E1a1a-195-16189	16183C	16189C	16223T	16291T	16362C	16390A	16519C	73G	195C	263G	309.1C	315.1C	489C
KJ655616	ET057	E1a1a-195-16189	16189C	16193.1C	16193.2(	16223T	16291T	16362C	16390A	16519C	73G	195C	263G	309.1C	315.1C	489C
KJ655756	ET294	E1a1a-195-16189	16183C	16189C	16193.1(	16223T	16291T	16301T	16362C	16390A	16519C	73G	195C	263G	309.1C	315.1C	489C
KJ655772	ET189	E1a1a-195-16189-16274	16183C	16189C	16193.1(	16223T	16274A	16291T	16362C	16390A	16519C	73G	195C	263G	309.1C	309.2C	315.1C	489C
KJ655793	ET325	E1a1a-195-16189-16274	16183C	16189C	16193.1(	16223T	16274A	16291T	16362C	16390A	16519C	73G	195C	263G	309.1C	315.1C	489C
KJ655757	ET324	E1a1a-195-16189-16274	16183C	16189C	16193.1(	16223T	16274A	16291T	16362C	16390A	16519C	73G	195C	263G	309.1C	315.1C	489C
KJ655842	ET192	E1a1a-16189	16189Y	16223T	16291T	16362C	16390A	16519C	73G	263G	309.1C	315.1C	489C
KJ655650	ET255	E1a1a-16189	16183C	16189C	16193.1(	16223T	16291T	16362C	16390A	16519C	73G	263G	315.1C	489C
KJ655619	ET149	E1a1a-16189	16189C	16223T	16291T	16362C	16390A	16519C	73G	263G	309.1C	315.1C	489C
KJ655627	ET065	E1a1a-16093-16189	16093C	16189Y	16223T	16291T	16362C	16390A	16465T	16519C	73G	263G	309.1C	315.1C	489C
KJ655882	ET140	E1a1a-16093	16093C	16223T	16291T	16362C	16390A	16519C	73G	263G	309.1C	315.1C	489C	513R
KJ655866	ET310	E1a1a-16093	16093C	16223T	16291T	16362C	16390A	16519C	73G	263G	309.1C	309.2C	315.1C	489C
KJ655712	ET147	E1a2	16223T	16362C	16390A	16519C	73G	263G	309.1C	315.1C	373G	489C
KJ655621	ET274	E1a2-16305	16223T	16305G	16362C	16390A	16519C	73G	263G	309.1C	315.1C	373G	489C
KJ655633	ET093	E1a2-16305	16223T	16305G	16362C	16390A	16519C	73G	263G	309.1C	315.1C	373G	489C
KJ655850	ET281	E1a2-16305	16223T	16305G	16362C	16390A	16519C	73G	263G	309.1C	315.1C	373G	489C
KJ655583	ET202	E1a2-16261	16069T	16223T	16261T	16362C	16390A	16519C	73G	263G	309.1C	315.1C	373G	489C
KJ655655	ET116	E1b-16261	16223T	16261T	16362C	16390A	16519C	73G	152C	263G	309.1C	315.1C	489C
KJ655587	ET128	E1b-16261	16223T	16261T	16362C	16390A	16519C	73G	152C	263G	315.1C	489C
KJ655586	ET320	E1b-16261	16223T	16261T	16362C	16390A	16519C	73G	152C	263G	315.1C	489C
KJ655803	ET306	E1b-16261	16223T	16261T	16362C	16390A	16519C	73G	152C	263G	309.1C	315.1C	489C
KJ655585	ET299	E1b-16261	16223T	16261T	16362C	16390A	16519C	73G	152C	263G	309.1C	315.1C	489C

KJ655738	ET157	E1b-16261	16223T	16261T	16311C	16362C	16390A	16519C	73G	152C	199Y	263G	309.1C	315.1C	489C							
KJ655857	ET046	E1b-16261	16223T	16261T	16362C	16390A	16519C	73G	152C	263G	309.1C	315.1C	489C									
KJ655876	ET262	E2-16093-16215	16051G	16093C	16215G	16223T	16362C	16390A	73G	195C	263G	315.1C	489C	523DEL	524DEL							
KJ655717	ET201	E2-16093-16215	16051G	16093C	16215G	16223T	16362C	16390A	73G	195C	263G	315.1C	489C	523DEL	524DEL							
KJ655723	ET272	E2-16093-16215	16051G	16093C	16215G	16223T	16362C	16390A	73G	195C	263G	315.1C	489C	523DEL	524DEL	525T						
KJ655600	ET094	E2-16215	16051G	16215G	16223T	16362C	16390A	73G	195C	263G	315.1C	489C										
KJ655823	ET298	E2-16215	16051G	16215G	16223T	16362C	16390A	73G	195C	263G	315.1C	489C										
KJ655609	ET123	E2-16185	16051G	16185T	16223T	16362C	16390A	73G	195C	263G	315.1C	489C										
KJ655710	ET099	E2-16185	16051G	16185T	16223T	16362C	16390A	73G	195C	263G	315.1C	489C										
KJ655821	ET078	F1a1a	16108T	16129A	16162G	16172C	16304C	16519C	73G	207A	249DEL	263G	309.1C	315.1C	523DEL	524DEL						
KJ655815	ET076	F1a2-16311	16172C	16245T	16304C	16311C	16519C	73G	249DEL	263G	315.1C	523DEL	524DEL									
KJ655625	ET025	F1a3a	16129A	16172C	16304C	16311C	16519C	73G	249DEL	263G	309.1C	315.1C	523DEL	524DEL								
KJ655779	ET036	F1a3a	16129A	16172C	16304C	16311C	16519C	73G	249DEL	263G	309.1C	309.2C	315.1C	523DEL	524DEL	573.1C						
KJ655862	ET195	F1a3a	16129A	16172C	16304C	16311C	16519C	73G	249DEL	263G	309.1C	315.1C	523DEL	524DEL								
KJ655768	ET268	F1a3a	16129A	16172C	16304C	16311C	16519C	73G	249DEL	263G	315.1C	523DEL	524DEL									
KJ655778	ET022	F1a3a	16129A	16172C	16304C	16311C	16519C	73G	249DEL	263G	309.1C	315.1C	523DEL	524DEL								
KJ655729	ET131	F1a3a	16129A	16172C	16304C	16311C	16519C	73G	249DEL	263G	309.1C	315.1C	523DEL	524DEL								
KJ655646	ET042	F1a3a	16129A	16172C	16304C	16311C	16519C	73G	249DEL	263G	309.1C	315.1C	523DEL	524DEL								
KJ655614	ET003	F1a3a	16129A	16172C	16304C	16311C	16519C	73G	249DEL	263G	309.1C	315.1C	523DEL	524DEL								
KJ655846	ET230	F1a3a	16129A	16172C	16304C	16311C	16519C	73G	249DEL	263G	315.1C	523DEL	524DEL									
KJ655632	ET080	F1a3a	16129A	16172C	16304C	16311C	16519C	73G	249DEL	263G	315.1C	523DEL	524DEL									
KJ655761	ET066	F1a3a	16129A	16172C	16235G	16304C	16311C	16519C	73G	249DEL	263G	309.1C	315.1C	523DEL	524DEL							
KJ655855	ET220	F1a3a	16129A	16172C	16304C	16311C	16519C	73G	249DEL	263G	309.1C	309.2C	315.1C	523DEL	524DEL							
KJ655838	ET096	F1a3a-195	16129A	16172C	16304C	16311C	16519C	73G	195C	249DEL	263G	309.1C	315.1C	523DEL	524DEL							
KJ655874	ET118	F1a3a-195-444-16174	16129A	16172C	16174T	16304C	16311C	16519C	73G	195C	249DEL	263G	309.1C	315.1C	385R	444G	523DEL	524DEL				
KJ655665	ET013	F1a3a-195-444-16174	16129A	16172C	16174T	16304C	16311C	16519C	73G	195C	249DEL	263G	309.1C	309.2C	315.1C	444G	523DEL	524DEL				
KJ655753	ET323	F1a3a-195-16174	16129A	16172C	16174T	16304C	16311C	16519C	73G	195C	249DEL	263G	309.1C	315.1C	523DEL	524DEL						
KJ655718	ET241	F1a4a1	16129A	16172C	16294T	16304C	16362C	16519C	73G	152C	249DEL	263G	309.1C	315.1C	521DEL	522DEL	523DEL	524DEL				
KJ655802	ET221	F1a4a1	16129A	16172C	16294T	16304C	16362C	16519C	73G	152C	249DEL	263G	309.1C	315.1C	521DEL	522DEL	523DEL	524DEL				
KJ655878	ET117	F1a4a1	16129A	16172C	16294T	16304C	16362C	16519C	73G	152C	249DEL	263G	309.1C	309.2C	315.1C	521DEL	522DEL	523DEL	524DEL			
KJ655879	ET213	F1a4a1	16129A	16172C	16294T	16304C	16362C	16519C	73G	152C	249DEL	263G	309.1C	315.1C	521DEL	522DEL	523DEL	524DEL				
KJ655716	ET199	F1a4a1	16129A	16172C	16294T	16304C	16362C	16519C	73G	152C	249DEL	263G	309.1C	315.1C	521DEL	522DEL	523DEL	524DEL				
KJ655884	ET056	F1a4a1	16129A	16172C	16294T	16304C	16362C	16519C	73G	152C	249DEL	263G	309.1C	309.2C	315.1C	521DEL	522DEL	523DEL	524DEL			
KJ655669	ET218	F1a4a1	16129A	16172C	16294T	16304C	16362C	16519C	73G	152C	249DEL	263G	309.1C	315.1C	521DEL	522DEL	523DEL	524DEL				
KJ655766	ET163	F1a4a1	16129A	16172C	16294T	16304C	16362C	16519C	73G	152C	249DEL	263G	309.1C	315.1C	521DEL	522DEL	523DEL	524DEL				
KJ655618	ET115	F1a4a1	16129A	16172C	16294T	16304C	16362C	16519C	73G	152C	249DEL	263G	309.1C	315.1C	521DEL	522DEL	523DEL	524DEL				
KJ655730	ET160	F1a4a1	16129A	16172C	16294T	16304C	16362C	16519C	73G	152C	249DEL	263G	309.1C	315.1C	521DEL	522DEL	523DEL	524DEL				
KJ655822	ET150	F1a4a1	16129A	16172C	16294T	16304C	16362C	16519C	73G	152C	249DEL	263G	309.1C	309.2C	315.1C	521DEL	522DEL	523DEL	524DEL			
KJ655877	ET248	F1a4a1	16129A	16172C	16294T	16304C	16362C	16519C	73G	152C	249DEL	263G	309.1C	315.1C	521DEL	522DEL	523DEL	524DEL	534Y			
KJ655606	ET089	F1a4a1	16129A	16172C	16294T	16304C	16362C	16519C	73G	152C	249DEL	263G	309.1C	315.1C	521DEL	522DEL	523DEL	524DEL				
KJ655652	ET313	F1a4a1	16129A	16172C	16294T	16304C	16362C	16519C	73G	152C	249DEL	263G	309.1C	315.1C	521DEL	522DEL	523DEL	524DEL	573.1C			
KJ655836	ET051	F1a4a1	16129A	16172C	16294T	16304C	16362C	16519C	73G	152C	249DEL	263G	309.1C	315.1C	324T	521DEL	522DEL	523DEL	524DEL			
KJ655848	ET263	F1a4a1	16129A	16172C	16294T	16304C	16362C	16519C	73G	152C	234R	249DEL	263G	309.1C	315.1C	521DEL	522DEL	523DEL	524DEL			
KJ655735	ET090	F1a4a1	16129A	16172C	16294T	16304C	16362C	16519C	73G	152C	195Y	249DEL	263G	309.1C	315.1C	521DEL	522DEL	523DEL	524DEL			
KJ655623	ET301	F1a4a1	16129A	16172C	16294T	16304C	16362C	16519C	73G	152C	214R	249DEL	263G	309.1C	315.1C	521DEL	522DEL	523DEL	524DEL			
KJ655687	ET155	F1a4a1-146	16129A	16172C	16294T	16304C	16362C	16519C	73G	146C	152C	249DEL	263G	309.1C	315.1C	521DEL	522DEL	523DEL	524DEL			
KJ655674	ET016	F1a4a1-146	16129A	16172C	16294T	16304C	16362C	16519C	73G	146C	152C	249DEL	263G	309.1C	309.2C	315.1C	521DEL	522DEL	523DEL	524DEL		
KJ655691	ET321	F1a4a1-16189	16129A	16172C	16189C	16193.1C	16193.2C	16294T	16304C	16362C	16519C	73G	152C	249DEL	263G	309.1C	309.2C	315.1C	521DEL	522DEL	523DEL	524DEL
KJ655685	ET045	F1a4a1-16189	16129A	16172C	16189C	16294T	16304C	16362C	16519C	73G	152C	249DEL	263G	309.1C	315.1C	521DEL	522DEL	523DEL	524DEL			
KJ655607	ET176	F1a4a1-16239	16129A	16172C	16239T	16294T	16304C	16362C	16519C	73G	152C	249DEL	263G	315.1C	521DEL	522DEL	523DEL	524DEL				
KJ655598	ET316	F1a4a1-16239	16129A	16172C	16239T	16294T	16304C	16362C	16519C	73G	152C	249DEL	263G	315.1C	521DEL	522DEL	523DEL	524DEL				
KJ655680	ET168	F1a4a1-16320	16129A	16172C	16294T	16304C	16320T	16362C	16519C	73G	152C	249DEL	263G	309.1C	309.2C	315.1C	521DEL	522DEL	523DEL	524DEL		
KJ655795	ET292	F1a4a1-16320	16129A	16172C	16294T	16304C	16320T	16362C	16519C	73G	152C	249DEL	263G	309.1C	315.1C	521DEL	522DEL	523DEL	524DEL			
KJ655697	ET018	F1a4a1-16320	16129A	16172C	16294T	16304C	16320T	16362C	16519C	73G	152C	249DEL	263G	309.1C	315.1C	521DEL	522DEL	523DEL	524DEL			
KJ655831	ET240	F1a4a1-16320	16129A	16172C	16294T	16304C	16320T	16362C	16519C	73G	152C	249DEL	263G	309.1C	315.1C	521DEL	522DEL	523DEL	524DEL			
KJ655721	ET269	F1a4a1-16320	16129A	16172C	16294T	16304C	16320T	16362C	16519C	73G	152C	249DEL	263G	309.1C	309.2C	315.1C	521DEL	522DEL	523DEL	524DEL		
KJ655829	ET181	F3b1a-71DEL	16093C	16220C	16265G	16298C	16311C	16362C	71DEL	73G	150T	152C	249DEL	263G	315.1C							
KJ676774	ET126	M-16192-16274-16362 (D6a*)	16192T	16223T	16274A	16362C	73G	263G	309.1C	315.1C	489C											
KJ655763	ET079	M10-417-16256	16223T	16256T	16311C	16519C	73G	263G	315.1C	417A	489C	523DEL	524DEL	573.1C	573.2C	573.3C	573.4C	573.5C				
KJ676790	ET110	M-152-174-299DEL-16093-16311 (M21b*)	16093C	16223T	16311C	16519C	73G	152C	174T	263G	299DEL	315.1C	489C									
KJ655615	ET021	M-152-417-16311	16223T	16311C	16519C	73G	152C	263G	315.1C	417A	489C	523DEL	524DEL									
KJ655620	ET227	M-152-417-16311	16223T	16311C	16519C	73G	152C	263G	315.1C	417A	489C	523DEL	524DEL									
KJ676780	ET257	M-146-195-215-519-16129-16311-16319 (Q3*)	16129A	16223T	16311C	16319A	73G	146C	195C	215G	263G	309.1C	315.1C	489C	519G							
KJ655774	ET196	M71a2-345-@16223	16129A	16140C	16271C	73G	143A	146C	151T	263G	309.1C	315.1C	345T	489C								
KJ676788	ET215	M73a-16192-16239-16354 (M73a*)	16184A	16192T	16223T	16239T	16245Y	16278T	16354T	73G	263G	309.1C	315.1C	489C								
KJ655673	ET129	M73a-16192-16239-16354	16184A	16192T	16223T	16239T	16278T	16354T	73G	263G	309.1C	315.1C	489C									
KJ655887	ET144	M7c1	16223T	16362C	16519C	73G	146C	199C	263G	309.1C	315.1C	489C	523DEL	524DEL								

KJ655743	ET223	M7c1	16223T	16362C	16519C	73G	146C	199C	263G	309.1C	309.2C	315.1C	489C	523DEL	524DEL					
KJ655664	ET012	M7c1	16223T	16362C	16519C	73G	146C	199C	263G	315.1C	489C	523DEL	524DEL							
KJ655870	ET179	M7c1-520	16223T	16362C	16519C	73G	146C	199C	263G	315.1C	489C	520T	523DEL	524DEL						
KJ655845	ET198	M7c1-520	16223T	16362C	16519C	73G	146C	199C	263G	315.1C	489C	520T	523DEL	524DEL						
KJ655728	ET253	M7c1-520	16223T	16362C	16519C	73G	146C	199C	263G	315.1C	489C	520T	523DEL	524DEL						
KJ655883	ET247	M7c1-520	16223T	16362C	16519C	73G	146C	199C	263G	315.1C	489C	520T	523DEL	524DEL						
KJ655765	ET133	M7c1-520	16223T	16362C	16519C	73G	146C	199C	263G	315.1C	489C	520T	523DEL	524DEL						
KJ655727	ET246	M7c1-520	16223T	16362C	16519C	73G	146C	199C	263G	315.1C	489C	520T	523DEL	524DEL						
KJ655637	ET191	M7c1-520	16223T	16362C	16519C	73G	146C	199C	263G	309.1C	315.1C	489C	520T	523DEL	524DEL					
KJ655858	ET083	M7c1-520	16223T	16362C	16519C	73G	146C	199C	263G	309.1C	315.1C	489C	520T	523DEL	524DEL					
KJ655641	ET270	M7c1-520	16223T	16362C	16519C	73G	146C	199C	263G	309.1C	315.1C	489C	520T	523DEL	524DEL					
KJ655851	ET112	M7c1-520	16223T	16362C	16519C	73G	146C	199C	263G	309.1C	315.1C	489C	520T	523DEL	524DEL					
KJ655834	ET007	M7c1-520	16223T	16362C	16519C	73G	146C	199C	263G	309.1C	315.1C	489C	520T	523DEL	524DEL					
KJ655589	ET001	M7c1-16295	16223T	16295T	16362C	16519C	73G	146C	199C	263G	309.1C	309.2C	315.1C	489C	523DEL	524DEL				
KJ655755	ET183	M7c1-16295	16223T	16295T	16362C	16519C	73G	146C	199C	263G	309.1C	309.2C	315.1C	489C	523DEL	524DEL				
KJ655584	ET236	M7c1-16295	16223T	16295T	16362C	16519C	73G	146C	199C	263G	309.1C	315.1C	489C	523DEL	524DEL					
KJ655596	ET127	M7c1-16295	16223T	16295T	16362C	16519C	73G	146C	199C	263G	309.1C	315.1C	489C	523DEL	524DEL					
KJ655602	ET120	M7c1-16295	16223T	16295T	16362C	16519C	73G	146C	199C	263G	309.1C	315.1C	489C	523DEL	524DEL					
KJ655686	ET067	M7c1-16295	16223T	16295T	16362C	16519C	73G	146C	199C	263G	309.1C	315.1C	489C	523DEL	524DEL					
KJ655737	ET104	M7c1-16295	16223T	16295T	16362C	16519C	73G	146C	199C	263G	309.1C	315.1C	489C	523DEL	524DEL					
KJ655796	ET038	M7c1-16295	16223T	16295T	16362C	16519C	73G	146C	199C	263G	309.1C	315.1C	489C	523DEL	524DEL					
KJ655745	ET244	M7c1-16295	16223T	16295T	16362C	16519C	73G	146C	199C	263G	309.1C	315.1C	489C	523DEL	524DEL					
KJ655731	ET162	M7c1-16295	16223T	16295T	16362C	16519C	73G	146C	199C	263G	309.1C	315.1C	489C	523DEL	524DEL					
KJ655828	ET039	M7c1-16295	16223T	16295T	16362C	16519C	73G	146C	199C	263G	309.1C	315.1C	489C	523DEL	524DEL					
KJ655806	ET049	M7c1-16295	16223T	16295T	16362C	16519C	73G	146C	199C	263G	309.1C	315.1C	489C	523DEL	524DEL					
KJ655826	ET058	M7c1-16295	16223T	16295T	16362C	16519C	73G	146C	199C	263G	315.1C	489C	523DEL	524DEL						
KJ655703	ET031	M7c1-16295	16223T	16295T	16362C	73G	146C	199C	207A	263G	309.1C	315.1C	489C	523DEL	524DEL					
KJ655601	ET114	M7c1-16295	16223T	16295T	16362C	16519C	73G	146C	199C	217Y	263G	309.1C	315.1C	489C	523DEL	524DEL				
KJ655707	ET077	M7c1-16295	16223T	16292T	16295T	16362C	16468C	16519C	73G	146C	199C	263G	309.1C	315.1C	489C	523DEL	524DEL			
KJ655679	ET122	M7c1-16295	16223T	16295T	16309G	16362C	16519C	73G	146C	199C	263G	309.1C	315.1C	489C	523DEL	524DEL				
KJ655588	ET206	M7c1-16295	16185T	16223T	16295T	16362C	16519C	73G	146C	199C	263G	309.1C	315.1C	489C	523DEL	524DEL				
KJ655725	ET137	M7c1-152-16295	16223T	16295T	16362C	16519C	73G	146C	152C	199C	263G	309.1C	315.1C	489C	523DEL	524DEL				
KJ655880	ET029	M7c1-152-16295	16223T	16295T	16362C	16519C	73G	146C	152C	199C	263G	309.1C	315.1C	489C	523DEL	524DEL				
KJ655714	ET178	M7c1-16189-16295	16189Y	16223T	16295T	16362C	16519C	73G	146C	199C	263G	309.1C	315.1C	489C	523DEL	524DEL				
KJ655835	ET055	M7c1-16189-16295	16189C	16223T	16295T	16362C	16519C	73G	146C	199C	263G	315.1C	489C	523DEL	524DEL					
KJ655859	ET121	M7c1-16189-16295	16189C	16223T	16295T	16362C	16519C	73G	146C	199C	263G	315.1C	489C	523DEL	524DEL					
KJ655852	ET145	M7c1-16189-16295	16189C	16193.1C	16223T	16295T	16362C	16519C	73G	146C	199C	263G	309.1C	315.1C	489C	523DEL	524DEL			
KJ655626	ET061	M7c1-16189-16295	16189C	16193.1C	16223T	16295T	16362C	16519C	73G	146C	199C	263G	315.1C	489C	523DEL	524DEL				
KJ655631	ET047	M7c1-16189-16295	16189C	16193.1C	16223T	16295Y	16311C	16362C	16519C	73G	146C	199C	263G	315.1C	489C	523DEL	524DEL			
KJ655816	ET224	M7c1-16189-16295	16086C	16183C	16189C	16193.1C	16223T	16295T	16362C	16519C	73G	146C	199C	263G	309.1C	315.1C	489C	523DEL	524DEL	
KJ655653	ET317	M7c1-143-16189-16295	16189C	16223T	16295T	16362C	16519C	73G	143A	146C	199C	263G	309.1C	309.2C	315.1C	489C	523DEL	524DEL		
KJ655860	ET164	M7c1-143-16189-16295	16189C	16193.1C	16193.2(	16223T	16295T	16362C	16519C	73G	143A	146C	199C	263G	309.1C	309.2C	315.1C	489C	523DEL	524DEL
KJ655849	ET265	M7c1-143-16189-16295	16183C	16189C	16193.1(	16223T	16295Y	16362C	16519C	73G	143A	146C	199C	263G	309.1C	309.2C	315.1C	489C	523DEL	524DEL
KJ655844	ET060	M7c1-143-16189-16295	16183C	16189C	16193.1(	16223T	16295T	16362C	16519C	73G	143A	146C	199C	263G	309.1C	315.1C	489C	523DEL	524DEL	
KJ655666	ET033	N21a-16319-@16193	16223T	16291T	16319A	16519C	73G	150T	195C	263G	309.1C	315.1C	337DEL							
KJ655681	ET209	N21a-16319	16193T	16223T	16291T	16319A	16519C	73G	150T	195C	200G	263G	309.1C	315.1C	337DEL					
KJ676777	ET064	P1 (P1d*)	16176T	16266T	16357C	73G	212C	263G	315.1C											
KJ676782	ET139	P1-152-235-16156-16169-16311 (P1d*)	16189Y	16156A	16169T	16176T	16266T	16311C	16357C	73G	152C	212C	235G	263G	315.1C					
KJ676784	ET167	P1-152-235-16156-16169-16311 (P1d*)	16156A	16169T	16176T	16266T	16311C	16357C	73G	152C	212C	235G	263G	315.1C	524.1A	524.2C				
KJ676787	ET014	P1-16526 (P1d*)	16176T	16266T	16357C	16526A	73G	212C	263G	315.1C										
KJ676781	ET017	P1-16526 (P1d*)	16176T	16266T	16357C	16526A	73G	212C	263G	315.1C	524.1A	524.2C								
KJ676776	ET005	P1-152-246-508-16239-@212-@16176 ("P1e"*#)	16239T	16266T	16357C	73G	152C	246C	263G	315.1C	508G									
KJ676779	ET154	P1-152-246-508-16239-@212-@16176 ("P1e"*#)	16239T	16266T	16357C	73G	152C	246C	263G	315.1C	508G									
KJ676785	ET284	P1-152-246-508-16239-@212-@16176 ("P1e"*#)	16239T	16266T	16357C	73G	152C	246C	263G	315.1C	508G									
KJ676775	ET232	P1-152-246-508-16239-@212-@16176 ("P1e"*#)	16239T	16266T	16357C	73G	152C	246C	263G	315.1C	508G									
KJ676789	ET072	P1-152-246-508-16239-@212-@16176 ("P1e"*#)	16239T	16266T	16357C	73G	152C	246C	263G	315.1C	508G									
KJ655820	ET260	P1-152-246-508-16239-@212-@16176	16239T	16266T	16357C	73G	152C	185T	246C	263G	315.1C	508G								
KJ655640	ET252	P1-152-246-508-16239-@212-@16176	16239T	16266T	16357C	73G	152C	246C	263G	315.1C	508G									
KJ676783	ET300	R-16093-16311-16357 (P1d*)	16093C	16311C	16357C	73G	263G	315.1C												
KJ676786	ET156	R-16093-16311-16357 (P1d*)	16093C	16311C	16357C	73G	150T	263G	315.1C											
KJ655677	ET100	Q1	16129A	16144C	16148T	16223T	16241G	16265C	16311C	16343G	73G	89C	92A	146C	263G	309.1C	315.1C	489C				
KJ655661	ET146	Q1	16129A	16144C	16148T	16223T	16241G	16265C	16311C	16343G	73G	89C	92A	146C	263G	309.1C	315.1C	489C				
KJ655613	ET208	Q1@146	16129A	16144C	16148T	16223T	16241G	16265C	16311C	16343G	73G	89C	92A	263G	315.1C	489C						
KJ655869	ET175	Q1@146	16129A	16144C	16148T	16223T	16241G	16265C	16311C	16343G	73G	89C	92A	263G	315.1C	489C						
KJ655649	ET143	Q1@92-@146	16129A	16144C	16148T	16223T	16241G	16265C	16311C	16343G	16445C	73G	89C	263G	315.1C	489C						
KJ655629	ET318	Q1@89-@92	16129A	16144C	16148T	16223T	16241G	16265C	16311C	16343G	73G	146C	263G	309.1C	315.1C	489C	523DEL	524DEL				
KJ655604	ET020	Q1@89-@92	16129A	16144C	16148T	16223T	16241G	16265C	16311C	16343G	73G	146C	263G	309.1C	315.1C	489C	523DEL	524DEL				

KJ655605	ET044	Q1@89-@92	16129A	16144C	16148T	16223T	16241G	16265C	16311C	16343G	73G	146C	263G	315.1C	489C	523DEL	524DEL					
KJ655658	ET023	Q1-16172	16066G	16129A	16144C	16148T	16172C	16223T	16241G	16265C	16300G	16311C	16343G	73G	89C	92A	146C	263G	315.1C	489C	523DEL	524DEL
KJ655749	ET293	Q1-16172	16129A	16144C	16148T	16172C	16173T	16223T	16241G	16265C	16288C	16311C	16343G	73G	89C	92A	146C	263G	315.1C	489C		
KJ655676	ET088	Q1-16172	16129A	16144C	16148T	16172C	16223T	16241G	16265C	16288C	16311C	16343G	73G	89C	92A	146C	263G	315.1C	489C			
KJ655811	ET234	Q1-16172	16129A	16144C	16148T	16172C	16223T	16241G	16265C	16311C	16343G	73G	89C	92A	146C	263G	315.1C	489C				
KJ655791	ET081	Q1-16172 -@92	16129A	16144C	16148T	16172C	16223T	16241G	16265C	16311C	16343G	73G	89C	146C	263G	271T	309.1C	315.1C	489C			
KJ655864	ET231	Q1-208-16222-@16223	16129A	16144C	16148T	16195Y	16222T	16241G	16265C	16311C	16343G	73G	89C	92A	93G	146C	208C	263G	315.1C	489C	523DEL	524DEL
KJ655682	ET010	Q1-208-16222-@92-@16223	16111T	16129A	16144C	16148T	16222T	16241G	16265C	16311C	16343G	73G	89C	146C	208C	263G	309.1C	315.1C	489C			
KJ655657	ET153	Q1-208-16222-@92-@16223	16129A	16144C	16148T	16222T	16241G	16265C	16311C	16343G	73G	89C	146C	208C	263G	309.1C	315.1C	489C				
KJ655696	ET200	Q1-208-16222-16343T-@92-@16223	16129A	16144C	16148T	16222T	16241G	16265C	16311C	16343T	73G	89C	146C	208C	263G	309.1C	315.1C	489C				
KJ655654	ET024	Q1-208-16222-16343T-@92-@16223	16129A	16144C	16148T	16222T	16241G	16265C	16311C	16343T	73G	89C	146C	208C	263G	309.1C	315.1C	489C				
KJ655695	ET166	Q1-208-16222-16343T-@92-@16223	16129A	16144C	16148T	16222T	16241G	16265C	16311C	16343T	73G	89C	146C	194T	208C	263G	309.1C	309.2C	315.1C	489C		
KJ655751	ET289	Q1-208-16222-16343T-@92-@16223	16129A	16144C	16148T	16222T	16241G	16265C	16311C	16343T	73G	89C	146C	208C	263G	309.1C	315.1C	489C				
KJ655705	ET054	Q1-208-16222-16343T-@92-@16223	16129A	16144C	16148T	16222T	16241G	16265C	16311C	16343T	73G	89C	146C	208C	263G	309.1C	315.1C	489C				
KJ655711	ET130	Q1-208-16222-16343T-@92-@16223	16129A	16144C	16148T	16222T	16241G	16265C	16311C	16343T	73G	89C	146C	208C	263G	315.1C	489C					
KJ655724	ET273	Q1-208-16222-16343T-@92-@16223	16129A	16144C	16148T	16222T	16241G	16265C	16311C	16343T	73G	89C	146C	208C	263G	309.1C	315.1C	489C				
KJ655750	ET243	Q1-208-16222-16343T-@92-@16223	16129A	16144C	16148T	16222T	16241G	16265C	16311C	16343T	16355T	73G	89C	146C	208C	263G	309.1C	315.1C	489C			
KJ655740	ET207	Q1-208-16222-16343T-@92-@16223	16129A	16144C	16148T	16222T	16241G	16265C	16311C	16343T	73G	89C	146C	208C	263G	309.1C	315.1C	489C				
KJ655794	ET217	Q1-208-16222-16343T-@92-@16223-@16311	16129A	16144C	16148T	16222T	16241G	16265C	16343T	73G	89C	146C	208C	263G	309.1C	315.1C	489C					
KJ655719	ET242	Q1-208-16222-16343T-@92-@16223	16129A	16144C	16148T	16222T	16241G	16265C	16311C	16343T	16465Y	73G	89C	146C	208C	215R	263G	309.1C	315.1C	489C		
KJ655748	ET238	Q1-208-16222-16343T-@92-@16223	16129A	16144C	16148T	16222T	16241G	16265C	16311C	16343T	73G	89C	146C	208C	263G	309.1C	315.1C	489C				
KJ655797	ET119	Q1-208-16222-16343T-@92-@16223	16129A	16144C	16148T	16222T	16241G	16265C	16311C	16343T	73G	89C	146C	208C	263G	309.1C	315.1C	489C				
KJ655693	ET267	Q1-208-16222-16343T-@92-@16223	16129A	16144C	16148T	16222T	16241G	16265C	16311C	16343T	73G	89C	146C	208C	215R	263G	309.1C	315.1C	489C			
KJ655732	ET009	Q1-208-16222-16343T-@92-@16223	16129A	16144C	16148T	16183R	16222T	16241G	16265C	16311C	16343T	73G	89C	146C	208C	263G	309.1C	315.1C	489C			
KJ655672	ET286	Q1-208-16222-16343T-@92-@16223	16129A	16144C	16148T	16222T	16241G	16265C	16311C	16343T	73G	89C	146C	208C	263G	309.1C	315.1C	489C				
KJ655591	ET210	Q1-208-16222-16343T-@92-@16223	16129A	16144C	16148T	16222T	16241G	16265C	16311C	16343T	73G	89C	146C	208C	263G	309.1C	315.1C	489C				
KJ655808	ET204	Q1-208-16222-16343T-@92-@16223	16129A	16144C	16148T	16222T	16241G	16265C	16311C	16343T	73G	89C	146C	208C	263G	309.1C	315.1C	489C				
KJ655798	ET276	Q1-194-208-16222-16343T-@92-@16223	16129A	16144C	16148T	16189C	16193.1C	16222T	16241G	16265C	16311C	16343T	73G	89C	146C	194T	208C	263G	309.1C	309.2C	315.1C	489C
KJ655700	ET311	Q1-194-208-16222-16343T-@92-@16223	16129A	16144C	16148T	16222T	16241G	16265C	16311C	16343T	73G	89C	146C	194T	208C	263G	309.1C	309.2C	315.1C	489C		
KJ655702	ET027	Q1-194-208-16222-16343T-@92-@16223	16129A	16144C	16148T	16222T	16241G	16265C	16311C	16343T	73G	89C	146C	194T	208C	263G	309.1C	315.1C	489C			
KJ655824	ET309	Q1-194-208-16222-16343T-@92-@16223	16129A	16144C	16148T	16222T	16241G	16265C	16311C	16343T	73G	89C	146C	194T	208C	263G	309.1C	315.1C	489C			
KJ655744	ET235	Q1-194-208-16222-16343T-@92-@16223	16129A	16144C	16148T	16222T	16241G	16265C	16311C	16343T	73G	89C	146C	194T	208C	263G	309.1C	309.2C	315.1C	489C		
KJ655592	ET249	Q1-194-208-16222-16343T-@92-@16223	16129A	16144C	16148T	16222T	16241G	16265C	16311C	16343T	73G	89C	146C	194T	195Y	208C	263G	309.1C	309.2C	315.1C	489C	
KJ655780	ET037	Q1-173-238-16362-@92-@16223	16129A	16144C	16148T	16241G	16265C	16311C	16343G	16362C	73G	89C	146C	173C	238G	257G	263G	315.1C	489C			
KJ655775	ET197	Q1-173-238-16362-@92-@16129-@16223	16144C	16148T	16241G	16265C	16311C	16343G	16362C	73G	89C	146C	173C	238G	263G	315.1C	489C					
KJ655759	ET034	Q1-173-238-16362-@92	16129A	16144C	16148T	16223T	16241G	16265C	16311C	16343G	16362C	73G	89C	146C	173C	238G	263G	315.1C	489C			
KJ655701	ET174	Q1-195-285-16157-16278-@92	16129A	16144C	16148T	16156A	16157C	16223T	16241G	16265C	16278T	16311C	16343G	73G	89C	146C	195C	263G	285T	309.1C	315.1C	489C
KJ655812	ET245	Q1-195-285-16157-16278-@92	16129A	16144C	16148T	16157C	16223T	16241G	16265C	16278T	16311C	16343G	73G	89C	146C	195C	263G	285T	315.1C	489C		
KJ655660	ET251	Q1-195-285-16157-16278-@92	16129A	16144C	16148T	16157C	16223T	16241G	16265C	16278T	16311C	16343G	73G	89C	146C	195C	263G	285T	309.1C	315.1C	489C	
KJ655692	ET277	R14-56.1A-65DEL-189-207-@182-@16187-@16362	16193T	16288C	16304G	56T	56.1A	65DEL	73G	189G	204C	207A	234G	263G	309.1C	315.1C	573.1C	573.2C				
KJ655694	ET086	R14-56.1A-65DEL-189-207-@182-@16187-@16362	16093C	16288C	16304G	56T	56.1A	65DEL	73G	189G	204C	207A	234G	263G	309.1C	315.1C	573.1C	573.2C	573.3C	573.4C	573.5C	
KJ655713	ET177	R-16093-16311-16357	16093C	16311C	16357C	73G	263G	315.1C														
KJ655752	ET290	R-16093-16311-16357	16093C	16311C	16357C	16526A	73G	263G	315.1C													
KJ655754	ET173	R9c1-573.XC	16092C	16157C	16264T	16304C	73G	263G	315.1C	523DEL	524DEL	573.1C	573.2C	573.3C	573.4C	573.5C						
KJ655889	ET186	R9c1-573.XC	16157C	16304C	73G	263G	309.1C	315.1C	316A	573.1C	573.2C											
KJ676778	ET095	R9c1-151-573.XC (R9c1b2*)	16157C	16304C	73G	151T	263G	315.1C	573.1C	573.2C	573.3C											
KJ655888	ET170	R9c1-151-573.XC	16157C	16304C	73G	151T	263G	315.1C	573.1C	573.2C												
KJ655801	ET132	R9c1a	16157C	16256T	16304C	16335G	73G	249DEL	263G	315.1C												
KJ655777	ET279	R9c1a	16157C	16256T	16304C	16335G	73G	249DEL	263G	315.1C												
KJ655781	ET097	R9c1a	16157C	16256T	16304C	16335G	73G	249DEL	263G	315.1C												
KJ655667	ET152	R9c1a	16157C	16256T	16304C	16335G	73G	249DEL	263G	315.1C												
KJ655839	ET105	R9c1a	16157C	16256T	16304C	16335G	73G	249DEL	263G	315.1C												
KJ655830	ET233	R9c1a	16157C	16256T	16304C	16335G	73G	249DEL	263G	309.1C	315.1C											
KJ655643	ET282	R9c1a	16157C	16256T	16304C	16335G	73G	249DEL	263G	315.1C												
KJ655642	ET275	R9c1a	16157C	16256T	16304C	16335G	73G	249DEL	263G	315.1C	573.1C											
KJ655644	ET305	R9c1a	16157C	16256T	16304C	16335G	73G	249DEL	263G	315.1C												
KJ655841	ET158	R9c1a	16157C	16256T	16304C	16335G	73G	249DEL	263G	315.1C												
KJ655810	ET229	R9c1a	16157C	16256T	16304C	16335G	73G	249DEL	263G	315.1C												
KJ655628	ET264	R9c1a@16157	16192T	16256T	16304C	16335G	73G	249DEL	263G	315.1C												
KJ655722	ET271	R9c1a	16157C	16256T	16304C	16335G	73G	195Y	249DEL	263G	315.1C											
KJ655715	ET188	R9c1a-16189-16357	16157C	16189C	16193.1(	16193.2C	16256T	16304C	16335G	16357C	73G	249DEL	263G	297G	309.1C	309.2C	315.1C					
KJ655886	ET138	R9c1a-16189-16357	16157C	16183C	16189C	16193.1C	16256T	16304C	16335G	16357C	73G	249DEL	263G	315.1C								

*classified after complete mitogenome sequencing (cf. Additional file 4)
postulated novel clade
