# Supplementary material for: Human settlement history between Sunda and Sahul: a focus on East Timor (Timor-Leste) and the Pleistocenic mtDNA diversity
Source: BMC Genomics. 2015 Feb 14;16(1):70. doi: 10.1186/s12864-014-1201-x (PMC4342813; doi:10.1186/s12864-014-1201-x)
Supplement: Additional file 4: — Mutational profiles, haplogroups and GenBank accession codes of the 17 novel complete East Timor mitogenomes. Haplogroups according to [49], build 16. Differences are relative to the rCRS [50]. Bases are indicated according to the IUBMB nucleotide code. [file 12864_2014_1201_MOESM4_ESM.pdf]

Additional file 4: Mutational profiles, haplogroups and GenBank accession codes of the 17 novel complete East Timor mitogenomes

GenBank accession	sample	haplogroup	
KJ676776	ET005	"P1e"#	16239T 16266T 16357C 73G 152C 246C 263G 315.1C 508G 750G 1438G 2706G 3229.1A 4659A 4769G 5964C 6077T 7028T 8286C 8860G 10118C 10398T 11719A 13098G 13722G 14766T 15258G 15326G 15607G
KJ676787	ET014	P1d	16176T 16266T 16357C 16526A 73G 212C 263G 315.1C 750G 1438G 2706G 3747T 3834A 4769G 6077T 7028T 8860G 9605T 10118C 11626C 11719A 12346T 14569A 14766T 14971C 15326G 15607G
KJ676781	ET017	P1d	16176T 16266T 16357C 16526A 73G 212C 263G 315.1C 524.1A 524.2C 750G 1438G 2706G 3747T 3834A 4769G 6077T 7028T 8860G 9605T 10118C 11626C 11719A 12346T 14569A 14766T 14971C 15326G 15607G
KJ676777	ET064	P1d	16176T 16266T 16357C 73G 212C 263G 315.1C 750G 1438G 2706G 4769G 6077T 7028T 8490C 8519A 8860G 9922T 10118C 10128T 11383C 11719A 12346T 12372A 12711G 12714C 14152G 14766T 15326G 15479C 15607G
KJ676789	ET072	"P1e"#	16239T 16266T 16357C 73G 152C 246C 263G 315.1C 508G 750G 1438G 2706G 4659A 4769G 5964C 6077T 7028T 8286C 8860G 10118C 10398T 11719A 13098G 13722G 14766T 15258G 15326G 15607G
KJ676782	ET139	P1d	16156A 16169T 16176T 16189Y 16266T 16311C 16357C 73G 152C 212C 235G 263G 315.1C 750G 1406C 1438G 2706G 2857C 4533A 4688C 4769G 6018A 6077T 7028T 8251A 8860G 8939C 9150G 9269T 10118C 11719A 12346T 12561A 14766T 15326G 15607G 15938T
KJ676779	ET154	"P1e"#	16239T 16266T 16357C 73G 152C 246C 263G 315.1C 508G 750G 1438G 2706G 3229.1A 4659A 4769G 5964C 6077T 7028T 8286C 8860G 10118C 10398T 11719A 13098G 13722G 14766T 15258G 15326G 15607G
KJ676786	ET156	P1d	16093C 16311C 16357C 73G 150T 263G 315.1C 750G 1438G 2706G 3474T 4769G 5444T 6077T 6452A 7028T 7859A 8860G 9051G 10118C 11719A 12346T 14383A 14766T 15326G 15607G
KJ676784	ET167	P1d	16156A 16169T 16176T 16266T 16311C 16357C 73G 152C 212C 235G 263G 315.1C 524.1A 524.2C 750G 1438G 2706G 2857C 4533A 4688C 4769G 6018A 6077T 7028T 8251A 8860G 8939C 9150G 9269T 10118C 11719A 12346T 12561A 14766T 15038G 15326G 15607G 15938T
KJ676775	ET232	"P1e"#	16239T 16266T 16357C 73G 152C 246C 263G 315.1C 508G 750G 1438G 2706G 4659A 4769G 5964C 6077T 7028T 8286C 8287.1C 8287.2C 8287.3C 8860G 10118C 10398T 11719A 13098G 13722G 14766T 15258G 15326G 15607G
KJ676785	ET284	"P1e"#	16239T 16266T 16357C 73G 152C 246C 263G 315.1C 508G 750G 1438G 2706G 4659A 4769G 5964C 6077T 7028T 8286C 8287.1C 8287.2C 8287.3C 8860G 10118C 10398T 11719A 13098G 13722G 14766T 15258G 15326G 15607G
KJ676783	ET300	P1d	16093C 16311C 16357C 73G 263G 315.1C 750G 1438G 1676G 2706G 3474T 4769G 5444T 6077T 6452A 7028T 7859A 8860G 9051G 10118C 11719A 12346T 14383A 14766T 15326G 15607G
KJ676790	ET110	M21b	16093C 16223T 16311C 16519C 73G 152C 174T 263G 299DEL 315.1C 489C 750G 1438G 2706G 3915A 4491A 4769G 4913G 5108C 6286C 6359G 6662G 7028T 7861C 8701G 8860G 9540C 10398G 10400T 10873C 11482C 11560G 11719A 12705T 14766T 14783C 15043A 15301A 15326G
KJ676774	ET126	D6a	16192T 16223T 16274A 16362C 73G 263G 309.1C 315.1C 489C 709A 750G 1438G 1719A 2706G 3714G 4769G 4883T 5178A 7028T 8473Y 8701G 8860G 9540C 10398G 10400T 10873C 11017C 11719A 12654G 12705T 14551G 14766T 14783C 15043A 15301A 15326G
KJ676788	ET215	M73a	16184A 16192T 16223T 16239T 16245Y 16278T 16354T 73G 263G 309.1C 315.1C 489C 750G 1438G 2263A 2352C 2706G 3396C 3399T 4227G 4769G 4856C 5655C 7028T 8701G 8860G 9540C 10398G 10400T 10685A 10873C 11476T 11719A 11971T 12705T 14034C 14502C 14766T 14783C 15043A 15301A 15326G
KJ676780	ET257	Q3	16129A 16223T 16311C 16319A 73G 146C 195C 215G 263G 309.1C 315.1C 489C 519G 750G 1438G 2706G 4117C 4335T 4769G 4924A 4928C 5483C 5843G 7028T 8200C 8701G 8790A 8860G 9540C 9656C 9833C 10398G 10400T 10873C 11719A 12705T 12930G 12940A 13500C 14766T 14783C 15043A 15172A 15301A 15326G
KJ676778	ET095	R9c1b2	16157C 16304C 73G 151T 263G 315.1C 573.1C 573.2C 573.3C 750G 1438G 2706G 3645C 3970T 4769G 7028T 7299G 7861C 8860G 10163A 11002G 11719A 12406A 12618A 12858T 13928C 14766T 15326G 15479C

postulated novel clade
