# Supplementary material for: Human settlement history between Sunda and Sahul: a focus on East Timor (Timor-Leste) and the Pleistocenic mtDNA diversity
Source: BMC Genomics. 2015 Feb 14;16(1):70. doi: 10.1186/s12864-014-1201-x (PMC4342813; doi:10.1186/s12864-014-1201-x)
Supplement: Additional file 5: — List of the complete haplogroup P1 mitogenomes depicted in Figure 3 . [file 12864_2014_1201_MOESM5_ESM.pdf]

Additional file 5: List of the complete haplogroup P1 mitogenomes depicted in Figure 3

Sample ID in Figure 3	GenBank accession	Geographic origin	Reference	Haplogroup	Sample ID in this study
1	KJ676787	East Timor	this study	**P1d**	ET014
2	KJ676781	East Timor	this study	**P1d**	ET017
3	KJ676784	East Timor	this study	**P1d**	ET167
4	KJ676782	East Timor	this study	**P1d**	ET139
5	KJ676786	East Timor	this study	**P1d**	ET156
6	KJ676783	East Timor	this study	**P1d**	ET300
7	AY289092	Papua New Guinea (Highland)	[53]	**P1d**	--
8	KJ676777	East Timor	this study	**P1d**	ET064
9	EU597507	Papua New Guinea (Marcus Feldman, pers. comm.)	[54]	**P1d1**	--
10	KC994150	Philippines	[55]	**P1d1**	--
11	AF347005	Papua New Guinea (Highland)	[52]	**P1d1**	--
12	AY289087	Papua New Guinea (Highland)	[53]	**P1d1**	--
13	AF347004	Papua New Guinea (Highland)	[52]	**"P1e"#**	--
14	KJ676789	East Timor	this study	**"P1e"#**	ET072
15	KJ676775	East Timor	this study	**"P1e"#**	ET232
16	KJ676785	East Timor	this study	**"P1e"#**	ET284
17	KJ676776	East Timor	this study	**"P1e"#**	ET005
18	KJ676779	East Timor	this study	**"P1e"#**	ET154
19	AF347002	Papua New Guinea (Coast)	[52]	**P1**	--
20	AY289086	Papua New Guinea (Highland)	[53]	**P1**	--

Haplogroups according to Phylotree [49], build 16

postulated novel clade
