# Supplementary material for: Human settlement history between Sunda and Sahul: a focus on East Timor (Timor-Leste) and the Pleistocenic mtDNA diversity
Source: BMC Genomics. 2015 Feb 14;16(1):70. doi: 10.1186/s12864-014-1201-x (PMC4342813; doi:10.1186/s12864-014-1201-x)
Supplement: Additional file 6: — Novel non-P1 complete mitogenomes from East Timor. The five East Timor samples are shown in the context of published sequences. Color codes indicate the country of origin. All identified differences are relative to the rCRS [50], disregarding cytosine insertions after nps 16193 and 309. Haplogroups according to [49], build 16. Bases are indicated according to the IUBMB nucleotide code. The prefix @ indicates the reversion of a mutation occurring earlier in the phylogeny. Underlined mutations are recurrent in the tree. References and GenBank accession numbers are indicated for each sample. Samples marked with an asterisk are available from GenBank only [Pradutkanchana 2010, unpublished data]. [file 12864_2014_1201_MOESM6_ESM.pdf]

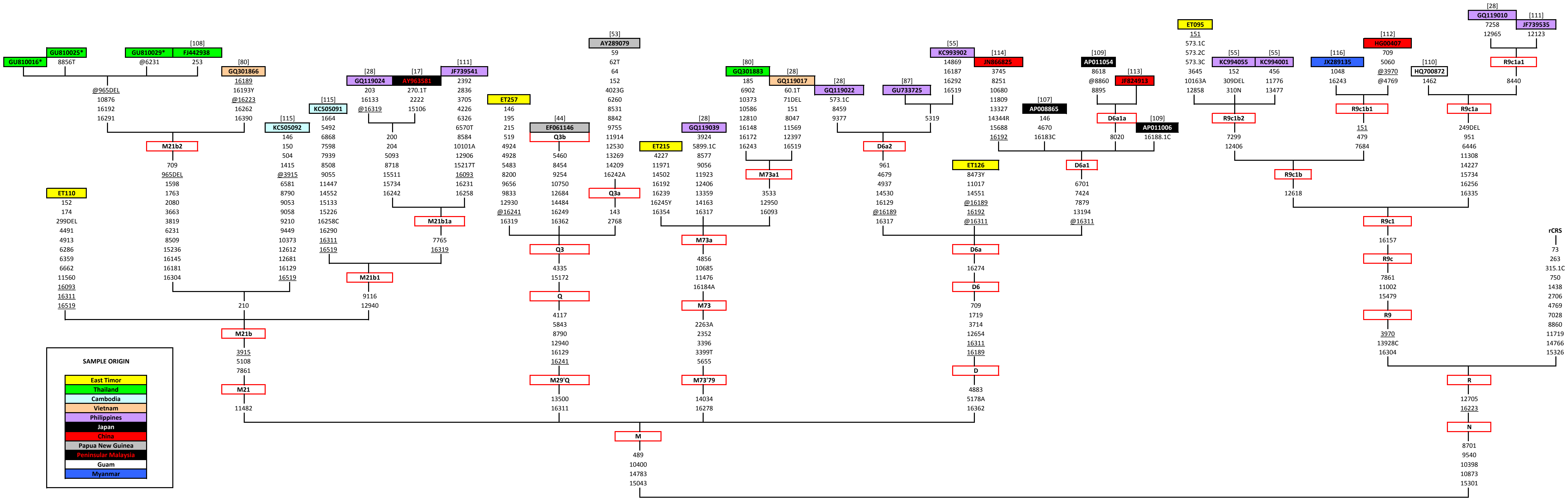
SAMPLE ORIGIN
East Timor
Thailand
Cambodia
Vietnam
Philippines
Japan
China
Papua New Guinea
Peninsular Malaysia
Guam
Myanmar
GU810025*
GU810016*
GU810029*
FJ442938
GQ301866
KC505091
KC505092
GQ119024
AY963581
JF739541
ET257
EF061146
AY289079
ET215
GQ119039
GQ301883
GQ119017
GQ119022
GU733725
KC993902
JN866825
AP011054
AP008865
JF824913
AP011006
ET126
ET095
KC994055
KC994001
JX289135
HQ700872
GQ119010
JF739535
ET110
M21b2
M21b1a
M21b1
M21b
M21
Q3b
Q3a
Q3
Q
M29'Q
M73a1
M73a
M73
M73'79
D6a2
D6a1a
D6a1
D6a
D6
D
M
R9c1a1
R9c1a
R9c1b1
R9c1b2
R9c1b
R9c1
R9c
R9
R
N
rCRS
