# Supplementary material for: Human settlement history between Sunda and Sahul: a focus on East Timor (Timor-Leste) and the Pleistocenic mtDNA diversity
Source: BMC Genomics. 2015 Feb 14;16(1):70. doi: 10.1186/s12864-014-1201-x (PMC4342813; doi:10.1186/s12864-014-1201-x)
Supplement: Additional file 7: — Shared haplotypes between East Timor and 10 surrounding populations based on HVS-I and HVS-II. [file 12864_2014_1201_MOESM7_ESM.pdf]

Additional File 7: Shared haplotypes between East Timor and 10 surrounding populations based on HVS-I and HVS-II

**Population**	**1A - East Timor**	**8 - Penins.Malaysia (Singapore)**	**12 - Philippines**	**13 - Vietnam**	**14 - Laos**	**15 - Thailand**	**16 - South Korea**	**17 - Taiwan**	**18 - Hainan**	**19 - Mixed Han (China)**	**21 - PNG**
**Reference**	this study	[57]	[28]	[58]	[59]	[60]	[61]	[45]	[62]	[63]	[34,64]
**number of samples**	324	205	130	187	214	190	692	640	290	262	142
**number of haplotypes**	**145**	**152**	**83**	**153**	**163**	**133**	**471**	**122**	**137**	**242**	**85**
ET103	4	0	0	0	1	0	0	1	0	0	0
ET278	2	1	0	0	2	2	0	0	0	0	0
ET017	3	0	0	0	0	0	0	0	0	0	2
ET165	10	0	0	0	0	0	0	0	0	0	1
ET218	15	1	2	1	0	0	0	3	3	0	0
ET187	4	0	2	0	1	0	1	1	4	0	0
ET131	11	1	1	0	0	0	2	2	0	0	0
ET147	1	1	1	0	0	0	22	11	0	1	0
ET219	2	1	1	0	0	0	0	16	3	0	0
ET030	10	1	7	0	0	0	0	14	0	0	0
ET067	13	1	9	0	0	0	0	19	0	0	0
ET161	9	0	6	0	0	0	0	21	0	0	0
ET152	11	0	2	0	0	0	0	14	0	0	0
ET012	14	0	3	0	0	0	0	0	0	0	0
ET084	6	1	2	0	0	0	0	12	0	0	0
ET099	2	1	2	0	0	0	0	0	0	0	0
ET206	1	0	2	0	0	0	0	0	0	0	0
ET092	2	1	1	0	0	0	0	0	0	0	0
ET181	1	0	2	0	0	0	0	17	0	0	0
ET314	1	0	0	0	0	0	0	7	0	0	0
ET061	4	0	0	0	0	0	0	1	0	0	0
ET140	2	0	0	0	0	0	0	5	0	0	0
ET079	1	0	0	0	0	0	0	3	0	0	0
ET299	6	1	0	0	0	0	0	0	0	0	0
ET182	1	1	0	0	0	0	0	0	0	0	0
ET196	1	1	0	0	0	0	0	0	0	0	0
ET015	1	0	0	0	0	1	0	0	0	0	0
**number of shared haplotypes**	**27**	**13**	**15**	**1**	**3**	**2**	**3**	**16**	**3**	**1**	**2**
**proportion of shared haplotypes [%]**	**18,62**	**8,55**	**18,07**	**0,65**	**1,84**	**1,50**	**0,64**	**13,11**	**2,20**	**0,42**	**2,35**
**individuals with shared haplotypes**	**138**	**13**	**43**	**1**	**4**	**3**	**25**	**147**	**10**	**1**	**3**
**proportion of individuals with shared haplotypes [%]**	**42,59**	**6,34**	**33,08**	**0,53**	**1,87**	**1,58**	**3,61**	**22,97**	**3,45**	**0,38**	**2,11**

reading frame: nps 16080-16193 16194-16365 73-300
one example haplotype given per shared haplotype
highest shared proportion marked in green
lowest shared proportion marked in red
