# Supplementary material for: Human settlement history between Sunda and Sahul: a focus on East Timor (Timor-Leste) and the Pleistocenic mtDNA diversity
Source: BMC Genomics. 2015 Feb 14;16(1):70. doi: 10.1186/s12864-014-1201-x (PMC4342813; doi:10.1186/s12864-014-1201-x)
Supplement: Additional file 8: — Intra-population comparisons for East Timor and 10 surrounding populations based on HVS-I and HVS-II. Part A: Design and results of AMOVA. Part B: Mean pairwise differences. Part C: F ST comparisons. [file 12864_2014_1201_MOESM8_ESM.pdf]

Additional File 8: Intra-population comparisons for East Timor and 10 surrounding populations based on HVS-I and HVS-II

reading frame: nps 16080-16193 16194-16365 73-300

Part A: Design and results of AMOVA

Source of variation	d.f.	Sum of squares	Variance components	Percentage of variation
Among populations	10	783,765	0.25597 Va	4,86
Within populations	3265	16350,495	5.00781 Vb	95,14
Total	3275	17134,26	5,26378	

p-value (Va): < 0.001

Part B: Mean pairwise differences

Population	East Timor	PNG	Vietnam	South Korea	Hainan	Philippines	Taiwan	Peninsular Malaysia	Mixed Han (China)	Thailand	Laos
1A - East Timor	10,70	**12,78**	10,90	10,54	10,96	**10,15**	10,59	10,87	11,01	10,87	10,96
21 - PNG	**1,48**	11,88	13,36	13,05	13,38	12,62	13,22	13,32	13,52	13,32	13,42
13 - Vietnam	0,50	2,37	10,10	10,10	10,31	9,93	10,22	10,34	10,47	10,08	10,14
16 - South Korea	0,50	2,42	0,36	9,37	10,20	9,45	10,03	10,03	9,96	10,02	10,18
18 - Hainan	0,45	2,28	0,10	0,36	10,31	9,97	10,30	10,47	10,58	10,29	10,35
12 - Philippines	**0,26**	2,14	0,34	0,23	0,27	9,08	9,59	9,85	9,98	9,90	9,99
17 - Taiwan	0,41	2,46	0,35	0,53	0,32	0,23	9,64	10,35	10,45	10,19	10,24
8 - Peninsular Malaysia (Singapore)	0,34	2,20	0,11	0,17	0,14	0,14	0,35	10,35	10,49	10,32	10,41
19 - Mixed Han (China)	0,42	2,34	0,18	0,04	0,19	0,20	0,39	0,08	10,48	10,40	10,56
15 - Thailand	0,54	2,40	0,05	0,35	0,15	0,38	0,39	0,16	0,18	9,96	10,14
14 - Laos	0,51	2,39	0,00	0,41	0,10	0,36	0,33	0,14	0,23	0,06	10,18

Values above diagonal: Average number of pairwise differences between populations (PiXY)
Diagonal elements: Average number of pairwise differences within populations (PiX)
Values below diagonal: Corrected number of average pairwise differences between populations (PiXY-(PiX+PiY)/2)
Lowest and highest values are highlighted in green and red, resp.
Values concerning the East Timor sample are highlighted in grey (lows and highs are bold)

Part C: FST comparisons

Population	East Timor	PNG	Vietnam	South Korea	Hainan	Philippines	Taiwan	Peninsular Malaysia	Mixed Han (China)	Thailand	Laos
1A - East Timor	*	**0,11835**	0,04524	0,04841	0,04085	**0,02423**	0,03968	0,03128	0,03809	0,04878	0,04656
21 - PNG	< 0.001	*	0,17886	0,19885	0,17412	0,16866	0,19714	0,16719	0,17594	0,18216	0,18038
13 - Vietnam	< 0.001	< 0.001	*	0,0367	0,00971	0,03366	0,03479	0,01032	0,01728	0,00474	-0,00009
16 - South Korea	< 0.001	< 0.001	< 0.001	*	0,03598	0,02347	0,05243	0,01768	0,00412	0,03575	0,04086
18 - Hainan	< 0.001	< 0.001	< 0.001	< 0.001	*	0,02606	0,03125	0,013	0,01774	0,01416	0,00928
12 - Philippines	< 0.001	< 0.001	< 0.001	< 0.001	< 0.001	*	0,02362	0,01359	0,01975	0,03753	0,03563
17 - Taiwan	< 0.001	< 0.001	< 0.001	< 0.001	< 0.001	< 0.001	*	0,03501	0,03773	0,03861	0,03225
8 - Peninsular Malaysia (Singapore)	< 0.001	< 0.001	< 0.001	< 0.001	< 0.001	< 0.001	< 0.001	*	0,00733	0,01584	0,01373
19 - Mixed Han (China)	< 0.001	< 0.001	< 0.001	< 0.001	< 0.001	< 0.001	< 0.001	< 0.001	*	0,01696	0,02131
15 - Thailand	< 0.001	< 0.001	0,00901	< 0.001	< 0.001	< 0.001	< 0.001	< 0.001	< 0.001	*	0,00612
14 - Laos	< 0.001	< 0.001	0,36036	< 0.001	0,00901	< 0.001	< 0.001	< 0.001	< 0.001	< 0.001	*

Values above diagonal: population pairwise F_{ST} values
Lowest and highest values are highlighted in green and red, resp.
Values below diagonal: p-values (significance level=0,05; 1023 permutations)
East Timor values are highlighted in grey, maximum and minimum vales bold
p value above 0.05 is highlighted in yellow

References for populations: see Table 2
