# Supplementary material for: Human settlement history between Sunda and Sahul: a focus on East Timor (Timor-Leste) and the Pleistocenic mtDNA diversity
Source: BMC Genomics. 2015 Feb 14;16(1):70. doi: 10.1186/s12864-014-1201-x (PMC4342813; doi:10.1186/s12864-014-1201-x)
Supplement: Additional file 9: — Shared haplotypes between East Timor and 24 surrounding populations based on HVS-I. [file 12864_2014_1201_MOESM9_ESM.pdf]

Additional file 9: Shared Haplotypes between East Timor and 16 surrounding populations based on HVS-I data
(reading frame: 16080-16180 16195-16354)

Population	East Timor	Nusa Tenggara	Bali	Java	Sumatra	Mentawai	Nias	Peninsular Malaysia	Borneo	Sulawesi	Moluccas	Philippines	Vietnam	Laos	Thailand	Hainan	Taiwan	China (Mixed Han)	Korea	WNG	PNG	Admiralty Islands	Solomons	Polynesia	Australia (Indigenous)
Reference	[3,this study]	[3,5,24,65]	[5,24]	[5,24]	[5,40]	[5]	[5,41]	[17,57]	[24]	[5,24]	[24,65]	[24,28]	[58]	[59]	[60]	[62]	[24,45]	[63]	[61]	[66]	[34,64,67]	[67,68]	[29]	[65,67]	[34,69]
**number of samples**	362	1699	570	97	228	128	499	470	157	437	74	483	187	214	190	293	718	262	692	227	201	203	703	394	146
**number of haplotypes**	**94**	**345**	**138**	**49**	**107**	**16**	**61**	**158**	**102**	**153**	**36**	**140**	**119**	**127**	**108**	**99**	**86**	**194**	**306**	**74**	**72**	**41**	**102**	**56**	**50**
ET030	39	33	14	2	6	0	1	2	10	61	3	43	0	0	0	0	24	1	1	0	0	0	0	0	0
ET067	26	82	11	6	15	7	22	11	3	32	4	37	3	1	1	9	22	3	3	0	0	2	10	1	0
ET045	24	88	0	0	2	0	0	1	3	13	0	12	1	0	0	3	6	0	0	0	0	0	0	0	0
ET012	18	53	17	2	8	0	7	3	5	22	10	23	2	6	1	1	40	10	69	2	3	0	6	0	7
ET152	15	14	2	0	1	0	0	0	3	1	0	14	0	0	0	0	14	1	0	0	0	0	0	0	0
ET165	13	7	0	0	0	0	0	0	2	9	6	0	0	0	0	0	0	0	0	0	3	70	305	225	0
ET131	12	72	11	2	2	0	0	1	2	7	8	15	1	0	0	0	2	0	2	0	0	0	0	0	0
ET100	11	15	0	0	0	0	0	0	0	2	4	0	0	0	0	0	0	0	0	31	33	27	5	1	0
ET161	11	36	3	0	13	0	36	1	7	18	3	40	0	0	0	2	41	2	3	0	0	16	77	41	0
ET202	8	56	4	0	2	4	3	2	1	16	0	3	0	0	0	0	1	0	0	0	0	2	6	0	0
ET187	7	59	0	0	1	0	0	1	1	8	2	20	0	2	0	15	47	0	2	0	0	0	3	5	0
ET319	7	19	36	2	3	0	13	2	2	22	1	15	0	0	1	1	12	0	1	0	0	0	0	0	0
ET212	6	56	5	0	2	23	21	3	2	7	1	24	0	0	0	0	0	0	5	0	0	3	1	0	0
ET103	5	1	0	0	0	0	5	1	0	0	0	0	1	1	0	0	1	1	1	0	0	0	1	2	0
ET106	5	11	3	0	0	0	0	1	1	1	1	2	0	0	0	0	0	0	0	0	0	1	2	0	0
ET065	4	0	0	0	0	0	0	0	0	0	0	0	0	0	0	0	7	0	0	0	0	0	0	0	0
ET272	4	3	0	0	0	0	0	0	0	0	0	0	0	0	0	0	0	0	0	0	0	0	0	0	0
ET017	3	3	0	0	0	0	0	0	0	0	2	0	0	0	0	0	0	0	0	8	4	2	5	0	0
ET071	3	0	10	0	0	0	0	0	0	0	0	3	1	1	0	1	0	0	0	0	0	0	0	0	0
ET074	3	33	0	0	0	0	12	1	1	8	0	1	0	0	0	0	9	0	0	0	0	0	0	0	0
ET186	3	0	0	0	0	0	0	0	0	0	0	2	0	0	0	3	0	0	0	0	0	0	0	0	0
ET234	3	10	0	0	0	0	0	0	1	0	1	0	0	0	0	0	0	0	0	13	1	0	0	0	0
ET274	3	0	0	0	0	0	0	0	0	1	0	0	0	0	0	0	0	0	0	0	0	0	0	0	0
ET008	2	1	0	0	0	0	0	0	0	0	0	0	0	0	0	0	0	0	0	0	0	0	0	0	0
ET021	2	54	5	4	5	0	1	3	4	5	0	3	1	2	1	3	0	3	15	0	0	0	0	0	0
ET094	2	22	0	0	1	0	0	0	0	1	0	0	0	0	0	0	0	0	0	0	0	0	0	0	0
ET153	2	1	0	0	0	0	0	0	0	0	0	0	0	0	0	0	0	0	0	7	0	0	0	0	0
ET209	2	6	0	0	0	0	0	0	0	0	0	0	0	0	0	0	0	0	0	0	0	0	0	0	0
ET278	2	5	11	1	3	0	1	3	4	3	0	0	0	3	3	0	0	0	0	0	0	0	0	0	0
FJ838905 [3]	2	10	1	6	2	0	0	3	0	1	0	1	2	1	8	8	0	3	1	0	3	0	0	0	7
ET015	1	0	0	0	0	0	0	0	0	1	0	0	0	0	1	0	0	0	1	0	0	0	0	0	0
ET037	1	16	0	0	0	0	0	0	0	0	0	0	0	0	0	0	0	0	0	1	2	0	0	0	0
ET077	1	0	0	0	1	0	0	0	0	0	0	0	0	0	0	0	0	0	0	0	0	0	0	0	0
ET078	1	26	1	5	6	0	8	24	0	3	1	0	8	7	10	3	0	2	0	0	0	0	0	0	0
ET079	1	0	0	0	0	0	0	0	0	0	0	0	0	0	1	0	3	0	0	0	0	0	0	0	0
ET110	1	1	0	4	0	0	0	0	1	0	0	2	0	0	0	1	0	0	0	0	0	0	0	0	0
ET122	1	4	0	0	0	0	0	0	0	0	0	0	0	0	0	0	0	0	0	0	0	0	0	0	0
ET126	1	15	1	0	0	0	0	1	0	3	0	0	1	0	1	0	0	1	5	0	0	0	0	0	0
ET181	1	7	0	0	0	0	5	0	0	0	0	3	0	0	0	0	19	0	0	0	0	0	0	0	0
ET194	1	0	0	0	0	0	1	0	0	0	0	1	0	0	0	0	0	0	0	0	0	0	0	0	0
ET196	1	9	0	0	0	0	0	1	0	1	0	0	0	0	0	0	0	0	0	0	0	0	0	0	0
ET224	1	0	0	0	0	0	0	0	0	0	0	2	0	0	0	0	0	0	0	0	0	0	0	0	0
ET225	1	0	0	0	0	0	0	0	0	0	0	0	0	0	0	0	0	0	0	0	0	3	5	9	0
ET257	1	7	0	0	0	0	0	0	0	0	0	0	0	0	0	0	0	0	0	0	0	0	0	0	0
ET259	1	5	0	0	0	0	0	0	0	0	0	0	0	0	0	0	0	0	0	0	0	0	0	0	0
ET264	1	0	0	0	0	0	0	0	0	0	0	0	0	0	0	0	1	0	0	0	0	0	0	0	0
ET277	1	1	1	0	0	0	0	0	0	1	0	0	0	0	0	0	0	0	0	0	1	0	0	0	0
ET283	1	0	0	0	0	0	0	0	0	0	1	0	0	0	0	0	0	0	0	0	0	0	0	0	0
FJ838923 [3]	1	1	0	0	0	0	0	0	0	0	0	0	0	0	0	0	0	0	0	0	0	0	0	0	0
FJ838931 [3]	1	0	0	0	1	0	0	0	0	0	0	0	0	0	0	0	0	0	0	0	0	0	0	0	0
FJ838932 [3]	1	0	0	0	0	0	0	0	0	0	0	0	0	0	0	0	0	0	1	0	0	0	0	0	0
**number of shared haplotypes**	**51**	**37**	**17**	**10**	**18**	**3**	**14**	**19**	**18**	**25**	**15**	**21**	**10**	**9**	**10**	**12**	**16**	**10**	**14**	**6**	**8**	**9**	**12**	**7**	**2**
**proportion of shared haplotypes [%]**	**54,26**	**10,72**	**12,32**	**20,41**	**16,82**	**18,75**	**22,95**	**12,03**	**17,65**	**16,34**	**41,67**	**15,00**	**8,40**	**7,09**	**9,26**	**12,12**	**18,60**	**5,15**	**4,58**	**8,11**	**11,11**	**21,95**	**11,76**	**12,50**	**4,00**
**individuals with shared haplotypes**	**268**	**842**	**136**	**34**	**74**	**34**	**136**	**65**	**53**	**247**	**48**	**266**	**21**	**24**	**28**	**50**	**249**	**27**	**110**	**62**	**50**	**126**	**426**	**284**	**14**
**proportion of individuals with shared haplotypes [%]**	**74,03**	**49,56**	**23,86**	**35,05**	**32,46**	**26,56**	**27,25**	**13,83**	**33,76**	**56,52**	**64,86**	**55,07**	**11,23**	**11,21**	**14,74**	**17,06**	**34,68**	**10,31**	**15,90**	**27,31**	**24,88**	**62,07**	**60,60**	**72,08**	**9,59**

one example haplotype given per shared haplotype
highest shared proportion marked in green
lowest shared proportion marked in red
