# Supplementary material for: Human settlement history between Sunda and Sahul: a focus on East Timor (Timor-Leste) and the Pleistocenic mtDNA diversity
Source: BMC Genomics. 2015 Feb 14;16(1):70. doi: 10.1186/s12864-014-1201-x (PMC4342813; doi:10.1186/s12864-014-1201-x)
Supplement: Additional file 10: — Intra-population comparisons for East Timor and 24 surrounding populations based on HVS-I. Part A: Design and results of AMOVA. Part B: Mean pairwise differences. Part C: F ST comparisons. [file 12864_2014_1201_MOESM10_ESM.pdf]

Additional file 10: Intra-population comparisons for East Timor and 24 surrounding populations based on HVS-I

reading frame: nps 16080-16180 16195-16354

Part A: Design and results of AMOVA

Source of variation	d.f.	Sum of squares	Variance components	Percentage of variation
Among populations	24	3385,858	0.37056 Va	12,44
Within populations	9609	25061,382	2.60812 Vb	87,56
Total	9633	28447,240	2,97868	

p-value (Va): < 0.001

Part B: Mean pairwise differences

Population	East Timor	Nusa Tenggara	Java	Bali	Philippines	Sulawesi	Borneo	Moluccas	Sumatra	Peninsular Malaysia	South Korea	Taiwan	Hainan	Mixed Han (China)	Vietnam	Laos	Thailand	WNG	PNG	Admiralty Islands	Solomons	Polynesia	Australia	Mentawai	Nias
1 - East Timor	5,98	5,96	5,96	6,08	5,70	**5,57**	5,81	5,74	5,93	6,50	5,74	5,85	5,90	6,02	5,97	6,08	6,04	**8,16**	7,11	6,41	6,08	6,00	5,65	5,86	6,07
2 - Nusa Tenggara	0,07	5,82	5,75	5,89	5,56	5,44	5,65	5,63	5,74	6,28	5,52	5,70	5,68	5,81	5,74	5,85	5,80	8,37	7,21	6,52	6,11	6,09	5,49	5,65	5,93
4 - Java	0,19	0,06	5,57	5,78	5,48	5,35	5,52	5,60	5,59	6,11	5,37	5,62	5,53	5,65	5,58	5,70	5,64	8,54	7,32	6,65	6,18	6,21	5,35	5,52	5,82
3 - Bali	0,27	0,16	0,17	5,64	5,46	5,38	5,60	5,72	5,69	6,26	5,51	5,64	5,64	5,80	5,70	5,80	5,82	9,01	7,59	6,68	6,07	6,00	5,43	5,50	5,76
12 - Philippines	0,18	0,12	0,17	0,11	5,05	5,03	5,25	5,37	5,37	5,96	5,19	5,22	5,34	5,48	5,45	5,56	5,52	8,73	7,32	6,22	5,52	5,39	5,07	5,16	5,34
10 - Sulawesi	0,18	0,13	0,16	0,15	0,10	4,80	5,12	5,22	5,27	5,80	4,95	5,26	5,24	5,29	5,33	5,45	5,42	8,41	7,12	6,12	5,48	5,44	4,86	5,16	5,49
9 - Borneo	0,15	0,07	0,07	0,11	0,05	0,04	5,35	5,45	5,47	6,02	5,20	5,43	5,43	5,51	5,53	5,63	5,59	8,61	7,31	6,36	5,74	5,68	5,14	5,33	5,61
11 - Moluccas	**0,04**	0,01	0,10	0,18	0,13	0,10	0,06	5,43	5,55	6,12	5,32	5,52	5,52	5,62	5,56	5,67	5,64	8,06	6,93	6,03	5,59	5,48	5,31	5,52	5,76
5 - Sumatra	0,18	0,07	0,05	0,11	0,08	0,11	0,03	0,08	5,52	6,13	5,32	5,54	5,52	5,62	5,60	5,71	5,67	8,70	7,37	6,51	5,93	5,90	5,26	5,36	5,57
8 - Penins. Malaysia	0,33	0,19	0,15	0,27	0,26	0,23	0,17	0,24	0,19	6,35	5,87	6,15	6,05	6,15	6,05	6,18	6,12	9,38	8,03	7,26	6,69	6,73	5,87	5,96	6,47
16 - South Korea	0,33	0,19	0,16	0,26	0,24	0,12	0,10	0,19	0,13	0,27	4,85	5,40	5,26	5,21	5,35	5,48	5,43	8,42	7,13	6,33	5,80	5,85	4,92	5,23	5,64
17 - Taiwan	0,27	0,20	0,23	0,22	0,09	0,25	0,16	0,21	0,18	0,38	0,37	5,20	5,47	5,65	5,59	5,69	5,65	8,80	7,38	6,28	5,55	5,37	5,29	5,42	5,51
18 - Hainan	0,24	0,11	0,08	0,15	0,14	0,17	0,09	0,14	0,09	0,21	0,16	0,21	5,34	5,52	5,46	5,57	5,53	8,76	7,41	6,56	6,00	5,97	5,24	5,45	5,73
19 - Mixed Han	0,27	0,13	0,10	0,21	0,18	0,12	0,07	0,14	0,09	0,21	0,02	0,28	0,08	5,53	5,60	5,73	5,67	8,74	7,44	6,64	6,10	6,12	5,26	5,54	5,90
13 - Vietnam	0,26	0,11	0,07	0,16	0,20	0,20	0,13	0,13	0,12	0,15	0,21	0,27	0,06	0,12	5,45	5,56	5,53	8,79	7,46	6,62	6,10	6,09	5,39	5,56	5,92
14 - Laos	0,25	0,10	0,07	0,14	0,19	0,20	0,11	0,12	0,10	0,16	0,21	0,24	0,05	0,12	-0,01	5,69	5,65	8,90	7,57	6,71	6,16	6,13	5,48	5,67	6,00
15 - Thailand	0,26	0,10	0,06	0,20	0,20	0,22	0,12	0,13	0,11	0,15	0,21	0,26	0,07	0,11	0,01	0,01	5,58	8,85	7,52	6,71	6,20	6,19	5,42	5,61	5,97
20 - WNG	1,65	1,94	2,23	2,66	2,68	2,48	2,42	1,83	2,41	2,68	2,47	2,68	2,56	2,45	2,55	2,54	2,53	7,04	7,19	8,28	9,30	9,59	8,52	8,73	8,85
21 - PNG	0,76	0,94	1,17	1,41	1,42	1,35	1,27	0,85	1,24	1,49	1,34	1,42	1,38	1,30	1,38	1,36	1,36	0,30	6,73	7,31	7,85	8,02	7,15	7,29	7,45
22 - Admiralty Islands	0,57	0,76	1,02	1,01	0,85	0,87	0,84	0,48	0,90	1,24	1,06	0,83	1,05	1,02	1,05	1,02	1,08	1,91	1,10	5,69	5,11	4,63	6,33	6,56	6,52
23 - Solomons	1,26	1,37	1,57	1,42	1,17	1,25	1,24	1,04	1,34	1,68	1,54	1,12	1,50	1,50	1,55	1,49	1,58	3,95	2,65	0,44	3,66	2,88	5,70	6,02	5,89
24 - Polynesia	**2,12**	2,29	2,53	2,28	1,97	2,14	2,11	1,88	2,24	2,66	2,53	1,88	2,40	2,46	2,47	2,39	2,51	5,18	3,76	0,89	0,16	1,79	5,71	6,03	5,71
25 - Australia (Indigenous)	0,39	0,31	0,28	0,33	0,27	0,18	0,19	0,32	0,22	0,42	0,22	0,42	0,29	0,22	0,39	0,36	0,35	2,72	1,50	1,21	1,59	2,54	4,56	5,12	5,46
6 - Mentawai	0,53	0,40	0,39	0,34	0,29	0,41	0,32	0,46	0,25	0,44	0,46	0,48	0,43	0,43	0,49	0,48	0,48	2,86	1,58	1,37	1,84	2,79	0,50	4,69	5,11
7 - Nias	0,70	0,64	0,65	0,55	0,43	0,71	0,55	0,66	0,43	0,91	0,83	0,53	0,68	0,75	0,81	0,77	0,79	2,95	1,70	1,29	1,67	2,43	0,80	0,38	4,77

Values above diagonal: Average number of pairwise differences between populations (PiXY)
Diagonal elements: Average number of pairwise differences within populations (PiX)
Values below diagonal: Corrected number of average pairwise differences between populations (PiXY-(PiX+PiY)/2)
Lowest and highest values are highlighted in green and red, resp.
Values concerning the East Timor sample are highlighted in grey (lows and highs are bold)

Part C: FST comparisons

Population	East Timor	Nusa Tenggara	Java	Bali	Philippines	Sulawesi	Borneo	Moluccas	Sumatra	Peninsular Malaysia	South Korea	Taiwan	Hainan	Mixed Han (China)	Vietnam	Laos	Thailand	WNG	PNG	Admiralty Islands	Solomons	Polynesia	Australia	Mentawai	Nias
1 - East Timor	*	0,011	0,031	0,044	0,033	0,033	0,026	**0,005**	0,030	0,051	0,059	0,047	0,040	0,044	0,043	0,041	0,042	0,205	0,109	0,089	0,221	**0,358**	0,065	0,085	0,117
2 - Nusa Tenggara	< 0.001	*	0,009	0,027	0,021	0,022	0,012	0,002	0,012	0,032	0,032	0,034	0,018	0,022	0,018	0,017	0,017	0,246	0,137	0,116	0,209	0,311	0,050	0,064	0,103
4 - Java	< 0.001	< 0.001	*	0,030	0,033	0,032	0,012	0,018	0,008	0,024	0,032	0,043	0,014	0,018	0,012	0,012	0,011	0,252	0,155	0,153	0,288	0,501	0,054	0,072	0,118
3 - Bali	< 0.001	< 0.001	< 0.001	*	0,020	0,028	0,019	0,032	0,020	0,043	0,048	0,039	0,027	0,036	0,028	0,023	0,035	0,306	0,192	0,152	0,238	0,359	0,057	0,058	0,096
12 - Philippines	< 0.001	< 0.001	< 0.001	< 0.001	*	0,020	0,011	0,026	0,016	0,044	0,046	0,018	0,027	0,034	0,038	0,034	0,037	0,320	0,204	0,140	0,217	0,355	0,052	0,054	0,080
10 - Sulawesi	< 0.001	< 0.001	< 0.001	< 0.001	< 0.001	*	0,009	0,021	0,021	0,039	0,025	0,048	0,033	0,023	0,040	0,039	0,043	0,308	0,200	0,146	0,234	0,388	0,036	0,079	0,129
9 - Borneo	< 0.001	< 0.001	< 0.001	< 0.001	< 0.001	< 0.001	*	0,011	0,006	0,027	0,020	0,030	0,016	0,012	0,024	0,020	0,022	0,275	0,171	0,132	0,239	0,431	0,037	0,059	0,101
11 - Moluccas	0,126	0,198	0,009	< 0.001	< 0.001	< 0.001	< 0.001	*	0,015	0,036	0,037	0,038	0,026	0,025	0,023	0,020	0,024	0,215	0,117	0,078	0,216	0,445	0,062	0,086	0,121
5 - Sumatra	< 0.001	< 0.001	0,009	< 0.001	< 0.001	< 0.001	0,009	< 0.001	*	0,031	0,026	0,033	0,017	0,016	0,021	0,018	0,020	0,278	0,169	0,138	0,246	0,416	0,041	0,046	0,079
8 - Penins. Malaysia	< 0.001	< 0.001	< 0.001	< 0.001	< 0.001	< 0.001	< 0.001	< 0.001	< 0.001	*	0,047	0,063	0,033	0,033	0,024	0,026	0,023	0,290	0,187	0,168	0,262	0,383	0,065	0,067	0,141
16 - South Korea	< 0.001	< 0.001	< 0.001	< 0.001	< 0.001	< 0.001	< 0.001	< 0.001	< 0.001	< 0.001	*	0,069	0,032	0,003	0,040	0,040	0,040	0,314	0,203	0,173	0,266	0,403	0,043	0,087	0,147
17 - Taiwan	< 0.001	< 0.001	< 0.001	< 0.001	< 0.001	< 0.001	< 0.001	< 0.001	< 0.001	< 0.001	< 0.001	*	0,038	0,051	0,049	0,044	0,047	0,322	0,204	0,136	0,201	0,320	0,075	0,085	0,096
18 - Hainan	< 0.001	< 0.001	< 0.001	< 0.001	< 0.001	< 0.001	< 0.001	< 0.001	< 0.001	< 0.001	< 0.001	< 0.001	*	0,015	0,012	0,009	0,012	0,297	0,189	0,160	0,265	0,421	0,054	0,077	0,120
19 - Mixed Han	< 0.001	< 0.001	< 0.001	< 0.001	< 0.001	< 0.001	< 0.001	< 0.001	< 0.001	< 0.001	< 0.001	< 0.001	< 0.001	*	0,021	0,019	0,019	0,282	0,177	0,155	0,265	0,429	0,040	0,076	0,130
13 - Vietnam	< 0.001	< 0.001	0,009	< 0.001	< 0.001	< 0.001	< 0.001	< 0.001	< 0.001	< 0.001	< 0.001	< 0.001	< 0.001	< 0.001	*	-0,001	0,003	0,287	0,184	0,159	0,278	0,455	0,071	0,087	0,141
14 - Laos	< 0.001	< 0.001	< 0.001	< 0.001	< 0.001	< 0.001	< 0.001	< 0.001	< 0.001	< 0.001	< 0.001	< 0.001	< 0.001	< 0.001	0,838	*	0,002	0,284	0,180	0,152	0,265	0,431	0,064	0,082	0,132
15 - Thailand	< 0.001	< 0.001	0,009	< 0.001	< 0.001	< 0.001	< 0.001	< 0.001	< 0.001	< 0.001	< 0.001	< 0.001	< 0.001	< 0.001	0,117	0,144	*	0,284	0,181	0,160	0,280	0,454	0,063	0,084	0,137
20 - WNG	< 0.001	< 0.001	< 0.001	< 0.001	< 0.001	< 0.001	< 0.001	< 0.001	< 0.001	< 0.001	< 0.001	< 0.001	< 0.001	< 0.001	< 0.001	< 0.001	< 0.001	*	0,042	0,230	0,468	0,583	0,309	0,316	0,350
21 - PNG	< 0.001	< 0.001	< 0.001	< 0.001	< 0.001	< 0.001	< 0.001	< 0.001	< 0.001	< 0.001	< 0.001	< 0.001	< 0.001	< 0.001	< 0.001	< 0.001	< 0.001	< 0.001	*	0,150	0,379	0,521	0,205	0,210	0,242
22 - Admiralty Islands	< 0.001	< 0.001	< 0.001	< 0.001	< 0.001	< 0.001	< 0.001	< 0.001	< 0.001	< 0.001	< 0.001	< 0.001	< 0.001	< 0.001	< 0.001	< 0.001	< 0.001	< 0.001	< 0.001	*	0,097	0,223	0,188	0,205	0,205
23 - Solomons	< 0.001	< 0.001	< 0.001	< 0.001	< 0.001	< 0.001	< 0.001	< 0.001	< 0.001	< 0.001	< 0.001	< 0.001	< 0.001	< 0.001	< 0.001	< 0.001	< 0.001	< 0.001	< 0.001	< 0.001	*	0,049	0,294	0,326	0,289
24 - Polynesia	< 0.001	< 0.001	< 0.001	< 0.001	< 0.001	< 0.001	< 0.001	< 0.001	< 0.001	< 0.001	< 0.001	< 0.001	< 0.001	< 0.001	< 0.001	< 0.001	< 0.001	< 0.001	< 0.001	< 0.001	< 0.001	*	0,501	0,528	0,413
25 - Australia (Indigenous)	< 0.001	< 0.001	< 0.001	< 0.001	< 0.001	< 0.001	< 0.001	< 0.001	< 0.001	< 0.001	< 0.001	< 0.001	< 0.001	< 0.001	< 0.001	< 0.001	< 0.001	< 0.001	< 0.001	< 0.001	< 0.001	< 0.001	*	0,098	0,144
6 - Mentawai	< 0.001	< 0.001	< 0.001	< 0.001	< 0.001	< 0.001	< 0.001	< 0.001	< 0.001	< 0.001	< 0.001	< 0.001	< 0.001	< 0.001	< 0.001	< 0.001	< 0.001	< 0.001	< 0.001	< 0.001	< 0.001	< 0.001	< 0.001	*	0,074
7 - Nias	< 0.001	< 0.001	< 0.001	< 0.001	< 0.001	< 0.001	< 0.001	< 0.001	< 0.001	< 0.001	< 0.001	< 0.001	< 0.001	< 0.001	< 0.001	< 0.001	< 0.001	< 0.001	< 0.001	< 0.001	< 0.001	< 0.001	< 0.001	< 0.001	*

Values above diagonal: population pairwise FST values
Lowest and highest values are highlighted in green and red, resp.
Values below diagonal: p-values (significance level=0,05; 1023 permutations)
East Timor values are highlighted in grey, maximum and minimum vales bold
p value above 0.05 is highlighted in yellow

References for populations: see Table 3

The pairwise F_{ST} values from comparisons including East Timor indicated a limited differentiation from both the Moluccas (0.005) and Nusa Tenggara (0.011), and a pronounced differentiation from Polynesia, WNG and the Solomon Islands at the other end ($F_{ST} \geq 0.21$). Generally high F_{ST} values (>0.1, up to 0.58) were yielded in pairwise comparisons including the latter three populations, PNG, Admiralties, and Nias, most likely due to the broad geographic range of populations included; also Australia and Mentawai showed high values. All other comparisons resulted in a limited differentiation ($F_{ST} \leq 0.05$). For further interpretations, see text.
