# Supplementary material for: Human settlement history between Sunda and Sahul: a focus on East Timor (Timor-Leste) and the Pleistocenic mtDNA diversity
Source: BMC Genomics. 2015 Feb 14;16(1):70. doi: 10.1186/s12864-014-1201-x (PMC4342813; doi:10.1186/s12864-014-1201-x)
Supplement: Additional file 11: — Frequency of selected mtDNA haplogroups in East Timor and surrounding populations. For a graphical representation of frequencies, see Figures 5 and 7 and Additional file 12. [file 12864_2014_1201_MOESM11_ESM.pdf]

Additional file 11: Frequency of selected mtDNA haplogroups in East Timor and surrounding populations

population no.	sample origin	reference(s)	mtDNA sequence range analyzed (indicated in the order of references included)	sample number
1	East Timor	[3]|this study	HVS-I (nps 16022-16392)|CR and WG	**362 (38|324)**
1A	East Timor	this study	CR and WG	**324**
2A	Nusa Tenggara (West Timor)	[5]	HVS-I (nps 16001-16530)	**528**
2B	Nusa Tenggara (Lembata)	[5]|[3]	HVS-I (nps 16001-16530)|HVS-I (nps 16022-16392)	**126 (92|34)**
2C	Nusa Tenggara (Sumba)	[5]|[24]	HVS-I (nps 16001-16530)|HVS-I (nps 16020-16500) **k**	**684 (634|50)**
2D	Nusa Tenggara (Lombok)	[24]	HVS-I (nps 16020-16500) **k**	**44**
2E	Nusa Tenggara (Flores)	[5]|[3]|[24]|[65]	HVS-I (nps 16001-16530)|HVS-I (nps 16022-16392)|HVS-I (nps 16020-16500) k|HVS-I (nps 16017-16420)	**548 (469|75|2|2)**
2F	Nusa Tenggara (Solor)	[3]	HVS-I (nps 16022-16392)	**41**
2G	Nusa Tenggara (Adonara)	[3]	HVS-I (nps 16022-16392)	**75**
2H	Nusa Tenggara (Pantar)	[5]|[3]	HVS-I (nps 16001-16530)|HVS-I (nps 16022-16392)	**67 (29|38)**
2K	Nusa Tenggara (Alor)	[5]|[3]|[24]|[65]	HVS-I (nps 16001-16530)|HVS-I (nps 16022-16392)|HVS-I (nps 16020-16500) **k** |HVS-I (nps 16017-16420)	**103 (23|27|45|8)**
3	Bali	[5]|[24]	HVS-I (nps 16001-16530)|HVS-I (nps 16020-16500) **k**	**570 (487|83)**
4	Java	[5]|[24]	HVS-I (nps 16001-16530)|HVS-I (nps 16020-16500) **k**	**97 (51|46)**
5	Sumatra	[5]|[40] **m**	HVS-I (nps 16001-16530)|HVS-I (nps 16012-16497) **k**	**228 (42|186)**
6	Mentawai	[5]	HVS-I (nps 16001-16530)	**128**
7	Nias	[5]|[41]	HVS-I (nps 16001-16530)|HVS-I (nps 16024-16392)	**499 (59|440)**
8	Peninsular Malaysia	[30]|[138]|[57]|[17]	WG|HVS-I (nps 15997-16410), HVS-II (nps 48-408) and HVS-III (nps 438-619)|HVS-I (nps 16024-16365) and HVS-II (nps 73-340)|HVS-I (average nps 16024-16400)	**781 (63|248|205|265)**
9	Borneo	[24]	HVS-I (nps 16020-16500) **k**	**157**
10	Sulawesi	[5]|[24]	HVS-I (nps 16001-16530)|HVS-I (nps 16020-16500) **k**	**437 (200|237)**
11A	Moluccas (Ternate)	[65]	HVS-I (nps 16017-16420)	**31**
11B	Moluccas (Ambon)	[24]	HVS-I (nps 16020-16500) **k**	**43**
12	Philippines	[55]|[141] f |[28]|[24]	WG|HVS-I (nps 16024-16391)/HVS-I (nps 16032-16365) (and HVS-II (nps 73-340)), WG|HVS-I (nps 16020-16500) **k**	**959 (360|116|422|61)**
13	Vietnam	[80]|[58]	HVS-I (nps 16038-16569) and HVS-II (nps 1-300)|CR	**494 (307|187)**
14	Laos	[59]	CR	**214**
15	Thailand	[60]	CR	**190**
16	South Korea	[61]	CR	**692**
17	Taiwan (indigenous)	[140]|[24]|[45]	CR|HVS-I (nps 16020-16500) **k** |HVS-I (nps 16006-16397) and HVS-II (nps 53-404)	**1257 (539|78|640)**
18	Hainan	[62]	HVS-I (nps 16080-16569) (and HVS-II (minimum nps 1-207, maximum nps 1-575))	**293**
19	China (Mixed Han)	[63]	HVS-I (nps 16001-16497) and HVS-II (nps 30-407)	**262**
20A	West New Guinea (highlands)	[66]	HVS-I (nps 16039-16373)	**106**
20B	West New Guinea (lowlands)	[66]	HVS-I (nps 16039-16373)	**120**
20C	West New Guinea (Southwest and Lowland Riverine)	[44] r	HVS-I and HVS-II (nps 16000-16569,1-408) and WG	**60**
21A	Papua New Guinea (pooled)	[44] r|[52,53]|[136]|[129,132]	HVS-I and HVS-II (nps 16000-16569,1-408) and WG|WG|HVS-I (nps 16018-16390)|HVS-I (nps 16051-16390 and 16067-16361) and HVS-II (nps 43-373 and 64-385)	**287 (171|21|47|48)**
21B	Papua New Guinea (Wewak)	[64]	HVS-I and HVS-II (nps 16001-16569,1-390)	**94**
21C	Papua New Guinea (Bundi)	[34]	HVS-I (nps 16024-16383) and HVS-II (nps 57-302)	**48**
21D	Papua New Guinea (Gidra)	[67]	HVS-I (nps 16007-16569, 1-60)	**59**
21E	Papua New Guinea (East Sepik Province)	[137]	HVS-I (nps 16019-16351)	**219**
21F	Papua New Guinea (Kula Ring)	[86]	HVS-I (nps 16024-16392)	**432**
22	Admiralty Islands	[68]|[67]	HVS-I (nps 16024-16392)|HVS-I (nps 16007-16569, 1-60)	**203 (144|59)**
23	Solomons	[44] s|[29]	HVS-I and HVS-II (nps 16000-16569,1-408)|HVS-I (16024-16365)	**1045 (342|703)**
24	Polynesia/ Fiji	[65]|[67]|[44] r	HVS-I (nps 16017-16420)|HVS-I (nps 16007-16569, 1-60)|HVS-I and HVS-II (nps 16000-16569,1-408)	**409 (351|43|15)**
25A	Australia (Northwest, Northern Territory and Far North Queensland)	[77]|[34]|[44]|[53]|[76]|[128,132]	HVS-I (nps 16050-16385) and HVS-II (nps 1-309)|HVS-I (nps 16024-16383) and HVS-II (nps 57-302)|WG|WG|HVS-I (nps 16050-16400) | HVS-I (nps 16051-16390) and HVS-II (43-373)	**167 (9|32|1|17|54|54)**
25B	Australia (New South Wales, Paakintji and Ngiyambaa)	[69,75]	HVS-I (nps 15997-16377) (and HVS-II (nps 1-577), WG) **n**	**117**
26	Cambodia	[115]|[135]	HVS-I (16038-16462) and HVS-II (nps 65-417) |HVS-I (nps 16024-16400)	**1085 (1054|31)**
27	Myanmar	[116]	CR	**327**
28	New Britain and New Ireland (Bismarck Archipelago)	[44] r	HVS-I and HVS-II (nps 16000-16569,1-408) and WG	**751**
29	Bougainville	[44] r	HVS-I and HVS-II (nps 16000-16569,1-408)	**273**
30	Vanuatu	[130]|[131]|[44] r	HVS-I (nps 16065-16375)|HVS-I (16184-16373)|HVS-I and HVS-II (nps 16000-16569,1-408)	**98 (51|24|23)**
31	New Caledonia	[44] r	HVS-I and HVS-II (nps 16000-16569,1-408)	**25**
32	Micronesia	[133]|[44] r	HVS-I (16184-16373)|HVS-I and HVS-II (nps 16000-16569,1-408)	**585 (538|47)**
33	New Zealand Maori	[139]	WG	**20**

population no.	P1xP1d1	P1d1	P1 (total)	P2 $	P3a	P3b	P4a	P4b	P5	P6	P7	P8	P9 $	P10	unassigned P	PxP1 total	P total
1	18 (5.0%)	0	**18 (5.0%)**	0	0	0 **d**	0	0	0	0	0	0	0	0	0	0	**18 (5.0%)**
1A	14 (4.3%)	0	**14 (4.3%)**	0	0	0	0	0	0	0	0	0	0	0	0	0	**14 (4.3%)**
2A	n.d. **f**	n.d. **f**	**n.d. f**	n.d. **f**	n.d. **f**	n.d. **f,g**	n.d. **f**	n.d. **f**	n.d. **f**	n.d. **f**	n.d. **f**	n.d. **f**	n.d. **f**	n.d. **f**	5 (0.9%) **f**	n.d. **f**	**5 (0.9%) f**
2B	5 (4.0%)	9 (7.1%)	**14 (11.1%)**	n.d.	0	0 **g**	0	0	0	0	0	0	n.d.	0	0	0	**14 (11.1%)**
2C	4 (0.6%)	7 (1.0%)	**11 (1.6%)**	n.d.	0	0 **g**	0	0	0	0	0	0	n.d.	0	0	0	**11 (1.6%)**
2D	0	0	**0**	n.d.	0	0 **g**	0	0	0	0	0	0	n.d.	0	0	0	**0**
2E	9 (1.6%)	10 (1.8%)	**19 (3.5%)**	n.d.	0	0 **g**	6 (1.1%)	0	0	0	0	0	n.d.	0	0	6 (1.1%)	**25 (4.6%)**
2F	0	0	**0**	n.d.	0	0 **g**	1 (2.4%)	0	0	0	0	0	n.d.	0	0	1 (2.4%)	**1 (2.4%)**
2G	0	0	**0**	n.d.	0	0 **g**	0	0	0	0	0	0	n.d.	0	0	0	**0**
2H	0	2 (3.0%)	**2 (3.0%)**	n.d.	0	0 **g**	0	0	0	0	0	0	n.d.	0	0	0	**2 (3.0%)**
2K	0	3 (2.9%)	**3 (2.9%)**	n.d.	0	0 **g**	0	0	0	0	0	0	n.d.	0	0	0	**3 (2.9%)**
3	1 (0.2%)	0	**1 (0.2%)**	n.d.	0	0 **g**	0	0	0	0	0	0	n.d.	0	0	0	**1 (0.2%)**
4	0	0	**0**	n.d.	0	0 **g**	0	0	0	0	0	0	n.d.	0	0	0	**0**
5	0	0	**0**	n.d.	0	0 **g**	0	0	0	0	0	0	n.d.	0	0	0	**0**
6	0	0	**0**	n.d.	0	0 **g**	0	0	0	0	0	0	n.d.	0	0	0	**0**
7	0	0	**0**	n.d.	0	0 **g**	0	0	0	0	0	0	n.d.	0	0	0	**0**
8	0	1 (0.1%)	**1 (0.1%)**	0	0	0 **d**	0	0	0	0	0	0	0	0	0	0	**1 (0.1%)**
9	0	0	**0**	n.d.	0	0 **g**	0	0	0	0	0	0	n.d.	0	0	0	**0**
10	2 (0.5%)	1 (0.2%)	**3 (0.7%)**	n.d.	0	0 **g**	0	0	0	0	0	0	n.d.	0	1 (0.2%)	1 (0.2%)	**4 (0.9%)**
11A	2 (6.5%)	1 (3.2%)	**3 (9.7%)**	n.d.	0	0 **g**	0	0	0	0	0	0	n.d.	0	0	0	**3 (9.7%)**
11B	0	0	**0**	n.d.	0	0 **g**	0	0	0	0	0	0	n.d.	0	0	0	**0**
12	0	1 (0.1%)	**1 (0.1%)**	0	0	0 **d**	0	0	0	0	0	0	13 (1.4%)	9 (0.9%)	29 (3.0%)	51 (5.3%)	**52 (5.4%)**
13	0	0	**0**	n.d.	0	0	0	0	0	0	0	0	n.d.	0	0	0	**0**
14	0	0	**0**	n.d.	0	0	0	0	0	0	0	0	n.d.	0	0	0	**0**
15	0	0	**0**	n.d.	0	0	0	0	0	0	0	0	n.d.	0	0	0	**0**
16	0	0	**0**	n.d.	0	0	0	0	0	0	0	0	n.d	0	0	0	**0**
17	0	0	**0**	n.d.	0	0	0	0	0	0	0	0	n.d. **c**	0	1 (0.1%)	1 (0.1%)	**1 (0.1%)**
18	0	0	**0**	n.d.	0	0	0	0	0	0	0	0	n.d.	0	0	0	**0**
19	0	0	**0**	n.d.	0	0	0	0	0	0	0	0	n.d.	0	0	0	**0**
20A	15 (14.2%)	22 (20.8%)	**37 (35.0%)**	n.d.	0	0 **g**	1 (0.9%)	0	0	0	0	0	n.d.	0	0	1 (0.9%)	**38 (35.8%)**
20B	9 (7.5%)	7 (5.8%)	**16 (13.3%)**	n.d.	1 (0.8%)	0 **g**	0	0	0	0	0	0	n.d	0	2 (1.7%)	3 (2.5%)	**19 (15.8%)**
20C	10 (16.7%)	n.d. **f**	**10 (16.7%)**	0	0	1 (1.7%)	1 (1.7%)	0	0	0	0	0	0	0	6 (10.0%)	8 (13.3%)	**18 (30.0%)**
21A	54 (18.8%)	4 (1.4%)	**58 (20.2%)**	3 (1.0%)	0	2 (0.7%) **d**	17 (5.9%)	0	0	0	0	0	0 **c**	0	21 (7.3%)	43 (15.0%)	**101 (35.2%)**
21B	20 (21.3%)	11 (11.7%)	**31 (33.0%)**	n.d.	0	0	0	0	0	0	0	0	n.d	0	1 (1.1%)	1 (1.1%)	**32 (34.0%)**
21C	16 (33.3%)	5 (10.4%)	**21 (43.8%)**	n.d.	0	0	1 (2.1%)	0	0	0	0	0	n.d **c**	0	5 (10.4%)	6 (12.5%)	**27 (56.3%)**
21D	9 (15.2%)	3 (5.1%)	**12 (20.3%)**	n.d.	0	0 **g**	0	0	0	0	0	0	n.d	0	0	0	**12 (20.3%)**
21E	29 (13.2%)	14 (6.4%)	**43 (19.6%)**	n.d.	0	0 **g**	0	0	0	0	0	0	n.d	0	0	0	**43 (19.6%)**
21F	73 (16.9%)	4 (0.9%)	**77 (17.8%)**	n.d.	0	0 **g**	0	0	0	0	0	0	0	0	85 (19.7%)	85 (19.7%)	**162 (37.5%)**
22	2 (1.0%)	0	**2 (1.0%)**	n.d.	0	0 **g**	2 (1.0%)	0	0	0	0	0	n.d	0	6 (2.9%)	8 (3.9%)	**10 (4.9%)**
23	18 (1.7%)	0	**18 (1.7%)**	n.d.	0	0	1 (0.1%)	0	0	0	0	0	n.d.	0	0	1 (0.1%)	**19 (1.8%)**
24	4 (1.0%)	0	**4 (1.0%)**	n.d.	0	0	0	0	0	0	0	0	n.d	0	1 (0.2%)	1 (0.2%)	**5 (1.2%)**
25A	0	1 (0.6%)	**1 (0.6%)**	0	7 (4.2%)	2 (1.2%) **d**	0	4 (2.4%)	7 (4.2%)	2 (1.2%)	1 (0.6%)	8 (4.8%)	0	0	4 (2.4%)	35 (21.0%)	**36 (21.6%)**
25B	0	0	**0**	0	0	0	0	22 (18.8%)	0	0	0	8 (6.8%)	n.d	0	0	30 (25.6%)	**30 (25.6%)**
26	0	0	**0**	n.d	0	0	0	0	0	0	0	0	n.d	0	0	0	**0**
27	0	0	**0**	n.d	0	0	0	0	0	0	0	0	n.d	0	0	0	**0**
28	24 (3.2%)	n.d. **f**	**24 (3.2%)**	0	0	0	3 (0.4%)	0	0	0	0	0	0	0	2 (0.3%)	5 (0.7%)	**29 (3.9%)**
29	8 (2.9%)	n.d. **f**	**8 (2.9%)**	n.d.	0	0	0	0	0	0	0	0	n.d.	0	0	0	**8 (2.9%)**
30	11 (11.2%)	3 (3.1%)	**14 (14.3%)**	n.d.	0	0	0	0	0	0	0	0	n.d	0	0	0	**14 (14.3%)**
31	2 (8.0%)	n.d. **f**	**2 (8.0%)**	n.d.	0	0	0	0	0	0	0	0	n.d	0	8 (32.0%)	8 (32.0%)	**10 (40.0%)**
32	0	46 (7.9%)	**46 (7.9%)**	n.d.	0	0	0	0	0	0	0	0	n.d	0	0	0	**46 (7.9%)**
33	0	0	**0**	0	0	0	0	0	0	0	0	0	0	0	0	0	**0**

population no.	Q1	Q2	Q3 #	unassigned Q §	Q total	P and Q total	N21	M7c1(c3)	F1a3a	F1a4a1	D5	F3b	F1a1	B4c2	E1a	E1b	E2
1	55 (15.2%)	0	1 (0.3%) **e**	0	**56 (15.5%)**	74 (20.4%)	2 (0.6%)	45 (12.4%)	16 (4.4%)	31 (8.6%)	5 (1.4%)	1 (0.3%)	1 (0.3%)	2 (0.6%)	54 (14.9%)	8 (2.2%)	8 (2.2%)
1A	46 (14.2%)	0	1 (0.3%) **e**	0	**47 (14.5%)**	61 (18.8%)	2 (0.6%)	45 (13.9%)	16 (4.9%)	29 (9.0%)	5 (1.5%)	1 (0.3%)	1 (0.3%)	2 (0.6%)	44 (13.6%)	7 (2.2%)	7 (2.2%)
2A	74 (14.0%)	0	0 **h**	5 (0.9%) **f**	**79 (15.0%)**	84 (15.9%)	0	31 (5.9%)	15 (2.8%)	102 (19.3%)	14 (2.7%)	4 (0.8%) **f,t**	0	3 (0.6%)	29 (5.5%)	34 (6.4%)	2 (0.4%)
2B	11 (8.7%)	5 (4.0%)	0	0	**16 (12.7%)**	30 (23.8%)	0	8 (6.3%)	5 (4.0%)	15 (11.9%)	5 (4.0%)	0	3 (2.4%)	0	10 (7.9%)	1 (0.8%)	0
2C	2 (0.3%)	0	0	0	**2 (0.3%)**	13 (1.9%)	0	76 (11.1%)	37 (5.4%)	34 (5.0%)	36 (5.3%)	7 (1.0%)	11 (1.6%)	1 (0.2%)	11 (1.6%)	22 (3.2%)	12 (1.8%)
2D	0	0	0	0	**0**	0	0	1 (2.3%)	1 (2.3%)	1 (2.3%)	1 (2.3%)	0	4 (9.1%)	0	1 (2.3%)	3 (6.8%)	0
2E	33 (6.0%)	2 (0.4%)	0	0	**35 (6.4%)**	60 (10.9%)	0	28 (5.1%)	25 (4.6%)	25 (4.6%)	18 (3.3%)	0	12 (2.2%)	6 (1.1%)	14 (2.6%)	6 (1.1%)	14 (2.6%)
2F	3 (7.3%)	1 (2.4%)	0	0	**4 (9.8%)**	5 (12.2%)	0	2 (4.9%)	0	4 (9.8%)	1 (2.4%)	0	0	0	0	0	5 (12.2%)
2G	8 (10.7%)	4 (5.3%)	0	0	**12 (16.0%)**	12 (16.0%)	0	5 (6.7%)	7 (9.3%)	9 (12.0%)	0	0	2 (2.7%)	0	0	1 (1.3%)	1 (1.3%)
2H	22 (32.8%)	0	0	0	**22 (32.8%)**	24 (35.8%)	0	4 (6.0%)	1 (1.5%)	1 (1.5%)	0	0	0	0	2 (3.0%)	2 (3.0%)	0
2K	22 (21.4%)	0	0 **h**	1 (1.0%)	**23 (22.3%)**	26 (25.2%)	3 (2.9%)	4 (3.9%)	0	9 (8.7%)	0	0	1 (1.0%)	0	7 (6.8%)	4 (3.9%)	0
3	1 (0.2%)	0	0 **h**	3 (0.5%)	**4 (0.7%)**	5 (0.9%)	10 (1.8%)	20 (3.5%)	12 (2.1%)	7 (1.2%)	1 (0.2%)	0	14 (2.5%)	40 (7.0%)	14 (2.5%)	4 (0.7%)	5 (0.9%)
4	0	0	0	0	**0**	0	0	7 (7.2%)	2 (2.1%)	0	1 (1.0%)	0	7 (7.2%)	1 (1.0%)	0	1 (1.0%)	2 (2.1%)
5	0	0	0	0	**0**	0	1 (0.4%)	18 (7.9%)	2 (0.9%)	2 (0.9%)	1 (0.4%)	0	10 (4.4%)	4 (1.8%)	6 (2.6%)	4 (1.8%)	1 (0.4%)
6	0	0	0	0	**0**	0	0	32 (25.0%)	0	0	1 (0.8%)	0	0	0	0	4 (3.1%)	0
7	0	0	0	0	**0**	0	0	42 (8.4%)	0	0	32 (6.4%)	6 (1.2%)	10 (2.0%)	1 (0.2%)	1 (0.2%)	3 (0.6%)	0
8	1 (0.1%)	0	0	0	**1 (0.1%)**	2 (0.3%)	33 (4.2%)	39 (5.0%)	5 (0.6%)	4 (0.5%)	4 (0.5%)	2 (0.3%)	58 (7.4%)	9 (1.2%)	9 (1.2%)	10 (1.3%)	4 (0.5%)
9	0	0	0 **h**	2 (1.3%)	**2 (1.3%)**	2 (1.3%)	0	11 (7.0%)	3 (1.9%)	3 (1.9%)	7 (4.5%)	9 (5.7%)	3 (1.9%)	9 (5.7%)	14 (8.9%)	3 (1.9%)	5 (3.2%)
10	3 (0.7%)	0	0 **h**	5 (1.1%)	**8 (1.8%)**	12 (2.7%)	1 (0.2%)	40 (9.2%)	8 (1.8%)	17 (3.9%)	23 (5.3%)	0	5 (1.1%)	2 (0.5%)	76 (17.4%)	22 (5.0%)	18 (4.1%)
11A	2 (6.5%)	0	0	0	**2 (6.5%)**	5 (16.1%)	0	3 (9.7%)	5 (16.1%)	0	0	0	0	0	1 (3.2%)	0	3 (9.7%)
11B	0	0	0 **h**	5 (11.6%)	**5 (11.6%)**	5 (11.6%)	0	1 (2.3%)	3 (7.0%)	2 (4.7%)	1 (2.3%)	0	1 (2.3%)	0	2 (4.7%)	0	2 (4.7%)
12	0	0	0	1 (0.1%)	**1 (0.1%)**	53 (5.5%)	0	126 (13.1%)	38 (4.0%)	30 (3.1%)	7 (0.7%)	28 (2.9%)	1 (0.1%)	0	87 (9.1%)	7 (0.7%)	23 (2.4%)
13	0	0	0	0	**0**	0	3 (0.6%)	2 (0.4%)	1 (0.2%)	1 (0.2%)	5 (1.0%)	0	28 (5.7%)	22 (4.5%)	1 (0.2%)	0	1 (0.2%)
14	0	0	0	0	**0**	0	2 (0.9%)	0	0	0	2 (0.9%)	0	27 (12.6%)	6 (2.8%)	0	0	0
15	0	0	0	0	**0**	0	0	0	0	1 (0.5%)	2 (1.1%)	0	17 (8.9%)	3 (1.6%)	0	0	0
16	0	0	0	0	**0**	0	0	0	6 (0.9%)	0	3 (0.4%)	0	12 (1.7%)	0	0	0	0
17	0	0	0	0	**0**	1 (0.1%)	0	64 (5.1%)	9 (0.7%)	8 (0.6%)	52 (4.1%)	61 (4.9%)	49 (3.9%)	2 (0.2%)	61 (4.9%)	3 (0.2%)	13 (1.0%)
18	0	0	0	0	**0**	0	0	2 (0.7%)	0	3 (1.0%)	13 (4.4%)	0	32 (10.9%)	0	0	0	0
19	0	0	0	0	**0**	0	0	0	1 (0.4%)	1 (0.4%)	17 (6.5%)	0	8 (3.1%)	0	0	0	0
20A	57 (53.8%)	0	2 (1.9%) **a**	1 (0.9%)	**60 (56.6%)**	98 (92.5%)	0	0	0	0	0	0	0	0	0	0	0
20B	85 (70.8%)	0	0 **h**	1 (0.8%)	**86 (71.7%)**	105 (87.5%)	0	0	0	0	0	0	0	0	0	0	0
20C	41 (68.3%)	0	0	0	**41 (68.3%)**	59 (98.3%)	0	0	0	0	0	0	0	0	0	0	0
21A	46 (16.0%)	13 (4.5%)	11 (3.8%) **a,b**	0	**70 (24.4%)**	171 (59.6%)	0	0	0	1 (0.3%)	0	0	0	0	5 (1.7%)	1 (0.3%)	1 (0.3%)
21B	44 (46.8%)	0	14 (14.9%) **a**	0	**58 (61.7%)**	90 (95.7%)	0	0	0	0	0	0	0	0	0	0	0
21C	14 (29.2%)	0	0	0	**14 (29.2%)**	41 (85.4%)	0	0	0	0	0	0	0	0	0	0	0
21D	18 (30.5%)	0	0 **h**	17 (28.8%)	**35 (59.3%)**	47 (79.7%)	0	0	0	0	0	0	0	0	0	0	0
21E	123 (56.2%)	0	1 (0.5%) **b**	13 (5.9%)	**137 (62.6%)**	180 (82.2%)	0	0	0	0	0	0	0	0	n.d. **f**	n.d. **f**	3 (1.4%)
21F	24 (5.6%)	11 (2.5%)	0	9 (2.1%)	**44 (10.2%)**	206 (47.7 %)	0	0	13 (3.0%)	0	0	0	0	0	0	36 (8.3%)	17 (3.9%)
22	44 (21.7%)	13 (6.4%)	0 **h**	9 (4.4%)	**66 (32.5%)**	76 (37.4%)	0	2 (1.0%)	0	0	0	0	0	0	0	2 (1.0%)	0
23	60 (5.7%)	17 (1.6%)	0	0	**77 (7.4%)**	96 (9.2%)	0	10 (1.0%)	0	0	0	0	0	0	0	0	0
24	9 (2.2%)	8 (2.0%)	0	0	**17 (4.2%)**	22 (5.4%)	0	1 (0.2%)	0	0	0	0	0	0	0	0	0
25A	0	1 (0.6%)	0	0	**1 (0.6%)**	37 (22.2%)	0	0	0	0	0	0	0	0	0	0	0
25B	0	0	0	0	**0**	30 (25.6%)	0	0	0	0	0	0	0	0	0	0	0
26	0	0	0	0	**0**	0	33 (3.0%)	0	1 (0.1%)	0	13 (1.2%)	0	157 (14.5%)	58 (5.3%)	0	0	0
27	0	0	0	0	**0**	0	0	0	0	0	0	0	54 (16.5%)	3 (0.9%)	0	0	0
28	75 (10.0%)	72 (9.6%)	5 (0.7%)	0	**152 (20.2%)**	181 (21.1%)	n.d. **f**	n.d. **f**	n.d. **f**	n.d. **f**	n.d. **f**	n.d. **f**	n.d. **f**	n.d. **f**	7 (0.9%) **f**	64 (8.5%) **f**	1 (0.1%) **f**
29	65 (23.8%)	0	0	0	**65 (23.8%)**	73 (26.7%)	n.d. **f**	n.d. **f**	n.d. **f**	n.d. **f**	n.d. **f**	n.d. **f**	n.d. **f**	n.d. **f**	0 **f**	6 (2.2%) f	0 **f**
30	6 (6.1%)	2 (2.0%)	0	7 (7.1%)	**15 (15.3%)**	29 (29.6%)	0	0	0	0	0	0	0	0	0	0	0
31	0	1 (4.0%)	0	0	**1 (4.0%)**	11 (44.0%)	n.d. **f**	n.d. **f**	n.d. **f**	n.d. **f**	n.d. **f**	n.d. **f**	n.d. **f**	n.d. **f**	0 **f**	0 **f**	0 **f**
32	10 (1.7%)	1 (0.2%)	0	0	**11 (1.9%)**	57 (9.7%)	0	17 (2.9%) **p**	1 (0.2%)	2 (0.3%)	0	0	n.d. **q**	0	0	0	0
33	0	0	0	0	**0**	0	0	0	0	0	0	0	0	0	0	0	0

Haplogroups according to Phylotree [49], build 16
CR = control region (nps 16024-16569, 1-576)
WG = whole mitogenome (nps 1-16569)
n.d. = not detectable in the available sequenced mtDNA range
P1xP1d1 = P1 haplotypes except P1d1
PxP1 = P haplotypes except P1
unassigned P/Q haplotypes could not further be specified in the range analyzed or given in publication

Footnotes (single bold characters in table):

$ P2 and P9 carry no CR polymorphisms; thus frequencies indicated derive from the WG samples
# Q3 carries no CR polymorphisms, HVS-I allows Q3a1 and Q3b detection, HVS-II is neded for Q3a; thus frequencies indicated as detecta
§ transitions at nps 16223, (16311), 16129 and 16241 indicating haplogroup Q status
a Q3a
b Q3b
c haplotypes with P9 CR pattern listed until phylotree [49], build 15 present in sample set
d only detectable in samples with HVS-II information
e Q3*
f no sequence data/information available in publication; if frequencies are indicated: as given in publication
g P3b1 (P3b n.d. in the available sequenced mtDNA range)
h no Q3a1/Q3b contained, whereas Q3a is n.d. in the available sequenced mtDNA range
k average length sequenced according to [24]
m 94 Sumatrans also used in [17]
n some samples were also sequenced in HVS-II and WG. It is not clear which were chosen from identical haplotypes; the results were assumed for all identical HVS-I haplotypes of [69]
p transitions at nps 16223, 16295, 16362 indicating haplogroup M7c1 status
q in the available sequenced mtDNA range [133]
r [44] included data from [48] that were not fully discernible. We used only [44] data to avoid multiple counting of samples
s [44] included data from [48] and [134] that were not fully discernible. We used only [44] data to avoid multiple counting of samples
t F3
