# Supplementary material for: Human settlement history between Sunda and Sahul: a focus on East Timor (Timor-Leste) and the Pleistocenic mtDNA diversity
Source: BMC Genomics. 2015 Feb 14;16(1):70. doi: 10.1186/s12864-014-1201-x (PMC4342813; doi:10.1186/s12864-014-1201-x)
Supplement: Additional file 12: — Dispersal of selected mtDNA haplogroups in East Timor and surrounding populations. (A) Haplogroups related to the “out of Taiwan” dispersal. (B) Haplogroups B4c2 and E; (C) Haplogroup F1a1. All available information was considered (reading frames varied between publications). Frequencies in populations are indicated by colored circle segments. See legends for color codes. Population reference numbers located within a circle indicate that the analyzed haplogroup(s) were not found in this population. For the populations included, see Figure 5. For details and references, see Additional file 11. [file 12864_2014_1201_MOESM12_ESM.pdf]

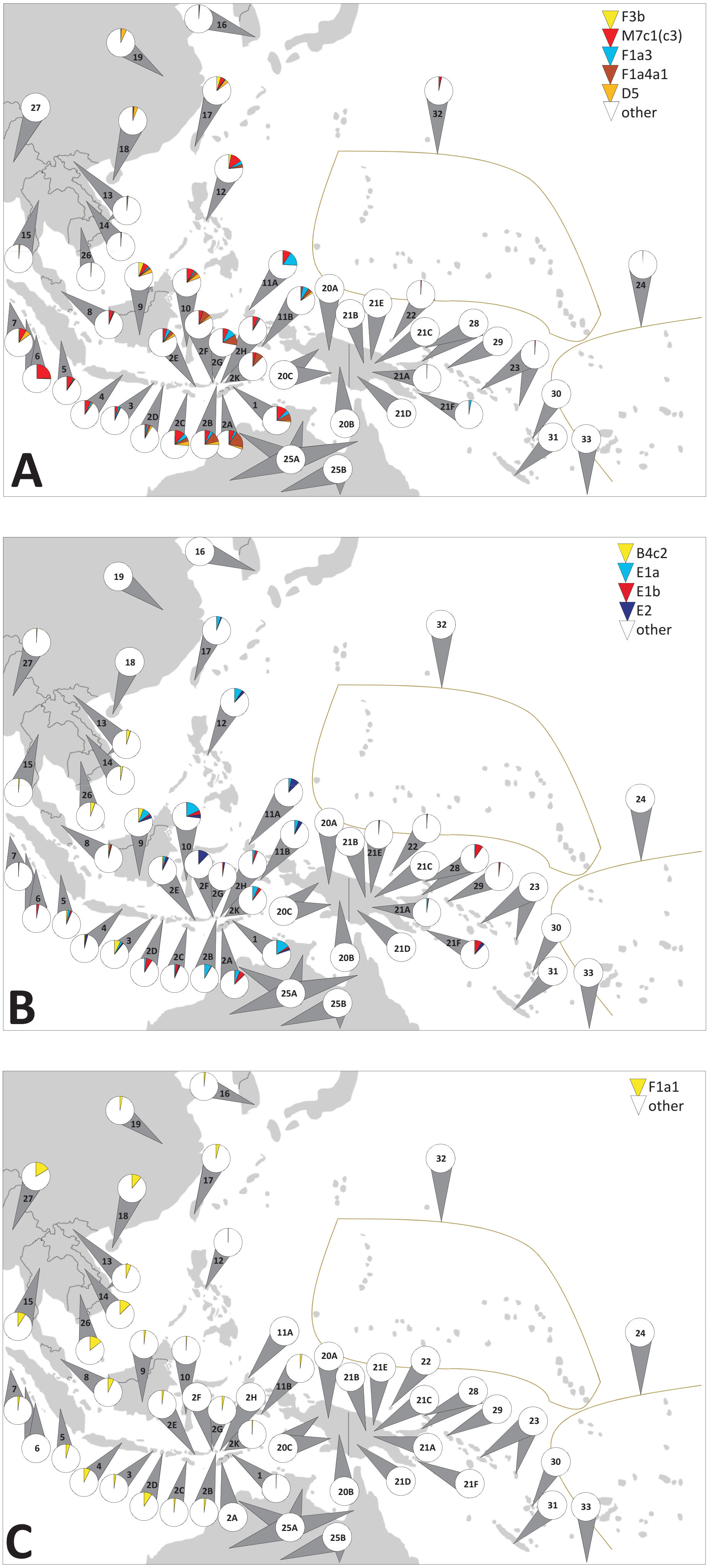
A
F3b
M7c1(c3)
F1a3
F1a4a1
D5
other
B
B4c2
E1a
E1b
E2
other
C
F1a1
other

Comment on 12A:

Haplogroup M7c3c of Phylotree [49], build 3-15, called M7c1c until build 2, is the main marker for the postulated "out of Taiwan" event. In the most recent mtDNA phylogeny (Phylotree build 16), M7c3c has been dissolved from the CR point of view; the defining mitogenomes of build 15 were relabeled M7c1c3, while another closely related haplogroup with identical CR pattern, M7c1a4a, has been introduced. We consequently assign our samples to the MRCA haplogroup M7c1 but treat them, for the lack of more information, as a homogenous group comprising the M7c3c haplotypes described in earlier publications.
